# Integrating Untargeted GC-MS Metabolomics, GNPS Molecular Networking, and Machine Learning for Sugarcane Byproduct Valorization

**DOI:** 10.1021/acsomega.6c00047

**Published:** 2026-04-06

**Authors:** Thapanee Pruksatrakul, Chanakarn Sangsum, Sasina Makmai, Pornkanok Pongpamorn, Atchara Paemanee, Surachet Soontontaweesub, Seangaroon Yoiprommarat, Nantirat Sittichok, Nopparat Suriyachai, Suchat Pongchaiphol, Marisa Raita, Walaiporn Rungjang, Taridaporn Bunyapaiboonsri, Verawat Champreda

**Affiliations:** † National Center for Genetic Engineering and Biotechnology (BIOTEC), National Science and Technology Development Agency (NSTDA), Pathum Thani 12120, Thailand; ‡ The Joint Graduate School for Energy and Environment (JGSEE), King Mongkut’s University of Technology Thonburi, Bangkok 10140, Thailand; § Innovation and Research Development Institute, Mitr Phol Sugarcane Research Center Co., Ltd., Phu Khiao, Chaiyaphum 36110, Thailand

## Abstract

The global sugar industry generates substantial byproducts and waste, posing significant environmental challenges. In alignment with circular economy and zero-waste principles, this study explores the valorization of sugarcane residues through solvent-based extraction and advanced metabolomic profiling. Various organic solvents were employed to extract metabolites from different sugarcane parts, followed by *N*,*O*-bis­(trimethylsilyl)­trifluoroacetamide (BSTFA) derivatization and analysis using gas chromatography-mass spectrometry (GC-MS). Automated spectral deconvolution and molecular networking, conducted via open-source platforms (MSHub, GNPS, and Cytoscape), enabled structural dereplication and clustering of metabolite spectra. Integration of metabolomic data with sample metadata facilitated system-level comparisons of chemical diversity and metabolite abundance across extraction conditions. Machine learning techniques, particularly random forest and multivariate statistical analyses, were applied to the metabolomic data set. These approaches enabled the identification of chemotypic drivers responsible for differentiating biomass types and extraction solvent systems. Results revealed that specific solvent–biomass pairings significantly influenced both the yield and specificity of high-value compounds, including policosanols, phytosterols, triterpenoids, and phenolic acids. Notably, trash and filter cake emerged as promising matrices for lipid-based compound recovery. Methanol and ethanol provided the highest overall extraction efficiency, whereas nonpolar solvents such as *tert*-butyl methyl ether (TBME) and hexane enabled selective enrichment of sterols and long-chain alcohols. By integrating molecular data with statistical modeling and yield analysis, this study presents a data-driven framework for optimizing biorefinery processes. These findings offer critical insights into aligning solvent systems with specific biomass types to enhance the efficiency, economic value, and environmental sustainability of sugarcane residue valorization.

## Graphical abstract

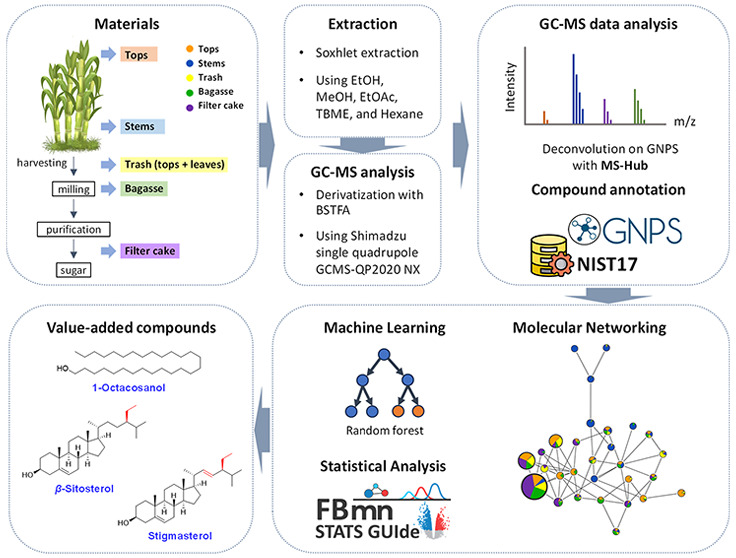

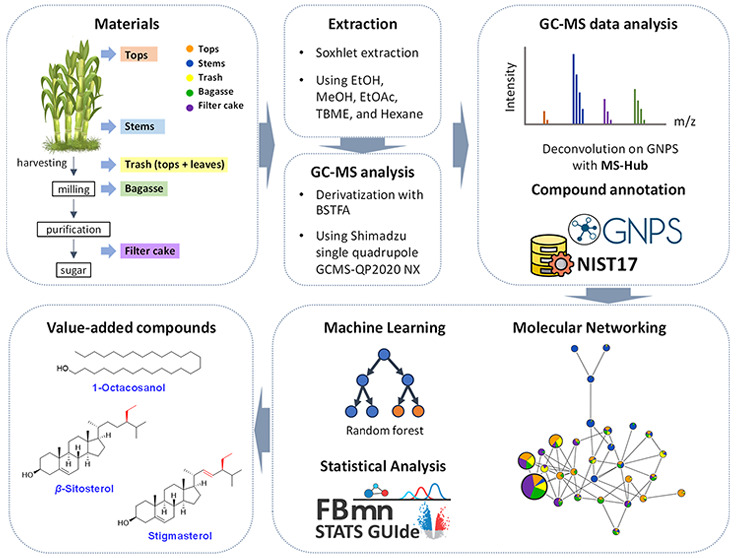

## 1 Introduction

Sugarcane, *Saccharum* spp., is a perennial grass belonging to the Poaceae family, indigenous to South Asia, Southeast Asia, and New Guinea. It is well adapted to tropical and subtropical environments.^1^ Globally, approximately 80% of sugar consumption is derived from sugarcane, with total production reaching 2.03 billion tons cultivated across 27.0 million hectares in 2023.^2^ Sugarcane is one of the key economic crops in Thailand, which ranks among the top 5 global producers and exporters of sugarcane and raw sugar. In 2023, the country produced approximately 94 million tons of sugarcane from 1.6 million hectares of cultivated land.^2^ While sugar production plays a key economic role, it also generates a substantial quantity of biomass that requires improved management and presents opportunities for value-added applications.

Sugarcane processing generates four main categories of byproducts: (1) green tops and dry leaves, (2) bagasse, (3) filter cake (press mud), and (4) molasses.^3^ While green tops and dry leaves result from harvesting, the remaining byproducts originate from milling and refining processes. These operations produce significant amounts of solid and liquid waste annually.^4^ Moreover, preharvest sugarcane burning poses serious environmental and public health risks,^5^ particularly due to the release of fine particulate matter (PM2.5). Valorizing these byproducts provides a sustainable approach for waste utilization and helps reduce the negative impacts of open burning, which aligns with the principles of the circular economy.^6^

Several chemical compounds derived from sugarcane have demonstrated high potency for bioeconomic applications and therapeutic benefits, including policosanol, phenolics, phytosterols, and triterpenoids.^7,8^ Policosanol, a class of long-chain aliphatic alcohols (C_24_–C_34_), exhibited antihyperlipidemic and antihypercholesterolemic effects by lowering of plasma total cholesterol (TC) and low-density lipoprotein cholesterol (LDL-C) levels. Additionally, it shows promising antiproliferative and antithrombotic activities.^7,8^ Phenolics represent another class of bioactive compounds that have attracted significant interest in cosmeceutical, nutraceutical, and pharmaceutical applications,^9,10^ owing to their wide range of therapeutic properties, such as antioxidant, antiallergic, anti-inflammatory, antiproliferative, antifibrotic, antiviral and anticancer effects.^7,8^ In sugarcane, phenolic compounds are categorized into phenolic acids and flavonoids. Phenolic acids include hydroxybenzoic acid (e.g., gallic acid and vanillic acid) and hydroxycinnamic acid (e.g., caffeic acid, sinapic acid, ferulic acid, and *p*-coumaric acid). Flavonoids are subdivided into isoflavone (e.g., genistein), flavonol (e.g., quercetin), flavone (e.g., luteolin and apigenin, and tricin derivatives), and flavone glycosides (e.g., vitexin, orientin, schaftoside, and isochaftoside).^7^ In addition, another important group of bioactive compounds in plants are phytosterols, which are triterpene analogs of cholesterol. These compounds, including stigmasterol, β-sitosterol, and campesterol^11^ are naturally occurring and have been associated with various health benefits. Phytosterols decrease intestinal cholesterol absorption, leading to lower blood cholesterol levels and a reduction in LDL-C.^11–13^ Furthermore, phytosterols have shown positive effects on insulin resistance, lipid metabolism, cancer, Alzheimer disease, and cardiovascular diseases related to atherosclerosis.^11^

In previous studies, gas chromatography (GC) and liquid chromatography (LC) have identified a wide range of phytochemicals in sugarcane. Gas chromatography coupled with mass spectrometry (GC-MS) has revealed the presence of fatty acids, policosanols, long-chain aldehydes, alkanes, phenolics, phytosterols, and triterpenoids in the natural wax composition of sugarcane.^14–16^ Additionally, liquid chromatography-tandem mass spectrometry (LC-MS/MS)-based metabolomics approaches have been applied for comprehensive plant metabolites analysis.^10,17,18^ Using the latter approach for chemical profiling of sugarcane juice and its byproduct molasses, flavone glycosides were found to be the predominant compounds among 42 metabolites, which included flavonoid glycosides, sterols, and fatty acids, detected via ultraperformance liquid chromatography-mass spectrometry (UPLC-MS). Gas chromatography–mass spectrometry (GC-MS) identified sucrose as the major component among 32 metabolites, including ethylene glycol, inorganic acids, nitrogenous compounds, organic acids, and other sugars. Furthermore, NMR spectroscopy detected 13 metabolites, comprising amino acids, organic acids, sugars, phenolic acids, fatty acids, and flavones.^18^

The computational metabolomics approach is widely used to find comprehensive secondary metabolites in plants.^19^ GNPS (global natural products social molecular networking) is a web-based platform that supports the community-wide deposition, processing, sharing, analysis, and curation of mass spectrometry data.^20,21^ It facilitates the annotation of untargeted small molecules and enables the visualization of the chemical space and molecular networks based on spectral similarity. This method involves clustering, aligning, and querying fragmentation spectra of ionized molecules from complex mixtures against curated spectral library databases.^21^ Advanced algorithms and software tools assist in annotating known and structurally related compounds, thereby constructing molecular networks that reflect the chemical relationships. In this study, five sugarcane residues (leaves, tops, trash, bagasse, and filter cake) were extracted using five different solvents [ethanol (EtOH), methanol (MeOH), *tert*-butyl methyl ether (TBME), ethyl acetate (EtOAc), and hexane] prior to GC-MS analysis. The resulting data were processed using a computational metabolomics workflow on the GNPS platform, employing feature-based molecular networking with statistical analysis (FBMN-STAT). Multivariate analysis and the supervised machine learning approach, random forest, were employed to identify key compounds that most significantly distinguished the sugarcane residues based on their GC-MS metabolite profiles. GC-MS-based molecular networking facilitated the visualization of phytochemical profiles across different sugarcane extracts, enabling broad metabolite coverage and revealing their interrelationships. This approach supports the identification and interpretation of compounds with value-added potential and prospective applications in cosmetics, nutraceuticals, and dietary supplement industries.

## 2 Experimental Section

### 2.1 Plant Materials

Sugarcane wastes including bagasse, filter cake, leaves, tops, and trash were obtained from Mitr Phol Sugarcane Research Center Co., Ltd. These raw materials were used for extraction experiments, and a summary of the sugarcane parts and their characteristics is provided in Table 1.

**Table 1:** Details of Sugarcane Materials

type of material	sugarcane variety	cultivated area or processing area	type of crop	cultivation period
tops	U-Thong 12	Ban Nong Phai Nuea, Wang Hin Lat, Chum Phae, Khon Kaen, Thailand	second Ratoon crop (regrowth, third harvest)	Dec 2020–Aug 2021 (8 months)
stems	Khon Kaen 3	Ban Kok Charoen Chai, Kok Sa-at, Phu Khiao, Chaiyaphum, Thailand	second Ratoon crop (regrowth, third harvest)	Dec 2020–Aug 2021 (8 months)
trash	Khon Kaen 3	Ban Kut Chok, Kok Sa-at, Phu Khiao, Chaiyaphum, Thailand	plant crop (first harvest)	Dec 2020–Dec 2021 (12 months)
bagasse	Khon Kaen 3, Phu Khiao 1, Phu Khiao 2, and Lampang 92-11	Mitr Phol Sugar Factory, Phu Khiao, Chaiyaphum, Thailand	no data	2020–2021
filter cake	Khon Kaen 3 and U-Thong 12	Phu Khiao, Chaiyaphum, Thailand	plant crop (first harvest), and first and second Ratoon crops (regrowth; second, and third years)	Dec 2020–Dec 2021 (12 months)

### 2.2 Extraction Procedure

The leaves, tops, trash, bagasse, and filter cake of sugarcane were air-dried and subsequently oven-dried at 60 °C for 24 h. A 10-g sample, ground to a particle size of 1 mm, was placed in a Soxhlet thimble and inserted into a Soxhlet apparatus, connected to a round-bottom flask containing 250 mL of one organic solvent at a time, systematically varying among hexane, *tert*-butyl methyl ether (TBME or MTBE), ethyl acetate (EtOAc), ethanol (EtOH), and methanol (MeOH). Each solvent was refluxed for 5 h individually. Afterward, the extract was concentrated under reduced pressure to yield the crude extract for each solvent.

The same extraction method was adapted for the stems of sugarcane with some modifications. The stems were ground to a particle size of 5 mm and washed several times with warm reverse osmosis water (60–65 °C) at a ratio of 1:10 (1 g of sample to 10 mL of water) at room temperature. This washing continued until there was no color change observed in Benedict’s solution. After filtration, the solid residue was oven-dried at 60 °C for 12 h, then ground to a 1 mm particle size. The Soxhlet extraction was then performed as described for the other sugarcane materials

### 2.3 GC-MS Analysis

To the dried crude extract (6 mg), 80 μL of methoxyamination reagent (20 mg/mL methoxyamine hydrochloride in pyridine) was added. The mixture was shaken at 1100 rpm in a block heater and incubated at 37 °C for 2 h. Thereafter, 140 μL of *N*,*O*-bis­(trimethylsilyl)­trifluoroacetamide (BSTFA) was added, and the mixture was shaken and incubated at 37 °C for an additional 30 min. After incubation, the mixture was centrifuged at 11,000 rpm (11,363 rcf) for 5 min. The supernatant was then transferred for GC-MS analysis using a GCMS-QP2020 NX (Shimadzu Co., Japan), equipped with an SH-Rxi-5Sil MS column (0.25 μm film thickness, 0.25 mm ID, 30 m length). Helium (99.9%) served as the carrier gas at a flow rate of 1 mL/min. A sample volume of 0.2 μL was injected in split mode with a split ratio of 1:10. The ion source and interface temperatures of the mass spectrometer, as well as the injector temperature, were maintained at 250 °C. Mass spectra were obtained by electron ionization (EI) at 70 eV, with a mass scan range of *m*/*z* 45–700, a scan speed of 2500, and an event time of 0.30 s. The column oven temperature program was set as follows: starting at 60 °C (held for 3 min), then increasing at 15 °C/min to 180 °C, followed by an increase at 8 °C/min to 300 °C (held for 5 min), and finally raising at 10 °C/min to 310 °C (held for 8 min). Replicate injections (*n* = 3) were performed, yielding relative standard deviation (% RSD) values below 10%. Metabolite abundances were calculated from peak areas normalized to the internal standard.

Compound identification was carried out by matching the mass spectra with the NIST 17 database (spectral similarity >80%) and by comparison with authentic standards. The reference standards included palmitic acid, stearic acid, *n*-octacosanoic acid, *n*-triacontanoic acid, *p*-coumaric acid, chlorogenic acid, *n*-octacosanol, *n*-triacontanol, *n*-tetracosanol, stigmasterol, and β-sitosterol, all purchased from Sigma-Aldrich. Praziquantel (Sigma-Aldrich) was used as the internal standard.

### 2.4 Data Processing

The raw data was acquired from GCMS Solution 4.50 SP1 software (Shimadzu Co., Japan) before being converted to “mzML” format with ProteoWizard software.^22^ All raw data files were deconvoluted using MSHub on the GNPS web-based platform.^20^ The deconvolution of the GC-MS data set was performed using MSHub. Subsequently, all features were organized into a molecular network and spectra matching against GNPS database using the following parameters: fragment ion mass tolerance was set to 0.5 Da, with a minimum of 6 matched peaks required for a spectral pair to be connected. A cosine score threshold of 0.5 was applied to define spectral similarity. The resulting molecular network was exported and further visualized using Cytoscape (version 3.10.1) for structural clustering and annotation of metabolites.

### 2.5 Statistical Analysis

The downstream processing of nontargeted metabolomics was performed following the approach outlined by Pakkir Shah.^23^ All detected features were weighted by using an internal standard. Data cleanup was then carried out, which included blank removal, imputation, normalization (total ion current), and scaling using the autoscaling technique. Statistical and machine learning analyses of the sugarcane waste data set were performed using R version 4.1.3.

## 3 Results and Discussion

### 3.1 Sugarcane Biomass Extraction Yields across Solvent Variations

Sugarcane parts and processing waste including tops, stems, trash (a mix of green tops and dry leaves left after harvesting), bagasse, and filter cake (see Table 1) were subjected to Soxhlet apparatus using five organic solvents of increasing polarity. These solvents included several low-cost and relatively environmentally friendly options. As illustrated in Figure 1, filter cake consistently yielded the highest total extraction across all five solvents, while bagasse produced the lowest. Among the materials extracted with the same solvent, sugarcane trash achieved the highest yield in methanol (12.4%). In contrast, filter cake resulted in the highest yields (5.9–11.0%) with the other solvents. Overall, methanol demonstrated the highest extraction efficiency, followed in order by ethanol, ethyl acetate, *tert*-butyl methyl ether, and hexane.

**Figure 1:**
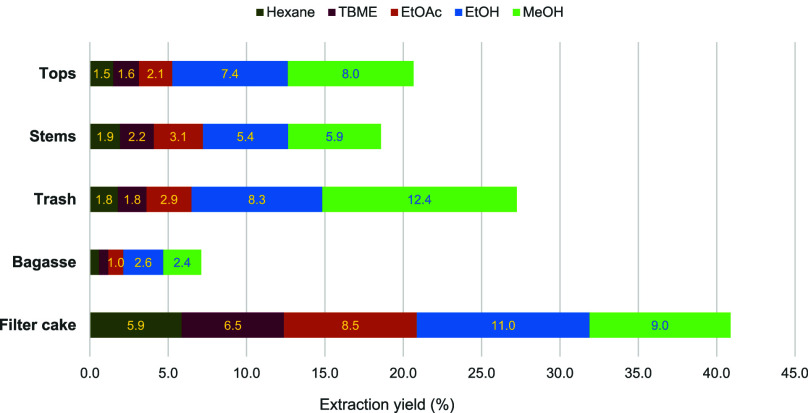
Percentage yields of extracts obtained from Soxhlet extraction of five sugarcane byproducts using five solvents with a broad range of polarities.

Beyond extraction efficacy, solvent choice should also be considered from a biorefinery and industrial feasibility perspective. Ethanol represents the most balanced and sustainable option because of its relatively low cost, good extraction efficacy, biodegradability, renewability, favorable safety profile, and ease of recovery and recycling in industrial processes. Ethyl acetate is also an acceptable alternative because of its moderate efficiency for semipolar compounds, relatively low toxicity, and good recoverability by distillation. Methanol provides high extraction efficiency at low cost; however, its toxicity and volatility raise safety concerns and require strict handling procedures. Similarly, *tert*-butyl methyl ether (TBME) is effective for extracting nonpolar to moderately polar compounds, although environmental considerations may limit its sustainability. In contrast, hexane is the least favorable option due to its high volatility and environmental impact, despite its strong extraction ability. Therefore, greener and safer solvents should be prioritized for industrial scale applications.^24,25^

### 3.2 Cluster Analysis of Compound Classes via GC-MS-Based Molecular Networking

Each of the 25 extracts, obtained through Soxhlet extraction of five sugarcane materials (tops, stems, trash, bagasse, and filter cake) using five different solvents (EtOH, MeOH, EtOAc, TBME, and hexane), was derivatized with BSTFA. The resulting samples were analyzed using GC-MS (single quadrupole), and the raw data were processed and uploaded to the GNPS platform. A molecular network was generated via GNPS and visualized using Cytoscape (Figure 2). In this network, each mass ion (spectrum) is represented as a node and annotated through dereplication using public mass spectral libraries available in the GNPS community repository. Nodes are interconnected by edges based on spectral similarity, forming clusters of related metabolites. The size of each node reflects the total chromatographic peak area summed across all 25 extracts, while the pie charts overlaid on each node indicate the relative abundance of that metabolite across the 5 solvent systems for each sugarcane material.

**Figure 2:**
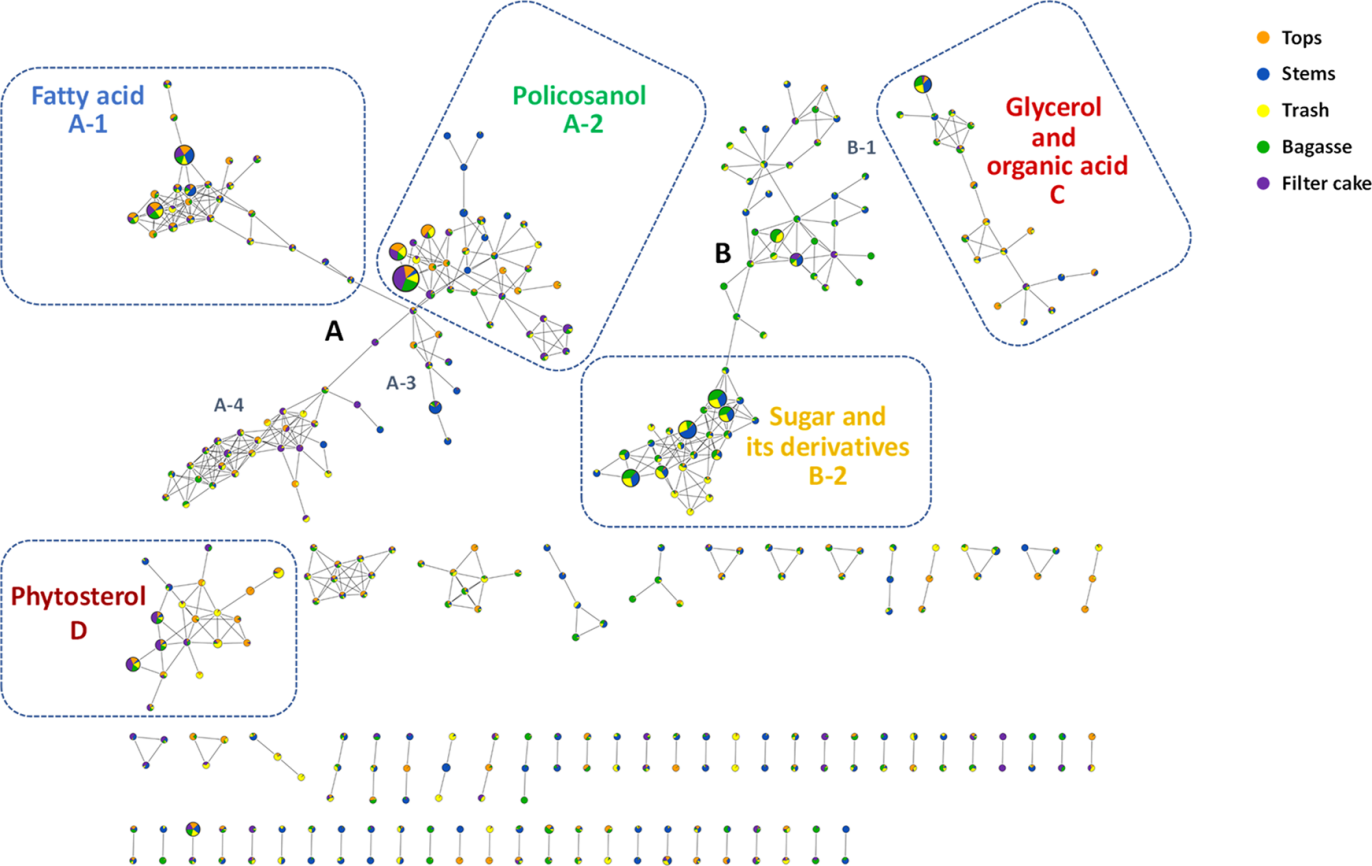
Molecular networking of sugarcane waste extracts revealed four major compound clusters: (A) fatty acids, policosanols, hydrocarbon, and related compounds; (B) sugars and their derivatives; (C) glycerol, polyols, and organic acids; and (D) phytosterols and triterpenoids.

A total of 357 mass spectral features were detected, of which approximately 33% were annotated (listed in Table S1). Annotation was achieved through a combination of tentative GNPS identifications, comparison of retention times and MS fragmentation patterns with authentic standard compounds, and spectral matching with the NIST 17 library. The resulting molecular network comprised four major clusters (Figure 2), with putative compounds and their associated clusters listed in Table S2. Cluster A, the largest, was further divided into four subclusters: (A-1) saturated fatty acids; (A-2) policosanols, fatty acid ester, and diterpene alcohol; (A-3) fatty acid; and (A-4) hydrocarbons (Figure 3). Cluster B primarily contained sugars and their derivatives, while cluster C included glycerol, polyols, and organic acids. Phytosterol and triterpenoids were classified in cluster D (Figure 4).

**Figure 3:**
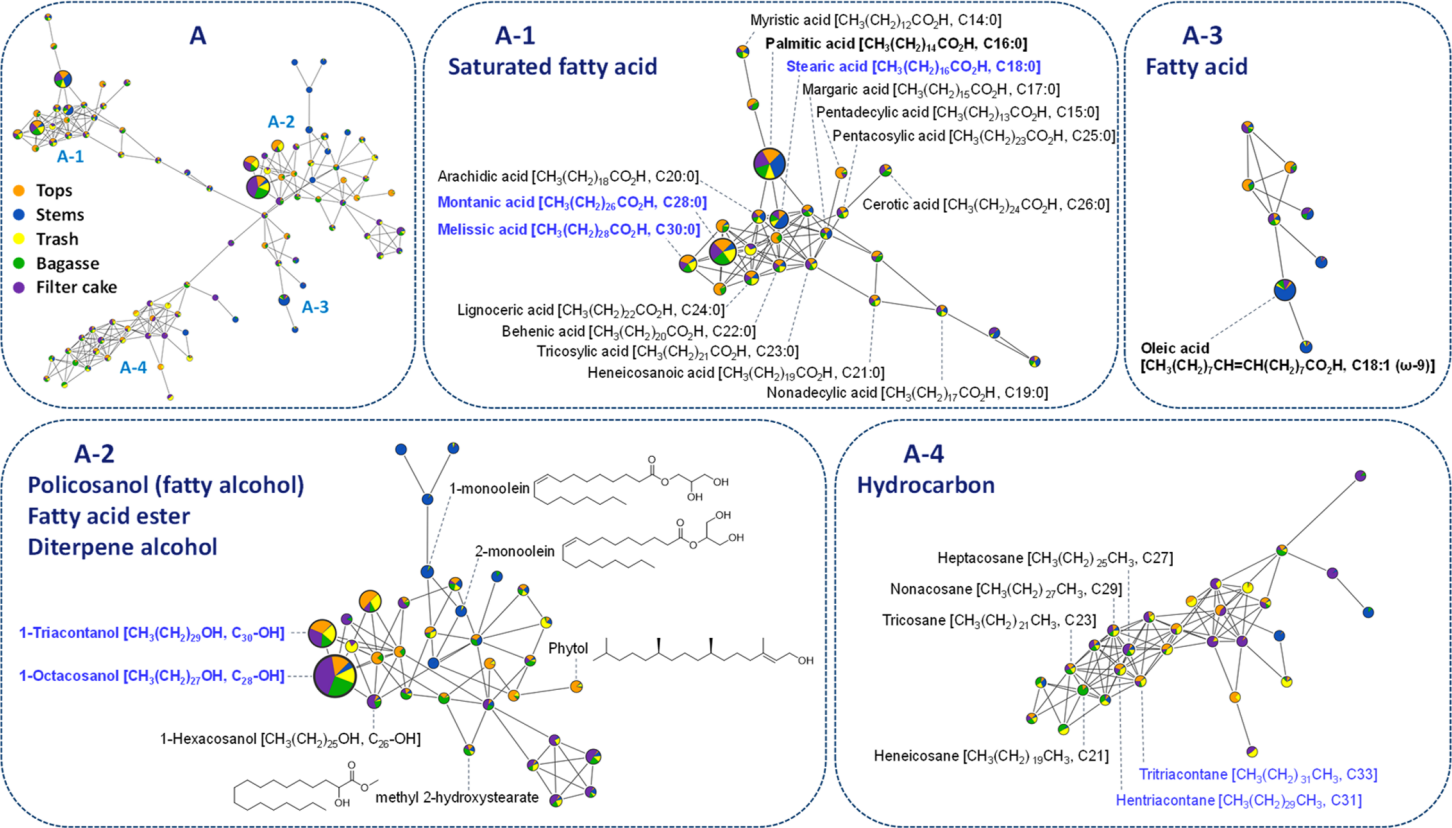
Molecular networking and metabolite annotation within cluster A. Metabolites labeled with blue text were identified by comparing both retention times and mass spectral patterns with authentic standard compounds.

**Figure 4:**
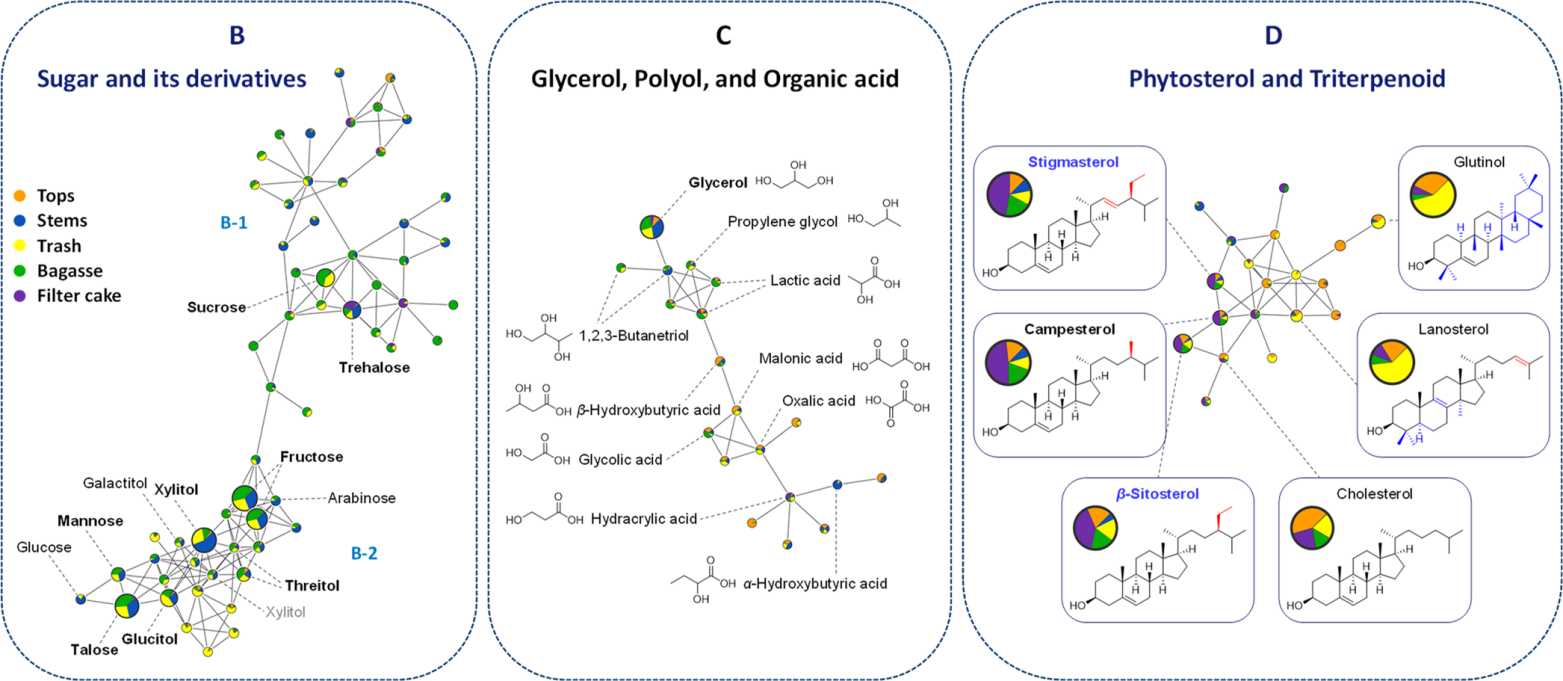
Molecular networking and metabolite annotation in Clusters B–D. Metabolites labeled in blue were identified by comparing both retention times and mass spectral patterns with authentic standard compounds.

As shown in Figures 2 and 3, octacosanol in subcluster A-2 exhibits the largest node size. The pie charts within the node depict its relative abundance in the following order: filter cake > bagasse > tops > trash > stems. Triacontanol and hexacosanol appear to be neighboring compounds. Triacontanol has a smaller node size compared to octacosanol, with its relative abundance ranked as follows: tops > filter cake > trash > bagasse > stems. Hexacosanol, with a significantly smaller node size than triacontanol, is most abundant in the filter cake, while the remaining 40% is distributed among the other four materials. In addition to policosanols, subcluster A-2 also includes fatty acid esters and diterpene alcohol.

Palmitic acid, ranking second in node size, was classified within subcluster A-1, which consists of saturated fatty acids ranging from C14:0 to C30:0, as shown in Figures 2 and 3. Palmitic acid (C16:0), margaric acid (C17:0), and stearic acid (C18:0) were present in high proportions in the stems. In contrast, heneicosanoic acid (C21:0), pentacosylic acid (C25:0), montanic acid (C28:0), and melissic acid (C30:0) were found in lower proportions in stems compared to other biomass components. For unsaturated fatty acids, oleic acid was identified in subcluster A-3. Subcluster A-4 contains smaller nodes representing hydrocarbons, including alkanes with odd numbers of carbons (C21, C23, C27, C29, C31, and C33) as well as a few alkenes, the names of which require confirmation.

Sugars and their derivatives were found in cluster B, which is divided into two subclusters. Subcluster B-1 comprises disaccharides, while monosaccharides and sugar alcohols were grouped in subcluster B-2. Most sugars and their derivatives were relatively abundant in the stems, trash, and bagasse. Cluster C encompasses a molecular network of glycerol and its derivatives including polyols and organic acids. Within this cluster, glycerol has the largest node, with its relative abundance following the order stems, followed by bagasse, trash, tops, and filter cake.

Phytosterols and triterpenoids were grouped into cluster D. Phytosterols, including β-sitosterol, stigmasterol, and campesterol, are main metabolites annotated by GNPS, with their node sizes decreasing accordingly. The relative abundance patterns of these compounds across the five sugarcane materials of these compounds shows a similar trend, with the highest proportion found in filter cake and the lowest in stems. Glutinol and lanosterol, identified as triterpenoids using the GNPS and NIST 17 library matching, were observed with smaller node sizes but high relative abundance in trash (∼60%) and tops (∼20–30%). These compounds were almost undetectable in stems.

Phenolic compounds annotated by GNPS—including protocatechuic acid and chlorogenic acid—and those identified through the NIST 17 library—such as vanillic acid, *p*-coumaric acid, and 5-methoxysalicylic acid—were detected with relatively small node sizes. Although these compounds were not grouped in the same molecular cluster, they appeared as tripletons and doubletons alongside other compound classes or unidentified metabolites. *p*-Coumaric acid, confirmed by comparison with the authentic standard, was detected at a relatively low-intensity molecular ion, with the highest abundance in bagasse (∼50%), followed by tops, stems, trash, and filter cake (∼1%).

The remaining ∼67% likely represent low-abundance metabolites, known metabolites not yet included in existing libraries or requiring confirmation with authentic standards, or structurally novel molecules. In future research, advanced computational MS tools could support structural prediction and facilitate the identification of novel metabolites. Moreover, integrating complementary analytical platforms, such as LC-MS/MS, may further expand metabolite coverage and enable the detection of previously uncharacterized compounds.

### 3.3 Comparative Phytochemical Analysis in Sugarcane Biomass

Unlike the molecular networking analysis in the previous section, which utilized raw GC-MS data without prior filtering, the statistical analyses in this section were conducted using a curated data set. The metabolite profile data were preprocessed by removing blank features, imputing missing values, and applying scaling prior to principal coordinate analysis (PCoA), as shown in Figure 5. These preprocessing steps improved the data robustness and enhanced the interpretability of the downstream statistical analyses.

**Figure 5:**
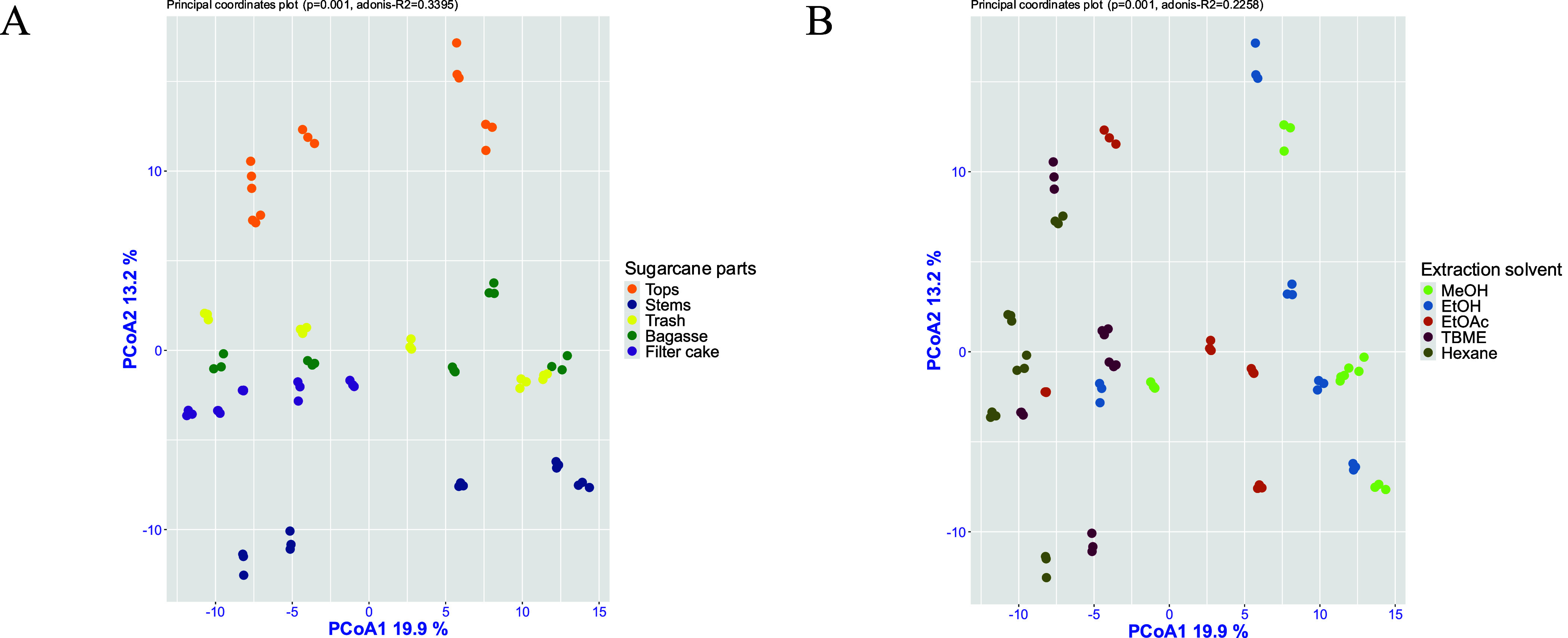
Principal coordinates analysis (PCoA) illustrating variation in metabolite profiles across (A) sugarcane byproducts (tops, stems, trash, bagasse, and filter cake) and (B) extraction solvents (EtOH, MeOH, EtOAc, TBME, and hexane).

PCoA was performed to evaluate the influence of sugarcane parts and extraction solvents on GC-MS metabolite profiles. The first two principal coordinates explained 19.9 and 13.2% of the total variance, respectively, indicating the major compositional gradients within the metabolomic data set. As shown in Figure 5A, extracts derived from the same sample type clustered together, forming three distinct groups, with tops and stems clearly separated from the other byproducts. PERMANOVA analysis (*R* ^2^ = 0.3395, *p* = 0.001) indicated that biomass type accounted for approximately 34% of the total variance, reflecting a strong influence of the anatomical origin on chemical composition.

In contrast, clustering based on extraction solvents showed greater overlap among groups (Figure 5B), although the separation remained statistically significant (*R* ^2^ = 0.2258, *p* = 0.001). These results reflect the solvent selectivity during extraction. Polar solvents such as ethanol and methanol preferentially extract sugars, organic acids, and phenolic compounds, whereas less-polar solvents such as TBME and hexane enrich for waxes, sterols, and long-chain hydrocarbons. Ethyl acetate, with intermediate polarity, extracts a broader range of semipolar compounds. Overall, these results indicate that the biological matrix exerts a stronger influence on metabolite composition than solvent choice, although solvent polarity modulates the detectable metabolome within each matrix.

To further interpret the PCoA ordination, metabolites significantly associated with the principal coordinates were examined. Long-chain fatty acids (e.g., pentacosanoic acid, nonadecanoic acid, and tricosanoic acid), sterols (e.g., cholesterol and stigmasterol), and long-chain hydrocarbons (e.g., nonacosane and heptacosane) showed strong correlations with the ordination axes. These compounds are characteristic components of plant cuticular waxes and structural lipids and likely contribute to the separation of sugarcane tissues in the ordination space. Detailed statistical associations between metabolites and the PCoA axes are provided in Tables S3 and S4. A PCoA biplot (Figure S1) further highlighted several wax-related metabolites, including pentacosanoic acid and tricosanoic acid, as key contributors to the sample differentiation.

Consistent with these results, hierarchical clustering heatmap analysis (Figure 7) revealed distinct metabolite enrichment patterns across the sugarcane biomass fractions. Several long-chain fatty acids, including tetradecanoic acid, heptadecanoic acid, nonadecanoic acid, tricosanoic acid, and octacosanoic acid, showed differential accumulation among the biomass types. In addition, phytosterols, such as stigmasterol and campesterol, were enriched in specific clusters. Together, these results indicate that metabolite composition differs significantly among sugarcane biomass fractions, with structural lipids and wax-related metabolites playing a major role in driving these differences.

To further validate the chemotypic differentiation among sugarcane byproducts, a random forest classification model was applied to the GC-MS metabolite data. The proximity matrix, visualized using multidimensional scaling (MDS), revealed five well-separated clusters corresponding to bagasse, filter cake, stems, tops, and trash (Figure 6A), confirming the distinct metabolic signatures of each biomass type. Model performance was evaluated using classification metrics (Figure 6B and Table 2). The random forest model achieved 100% accuracy, correctly classifying all 75 samples (15 per class). The out-of-bag (OOB) error was negligible, and model convergence was achieved with fewer than 50 trees. Class-wise accuracy for all biomass types was 100% (with 95% confidence interval of 78.2–100%), and the overall model accuracy also reached 100%, (with 95% confidence interval of 95.2–100%). No misclassifications were observed in the confusion matrix, indicating strong discriminatory power of the GC-MS metabolite features.

**Figure 6:**
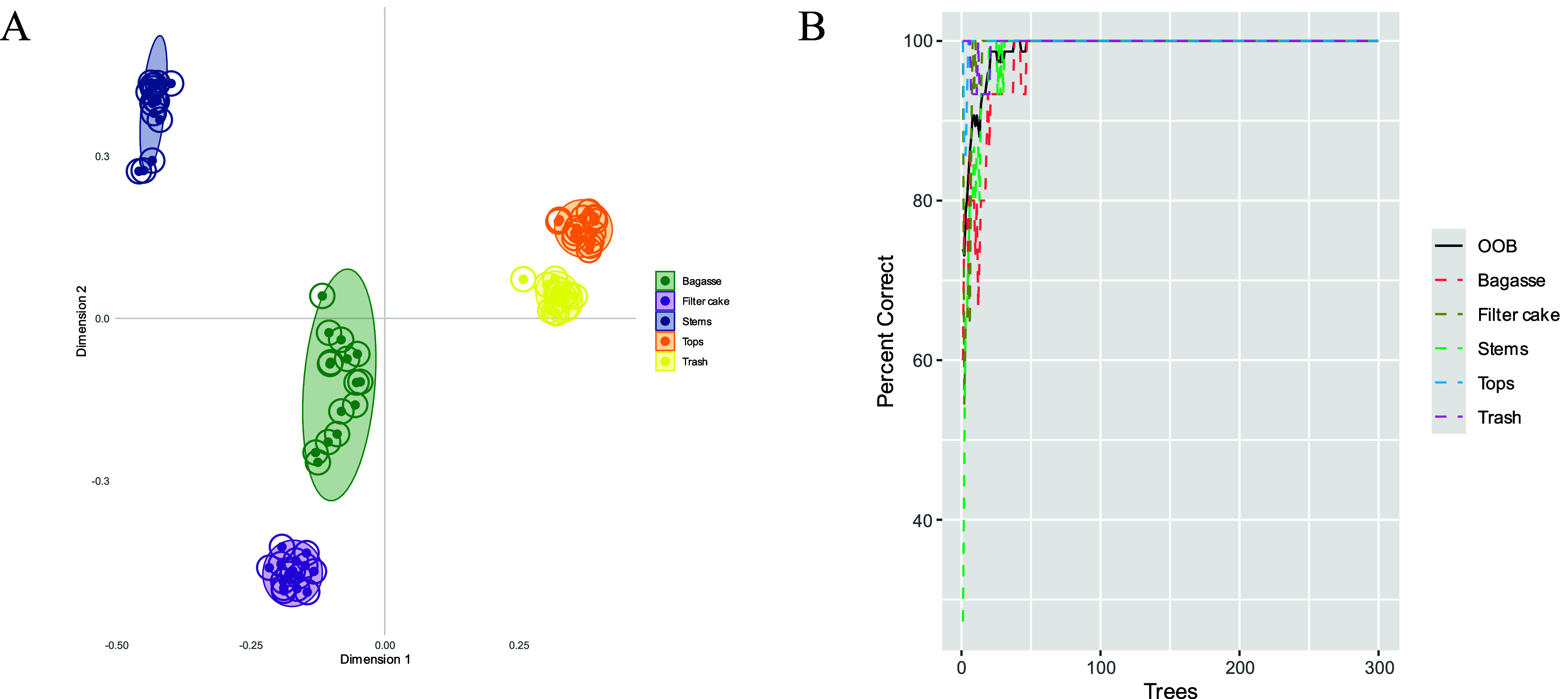
Classification of sugarcane byproducts based on GC-MS metabolite profiles using random forest. (A) Multidimensional scaling (MDS) plot derived from the random forest proximity matrix. (B) Classification accuracy across 300 trees with minimal out-of-bag (OOB) error.

**Table 2:** Confusion Matrix and Classification Accuracy of the Random Forest Model for Sugarcane Byproducts Based on GC-MS Metabolite Profiles^a^

	bagasse	filter cake	stems	tops	trash	pct.correct	LCI_0.95	UCI_0.95
bagasse	15	0	0	0	0	100	78.2	100
filter cake	0	15	0	0	0	100	78.2	100
stems	0	0	15	0	0	100	78.2	100
tops	0	0	0	15	0	100	78.2	100
trash	0	0	0	0	15	100	78.2	100
overall	NA	NA	NA	NA	NA	100	95.2	100

a NA: not available; pct correct: percent correct; LCI: lower confidence interval; UCI: upper confidence interval.

To further evaluate model robustness, repeated 10-fold cross-validation was performed. The random forest model consistently achieved a mean classification accuracy of 1.000 ± 0.000 across validation folds (Table S5). The pooled confusion matrix confirmed that all samples were correctly classified across validation runs (Table S6). In contrast, permutation testing yielded substantially lower accuracy (0.197 ± 0.068), close to the random expectation for five classes (∼0.20), confirming that the observed classification performance is not due to chance (*p* < 0.001). The comparison between cross-validation accuracy and permutation-based accuracy is shown in Figure S2. Although the random forest model demonstrated perfect classification performance under cross-validation, the number of samples per class remains relatively limited. Future studies incorporating independent data sets from different harvest seasons, sugarcane cultivars, or geographic regions would be valuable to further assess model generalizability.

Given the confirmed robustness of the classification model, feature importance analysis (Table S7) was used to identify metabolites contributing to the sample discrimination. The top-ranked metabolites included tetratriacontanol, hexadecanoic acid, (9*Z*)-octadecenoic (oleic acid) acid, glutinol, and *n*-hexacosanol. These compounds have been reported to exhibit bioactivities relevant to cosmetic applications, including antioxidant, emollient, and anti-inflammatory properties.^16,26^ Their enrichment in specific sugarcane byproducts highlights the potential for targeted extraction of high-value metabolites from sugarcane waste streams within sustainable biorefinery systems.

### 3.4 Comparative Analysis of Metabolites in Sugarcane Biomass

Following PCoA, metabolites with high molecular ion intensities were analyzed to identify the key contributors to sample differentiation. A heatmap (Figure 7) of the top 50 annotated metabolites was constructed from autoscaled chromatographic intensities to visualize the distribution and relative abundance of annotated compounds, enabling direct comparison of metabolite profiles across biomass types and extraction conditions. Hierarchical clustering identified four distinct metabolite groups. Cluster 1 primarily comprised polar compounds, including sugars (e.g., fructose, talose, mannose), sugar alcohols (e.g., xylitol, threitol, glucitol), sugar acids (gluconic acid), organic acids (e.g., citric acid, aconitic acid), and glycerol. Extraction with highly polar solvents (EtOH, MeOH, and EtOAc) resulted in elevated levels of these primary metabolites in stems, bagasse, and trash, reflecting the physiological role of these tissues in supporting growth and development. Cluster 2 comprised long-chain fatty acids, organic acids, a monoglyceride, and a phenolic acid. Tetradecanoic acid, *p*-coumaric acid, ribonic acid, and other organic acids (glycolic acid, glyceric acid, and lactic acid) were most abundant in the tops when extracted with highly polar solvents (MeOH and EtOH). In contrast, long-chain saturated fatty acids (C16:0, C17:0, C18:0, and C20:0), the unsaturated fatty acid (oleic acid, C18:1), and 1-oleoylglycerol exhibited more variable distributions in stems extracted with low-polarity solvents (hexane and TBME). These patterns indicate tissue-specific metabolic allocation: tops are enriched in phenolic and short-chain organic acids for metabolic activity, whereas stems are enriched in structural lipids that contribute to membrane integrity, cuticular wax formation, and overall protection. Cluster 3 included fatty acids (C23:0 and C26:0), alkanes (C27 and C29), plant sterols and triterpenoids (stigmasterol, β-sitosterol, campesterol, and stigmastanol), and policosanols (C26 and C28). These metabolites were predominantly found in filter cake extracts obtained with hexane and TBME, as well as in bagasse and tops extracted with hexane, consistent with their lipophilic nature and surface localization in sugarcane biomass. Lastly, cluster 4 contained very long-chain saturated fatty acids (e.g., C22:0, C24:0, C28:0, C30:0), alkanes (C31), and alcohols (policosanols with C30, C32, and C34), along with the diterpene alcohol (phytol), and triterpenoids (glutinol and lanosterol). These hydrophobic compounds were highly concentrated in tops and trash samples and were best recovered using nonpolar solvents (TBME and hexane). Their accumulation in leaf and residue surfaces is consistent with structural and protective roles, particularly in forming waxy barriers that reduce water loss and protect against environmental stress.

**Figure 7:**
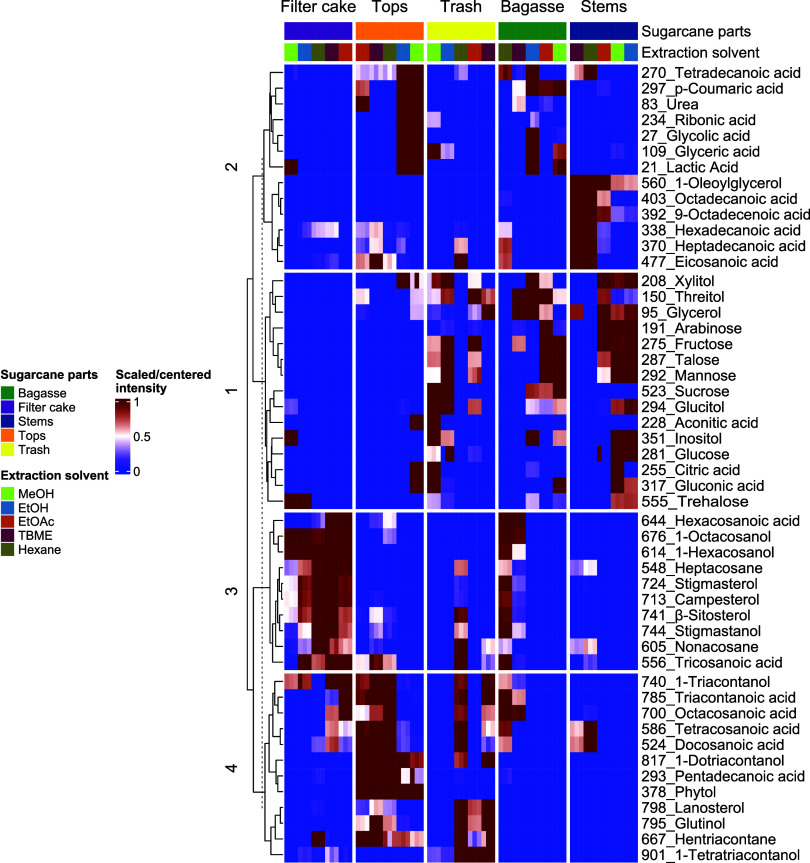
Heatmap showing relative metabolite abundances in sugarcane materials extracted with solvents of different polarity (columns). Extracts were analyzed in triplicate (*n* = 3). Colors range from blue (low abundance) through white (medium abundance) to dark red (high abundance), representing chromatographic peak areas. Labels indicate the MZmine cluster index and putatively identified metabolites (listed in Table S8).

Given their prominent molecular ion intensities and potential applications in nutraceutical and cosmetic industries, policosanols, phytosterols, and triterpenoids were analyzed in detail. Filter cake was the principal source of policosanols, particularly *n*-hexacosanol and *n*-octacosanol, with the highest levels in TBME extracts, followed by EtOAc and hexane. Bagasse served as a secondary source, especially in hexane and TBME extracts. Other policosanols showed varying tissue specificity: *n*-triacontanol occurred in filter cake, tops, and trash, *n*-dotriacontanol was enriched in tops, and *n*-tetratriacontanol was mainly found in trash extracts. Phytosterols (β-sitosterol, stigmasterol, campesterol, stigmastanol) displayed similar enrichment patterns, peaking in filter cake extracts obtained with hexane, followed by TBME and EtOAc, with moderate levels in trash and bagasse. Triterpenoids (lanosterol and glutinol) were primarily concentrated in trash extracts (hexane, TBME, and EtOAc), with lower levels in tops. These distributions suggest sterol accumulation in lipid-rich inner tissues.

Extraction yields (Figure 1) were incorporated to assess the practical applicability. Methanol extraction of trash provided the highest overall recovery of target metabolites, followed by ethanol extraction of the filter cake, highlighting the effectiveness of food-grade solvents. Low-polarity solvents (hexane, TBME, and EtOAc) produced lower absolute abundances but higher selectivity, defined as the proportion of target metabolites relative to total extracted compounds. Polar solvents yielded higher total quantities but coextracted more nontarget metabolites, reducing relative selectivity.

These findings have direct implications for sugarcane biorefinery applications. The composition of the biomass determines the abundance of high-value metabolites, while solvent polarity governs extraction efficiency and selectivity. For selective recovery, phytosterols are most effectively extracted with hexane from filter cake, policosanols with TBME from filter cake, and triterpenoids with hexane from trash. For maximal overall recovery, methanol extraction of trash or ethanol extraction of filter cake provides the highest yield. Applying a dual-extraction strategy, aligned with solvent polarity and biomass characteristics, can further improve selectivity, efficiency, and profitability, supporting a more targeted and sustainable sugarcane biorefinery approach.

## 4 Conclusions

This study presents a comprehensive GC-MS-based metabolomic profiling of five sugarcane byproducts: bagasse, filter cake, stems, tops, and trash. These materials were extracted using solvents with different polarities, including methanol, ethanol, ethyl acetate, *tert*-butyl methyl ether, and hexane, followed by derivatization and analysis. Metabolite annotation was conducted through molecular networking using the GNPS platform and was further validated by NIST libraries and reference standards. Despite inherent limitations of single-quadrupole mass spectrometry and database coverage, the integration of molecular networking and dereplication significantly enhanced compound identification. Multivariate statistical analysis, including principal coordinate analysis and random forest classification, revealed clear chemotypic differences among the sugarcane waste types. Both the biomass source and solvent polarity played critical roles in determining the abundance and selectivity of the metabolite extraction. Lipidic compounds such as policosanols, phytosterols, and triterpenoids were particularly enriched in the filter cake and trash. Nonpolar solvents were more selective for these compounds, while polar solvents extracted a broader range of metabolites with higher total yields. Random forest classification achieved 100% accuracy in distinguishing between sugarcane waste types, supporting the robustness of the approach. This study demonstrates that combining GC-MS-based molecular networking with supervised machine learning is an effective strategy for characterizing the chemical diversity in agricultural waste. The findings provide new insights into the targeted valorization of sugarcane residues and offer a practical framework for integrating biorefinery principles into sustainable industrial applications, particularly in the cosmetic, nutraceutical, and agricultural sectors.

## Supplementary Material



## Data Availability

All data generated or analyzed during this study are included in this article. All sample data sets are available on MassIVE at the University of California, San Diego Center for Computational Mass Spectrometry Web site (https://massive.ucsd.edu) with the following ID: MSV000091601. The deconvolution of the GC-MS data set using MSHub can be accessed at https://gnps.ucsd.edu/ProteoSAFe/status.jsp?task=7b9f7aed80cc420fa83179fde03c419e. The molecular network and spectra matching against the GNPS database are available at https://gnps.ucsd.edu/ProteoSAFe/status.jsp?task=3c38a100addc4da584c8ce60dddb0a00.

## Notes

Putative compounds identified in sugarcane byproducts (Table S1); putative compounds and their associated clusters in the molecular network presented in Figures 3 and 4 (Table S2); metabolites significantly associated with PCoA axes based on envfit analysis of the GC-MS metabolomics data set (Table S3); Spearman correlations between metabolite intensities and PCoA axes (Table S4); classification accuracy and balanced accuracy for each fold during repeated 10-fold cross-validation of the random forest model (Table S5); pooled confusion matrix summarizing random forest classification results across all repeated cross-validation runs (Table S6); metabolite importance scores based on random forest classification (Table S7); putative metabolites in the heatmap of Figure 7 with corresponding cluster indices (Table S8); PCoA biplot showing metabolites significantly associated with the ordination axes based on envfit analysis (Figure S1); and comparison of random forest classification accuracy from repeated cross-validation and permutation testing (Figure S2) (PDF)

## ∥

Department of Chemistry, Faculty of Science, Mahidol University, Bangkok 10400, Thailand

## ⊥

Plant Hormone Biology Group, Green Life Sciences Cluster, Swammerdam Institute for Life Science, University of Amsterdam, Science Park 904, Amsterdam 1098 XH, The Netherlands.

## #

Integrated Biorefinery Excellence Center (IBC), School of Energy and Environment, University of Phayao, Phayao 56000, Thailand.
