# Supplementary material for: Integrating Untargeted GC-MS Metabolomics, GNPS Molecular Networking, and Machine Learning for Sugarcane Byproduct Valorization
Source: ACS Omega. 2026 Apr 6;11(15):23241–51. doi: 10.1021/acsomega.6c00047 (PMC13103850; doi:10.1021/acsomega.6c00047)
Supplement: Supplementary file 1 [file ao6c00047_si_001.pdf]

# *Supporting Information*

## Integrating Untargeted GC-MS Metabolomics, GNPS Molecular Networking, and Machine Learning for Sugarcane By-Products Valorization

Thapanee Pruksatrakul [a], Chanakarn Sangsum [a, †], Sasina Makmai [a], Pornkanok Pongpamorn [a, ‡], Atchara Paemanee [a], Surachet Soontontaweesub [a], Seangaroon Yoiprommarat [a], Nantirat Sittichok [a, b], Nopparat Suriyachai [a, §], Suchat Pongchaiphol [a, b], Marisa Raita [a], Walaiporn Rungjang [c], Taridaporn Bunyapaiboonsri [a, *], and Verawat Champreda [a]

[a] *National Center for Genetic Engineering and Biotechnology (BIOTEC), National Science and Technology Development Agency (NSTDA), Pathum Thani 12120, Thailand*

[b] *The Joint Graduate School for Energy and Environment (JGSEE), King Mongkut's University of Technology Thonburi, Bangkok 10140, Thailand*

[c] *Innovation and Research Development Institute, Mitr Phol Sugarcane Research Center Co., Ltd., Phu Khiao, Chaiyaphum, Thailand*

[†] *Present address: Department of Chemistry, Faculty of Science, Mahidol University, Bangkok 10400, Thailand*

[‡] *Present address: Plant Hormone Biology Group, Green Life Sciences Cluster, Swammerdam Institute for Life Science, University of Amsterdam, Science Park 904, Amsterdam 1098 XH, The Netherlands*

[§] *Present address: Integrated Biorefinery Excellence Center (IBC), School of Energy and Environment, University of Phayao, Phayao 56000, Thailand*

* Corresponding author.

*E-mail address:* taridaporn@biotec.or.th (T. Bunyapaiboonsri)

## Content


| | | Pages |
|---|---|---|
| **Table S1.** | Putative Compounds Identified in Sugarcane Byproducts | *S3–S5* |
| **Table S2.** | Putative Compounds and Their Associated Clusters in the Molecular Network Presented in Figures 3 and 4 | *S6–S7* |
| **Table S3.** | Metabolites Significantly Associated with PCoA Axes Based on Envfit Analysis of the GC-MS Metabolomics Dataset | *S8–S16* |
| **Table S4.** | Spearman Correlations between Metabolite Intensities and PCoA Axes | *S17–S26* |
| **Table S5.** | Classification Accuracy and Balanced Accuracy for Each Fold during Repeated 10-Fold Cross-validation of the Random Forest Model | *S27–S32* |
| **Table S6.** | Pooled Confusion Matrix Summarizing Random Forest Classification Results across all Repeated Cross-validation Runs | *S33* |
| **Table S7.** | Metabolite Importance Scores Based on Random Forest Classification | *S34–S41* |
| **Table S8.** | Putative Metabolites in the Heatmap of Figure 7 with Corresponding Cluster Indices | *S42–S43* |
| **Figure S1.** | PCoA biplot showing metabolites significantly associated with the ordination axes based on envfit analysis | *S44* |
| **Figure S2.** | Comparison of random forest classification accuracy from repeated cross-validation and permutation testing | *S45* |

**Table S1. Putative Compounds Identified in Sugarcane Byproducts**

| No. | Cluster Index | Retention Time (min) | Putative Compound Name |
|---|---|---|---|
| 1 | 5 | 6.2 | Propane-1,2-diol (Propylene glycol) |
| 2 | 14 | 6.63 | 2-Hydroxypropanoic acid (Lactic acid) |
| 3 | 16 | 6.76 | Butane-2,3-diol |
| 4 | 21 | 6.99 | 2-Hydroxypropanoic acid (Lactic Acid) |
| 5 | 27 | 7.2 | Hydroxyacetic acid (Glycolic acid) |
| 6 | 31 | 7.34 | 2-Oxopropanoic acid (Pyruvic acid) |
| 7 | 49 | 7.88 | 1,2-Ethanedioic acid (Oxalic acid) |
| 8 | 50 | 7.88 | 1,2-Ethanedioic acid (Oxalic acid) |
| 9 | 55 | 8.04 | 3-Hydroxypropanoic acid (Hydracrylic acid) |
| 10 | 58 | 8.19 | 3-Hydroxybutanoic acid ($\beta$-Hydroxybutyric acid,) |
| 11 | 70 | 8.67 | Propanedioic acid (Malonic acid) |
| 12 | 73 | 8.74 | 3-Hydroxyisovaleric acid |
| 13 | 83 | 9.05 | Urea |
| 14 | 95 | 9.37 | Propane-1,2,3-triol (Glycerol) |
| 15 | 103 | 9.55 | 1,2,3-Butanetriol |
| 16 | 108 | 9.72 | 2,3-Diaminopropionic acid (3-Amino-alanine) |
| 17 | 109 | 9.91 | 2,3-Dihydroxypropanoic acid (Glyceric acid) |
| 18 | 111 | 10.13 | 2-Butenedioic acid (Fumaric acid) |
| 19 | 113 | 10.17 | 1,2,3-Butanetriol |
| 20 | 120 | 10.44 | Threonine |
| 21 | 122 | 10.52 | 2-Hydroxybutanoic acid ($\alpha$-hydroxybutyric acid) |
| 22 | 131 | 10.85 | 3,4-Dihydroxybutanoic acid |
| 23 | 144 | 11.23 | Ascorbic acid |
| 24 | 149 | 11.42 | Threitol |
| 25 | 150 | 11.49 | Threitol |
| 26 | 155 | 11.69 | Pyroglutamic acid |
| 27 | 156 | 11.76 | $\gamma$-Aminobutanoic acid (GABA) |
| 28 | 158 | 11.81 | Threonic acid |
| 29 | 173 | 12.35 | 2-Deoxyarabitol |
| 30 | 191 | 12.85 | Arabinose |
| 31 | 208 | 13.34 | Xylitol |
| 32 | 217 | 13.57 | Arabitol |
| 33 | 228 | 13.79 | Aconitic acid |
| 34 | 230 | 13.83 | 3,6-Anhydrogalactose |
| 35 | 234 | 13.92 | Ribonic acid |
| 36 | 242 | 14.2 | Vanillic acid |
| 37 | 247 | 14.28 | *p*-Coumaric acid |
| 38 | 255 | 14.5 | Citric acid |
| 39 | 257 | 14.56 | 3,4-Dihydroxybenzoic acid (Protocatechuic acid) |

| No. | Cluster Index | Retention Time (min) | Putative Compound Name |
|---|---|---|---|
| 40 | 270 | 14.88 | Tetradecanoic acid (Myristic acid) |
| 41 | 271 | 14.88 | Adonitol |
| 42 | 275 | 15.04 | Fructose |
| 43 | 281 | 15.22 | Glucose |
| 44 | 287 | 15.31 | Talose |
| 45 | 292 | 15.52 | Mannose |
| 46 | 293 | 15.62 | Pentadecanoic acid (Pentadecylic acid) |
| 47 | 294 | 15.66 | Glucitol |
| 48 | 297 | 15.87 | *p*-Coumaric acid |
| 49 | 305 | 16.07 | Galactose |
| 50 | 317 | 16.41 | Gluconic acid |
| 51 | 318 | 16.48 | 4-Hydroxy-3-methoxymandelic acid |
| 52 | 319 | 16.58 | Hexadecanoic acid (Palmitic acid) |
| 53 | 325 | 16.67 | 14-Methylhexadecanoic acid |
| 54 | 329 | 16.71 | *Myo*-Inositol |
| 55 | 338 | 16.97 | Hexadecanoic acid (Palmitic acid) |
| 56 | 351 | 17.4 | *Myo*-Inositol |
| 57 | 356 | 17.57 | *Myo*-Inositol |
| 58 | 361 | 17.63 | Heptadecanoic acid (Margaric acid) |
| 59 | 364 | 17.81 | Heneicosane |
| 60 | 365 | 17.86 | 2-Methylpentadecanoic acid |
| 61 | 368 | 17.97 | Mannitol |
| 62 | 370 | 18.01 | Heptadecanoic acid (Margaric acid) |
| 63 | 374 | 18.14 | Methyl 2-hydroxystearate |
| 64 | 378 | 18.31 | Phytol |
| 65 | 386 | 18.56 | Ethyl palmitate |
| 66 | 391 | 18.71 | (9*Z*)-Octadecenoic acid (Oleic acid) |
| 67 | 392 | 18.78 | (9*Z*)-Octadecenoic acid (Oleic acid) |
| 68 | 393 | 18.86 | (9*Z*)-Octadecenoic acid (Oleic acid) |
| 69 | 394 | 18.86 | (9*Z*)-Octadecenoic acid (Oleic acid) |
| 70 | 403 | 19.05 | Octadecanoic acid (Stearic acid) |
| 71 | 405 | 19.13 | galactinol |
| 72 | 429 | 19.68 | Tricosane |
| 73 | 440 | 20.05 | Nonadecanoic acid (Nonadecylic acid) |
| 74 | 477 | 21.04 | Eicosanoic acid (Arachidic acid) |
| 75 | 478 | 21.08 | Eicosanoic acid (Arachidic acid) |
| 76 | 503 | 21.99 | Heneicosanoic acid (Heneicosylic acid) |
| 77 | 523 | 22.82 | Sucrose |
| 78 | 524 | 22.92 | Docosanoic acid (Behenic acid) |
| 79 | 526 | 22.98 | Glucose-1-phosphate |
| 80 | 537 | 23.25 | Mannobiose |

| No. | Cluster Index | Retention Time (min) | Putative Compound Name |
|---|---|---|---|
| 81 | 540 | 23.3 | Sucrose |
| 82 | 548 | 23.52 | Heptacosane |
| 83 | 549 | 23.64 | 2-Oleoylglycerol (2-monoolein) |
| 84 | 555 | 23.79 | Trehalose |
| 85 | 556 | 23.82 | Tricosanoic acid (Tricosylic acid) |
| 86 | 560 | 23.95 | 1-Oleoylglycerol (1-monoolein) |
| 87 | 584 | 24.55 | (*Z*)-13-Docosenamide |
| 88 | 586 | 24.69 | Tetracosanoic acid (Lignoceric acid) |
| 89 | 593 | 24.93 | Melibiose |
| 90 | 605 | 25.26 | Nonacosane |
| 91 | 608 | 25.31 | Nonacosane |
| 92 | 613 | 25.54 | Pentacosanoic acid (Pentacosylic acid) |
| 93 | 614 | 25.6 | 1-Hexacosanol |
| 94 | 629 | 26 | Praziquantel |
| 95 | 644 | 26.37 | Hexacosanoic acid (Cerotic acid) |
| 96 | 648 | 26.48 | 1,2-Epoxyoctadecane |
| 97 | 667 | 27 | Hentriacontane |
| 98 | 668 | 27.04 | Chlorogenic acid |
| 99 | 676 | 27.38 | 1-Octacosanol |
| 100 | 679 | 27.51 | Cholesterol |
| 101 | 700 | 28.38 | Octacosanoic acid (Montanic acid) |
| 102 | 710 | 28.58 | Heptacosanal |
| 103 | 711 | 28.58 | 5-Methoxysalicylic acid |
| 104 | 713 | 28.68 | Campesterol |
| 105 | 724 | 28.98 | Stigmasterol |
| 106 | 729 | 29.24 | Tritriacontane |
| 107 | 740 | 29.71 | 1-Triacontanol |
| 108 | 741 | 29.78 | $\beta$-Sitosterol |
| 109 | 744 | 29.95 | Stigmastanol |
| 110 | 785 | 31.12 | Triacontanoic acid (Melissic acid) |
| 111 | 795 | 31.66 | Glutinol |
| 112 | 798 | 31.82 | Lanosterol |
| 113 | 815 | 32.54 | Hentriacontanoic acid (Hentriacontylic acid) |
| 114 | 817 | 32.61 | 1-Dotriacontanol |
| 115 | 900 | 35.88 | Tritriacontanoic acid (Psyllic acid) |
| 116 | 901 | 35.95 | 1-Tetratriacontanol |
| 117 | 903 | 35.98 | Isobutyl tetratriacontyl ether |
| 118 | 904 | 36.02 | Butyl octacosyl ether |
| 119 | 907 | 36.29 | Nonadecanenitrile |

**Table S2. Putative Compounds and Their Associated Clusters in the Molecular Network Presented in Figures 3 and 4**

| Putative Compound Name | Cluster |
|---|---|
| Tetradecanoic acid (Myristic acid) | A1 |
| Pentadecanoic acid (Pentadecylic acid) | A1 |
| Hexadecanoic acid (Palmitic acid) | A1 |
| Heptadecanoic acid (Margaric acid) | A1 |
| Octadecanoic acid (Stearic acid) | A1 |
| Nonadecanoic acid (Nonadecylic acid) | A1 |
| Eicosanoic acid (Arachidic acid) | A1 |
| Heneicosanoic acid (Heneicosylic acid) | A1 |
| Docosanoic acid (Behenic acid) | A1 |
| Tricosanoic acid (Tricosylic acid) | A1 |
| Tetracosanoic acid (Lignoceric acid) | A1 |
| Pentacosanoic acid (Pentacosylic acid) | A1 |
| Hexacosanoic acid (Cerotic acid) | A1 |
| Octacosanoic acid (Montanic acid) | A1 |
| Triacontanoic acid (Melissic acid) | A1 |
| Methyl 2-hydroxystearate | A2 |
| Phytol | A2 |
| 2-Oleoylglycerol (2-monoolein) | A2 |
| 1-Oleoylglycerol (1-monoolein) | A2 |
| 1-Hexacosanol | A2 |
| 1-Octacosanol | A2 |
| 1-Triacontanol | A2 |
| 2-Methylpentadecanoic acid | A3 |
| (9*Z*)-Octadecenoic acid (Oleic acid) | A3 |
| Heneicosane | A4 |
| Tricosane | A4 |
| Heptacosane | A4 |
| Nonacosane | A4 |
| Hentriacontane | A4 |
| Tritriacontane | A4 |
| Sucrose | B1 |
| Trehalose | B1 |
| Threitol | B2 |
| Galacitol | B2 |
| Arabinose | B2 |
| Xylitol | B2 |
| Fructose | B2 |
| Glucose | B2 |
| Talose | B2 |
| Mannose | B2 |
| Glucitol | B2 |

| Putative Compound Name | Cluster |
|---|---|
| Propane-1,2-diol (Propylene glycol) | C |
| 2-Hydroxypropanoic acid (Lactic Acid) | C |
| Hydroxyacetic acid (Glycolic acid) | C |
| 1,2-Ethanedioic acid (Oxalic acid) | C |
| 3-Hydroxypropanoic acid (Hydracrylic acid) | C |
| 3-Hydroxybutanoic acid ($\beta$-Hydroxybutyric acid,) | C |
| Propanedioic acid (Malonic acid) | C |
| Propane-1,2,3-triol (Glycerol) | C |
| 1,2,3-Butanetriol | C |
| 2-Hydroxybutanoic acid ($\alpha$-hydroxybutyric acid) | C |
| Cholesterol | D |
| Campesterol | D |
| Stigmasterol | D |
| $\beta$-Sitosterol | D |
| Glutinol | D |
| Lanosterol | D |

**Table S3. Metabolites Significantly Associated with PCoA Axes Based on Envfit Analysis of the GC-MS Metabolomics Dataset**

| PCoA Axis1 | PCoA Axis2 | Feature | $R^2$ | p-value |
|---|---|---|---|---|
| -0.53 | 0.74 | 679_Cholesterol | 0.82805 | 0.001 |
| -0.87 | 0.24 | 613_Pentacosanoic acid (Pentacosylic acid) | 0.81485 | 0.001 |
| -0.88 | 0.18 | 556_Tricosanoic acid (Tricosylic acid) | 0.80947 | 0.001 |
| -0.87 | -0.21 | 440_Nonadecanoic acid (Nonadecylic acid) | 0.79763 | 0.001 |
| -0.81 | 0.36 | 503_Heneicosanoic acid (Heneicosylic acid) | 0.79176 | 0.001 |
| -0.79 | 0.38 | 524_Docosanoic acid (Behenic acid) | 0.76516 | 0.001 |
| -0.28 | 0.83 | 817_1-Dotriacontanol | 0.76405 | 0.001 |
| -0.78 | 0.38 | 586_Tetracosanoic acid (Lignoceric acid) | 0.75420 | 0.001 |
| -0.38 | 0.76 | 729_Tritriacontane | 0.73079 | 0.001 |
| -0.65 | 0.55 | 740_1-Triacontanol | 0.72030 | 0.001 |
| 0.78 | -0.33 | 287_Talose | 0.71624 | 0.001 |
| -0.20 | 0.82 | 365_2-Methylpentadecanoic acid | 0.70680 | 0.001 |
| -0.12 | 0.83 | 378_Phytol | 0.70629 | 0.001 |
| 0.81 | 0.22 | 208_Xylitol | 0.70151 | 0.001 |
| -0.83 | -0.10 | 605_Nonacosane | 0.69404 | 0.001 |
| -0.82 | -0.10 | 492_NA | 0.68815 | 0.001 |
| 0.02 | 0.82 | 542_NA | 0.67979 | 0.001 |
| -0.49 | 0.66 | 694_NA | 0.67948 | 0.001 |
| -0.51 | 0.65 | 667_Hentriacontane | 0.67681 | 0.001 |
| -0.65 | -0.50 | 611_NA | 0.67650 | 0.001 |
| -0.09 | 0.82 | 283_NA | 0.67466 | 0.001 |
| -0.29 | 0.76 | 293_Pentadecanoic acid (Pentadecylic acid) | 0.66915 | 0.001 |
| 0.79 | -0.21 | 300_NA | 0.66456 | 0.001 |
| 0.80 | -0.14 | 294_Glucitol | 0.65857 | 0.001 |
| -0.74 | 0.33 | 700_Octacosanoic acid (Montanic acid) | 0.65835 | 0.001 |
| 0.71 | -0.40 | 215_NA | 0.65817 | 0.001 |
| -0.44 | -0.68 | 469_NA | 0.65518 | 0.001 |
| -0.24 | 0.77 | 792_NA | 0.65172 | 0.001 |
| 0.76 | -0.28 | 292_Mannose | 0.65068 | 0.001 |
| -0.81 | 0.02 | 437_NA | 0.64923 | 0.001 |
| 0.80 | 0.00 | 312_NA | 0.64644 | 0.001 |
| -0.69 | -0.40 | 671_NA | 0.63853 | 0.001 |
| -0.73 | -0.32 | 735_NA | 0.63599 | 0.001 |
| -0.22 | 0.76 | 420_NA | 0.63199 | 0.001 |
| 0.78 | 0.12 | 158_Threonic acid | 0.62908 | 0.001 |
| 0.23 | 0.76 | 318_4-Hydroxy-3-methoxymandelic acid | 0.62564 | 0.001 |

| PCoA Axis1 | PCoA Axis2 | Feature | $R^2$ | p-value |
|---|---|---|---|---|
| 0.71 | -0.35 | 275_Fructose | 0.62262 | 0.001 |
| 0.61 | 0.50 | 109_2,3-Dihydroxypropanoic acid (Glyceric acid) | 0.62042 | 0.001 |
| -0.23 | 0.75 | 319_Hexadecanoic acid (Palmitic acid) | 0.61775 | 0.001 |
| 0.45 | 0.64 | 608_Nonacosane | 0.61530 | 0.001 |
| 0.68 | 0.39 | 268_NA | 0.61273 | 0.001 |
| -0.13 | 0.77 | 759_NA | 0.61192 | 0.001 |
| 0.69 | -0.35 | 280_NA | 0.60567 | 0.001 |
| 0.74 | 0.24 | 165_NA | 0.60443 | 0.001 |
| -0.46 | -0.63 | 760_NA | 0.60323 | 0.001 |
| -0.77 | 0.12 | 741_*β*-Sitosterol | 0.60032 | 0.001 |
| 0.42 | 0.65 | 297_*p*-Coumaric acid | 0.59603 | 0.001 |
| -0.01 | 0.77 | 853_NA | 0.59340 | 0.001 |
| -0.70 | 0.30 | 429_Tricosane | 0.58825 | 0.001 |
| -0.70 | -0.32 | 548_Heptacosane | 0.58765 | 0.001 |
| -0.64 | 0.42 | 785_Triacontanoic acid (Melissic acid) | 0.58533 | 0.001 |
| 0.68 | -0.35 | 366_NA | 0.58150 | 0.001 |
| -0.44 | 0.62 | 630_NA | 0.58073 | 0.001 |
| 0.58 | 0.49 | 600_NA | 0.58048 | 0.001 |
| -0.75 | -0.13 | 701_NA | 0.57817 | 0.001 |
| 0.74 | -0.16 | 242_Vanillic acid | 0.57111 | 0.001 |
| -0.39 | 0.65 | 361_Heptadecanoic acid (Margaric acid) | 0.56483 | 0.001 |
| 0.16 | 0.73 | 83_Urea | 0.56361 | 0.001 |
| -0.16 | 0.73 | 468_NA | 0.56332 | 0.001 |
| -0.75 | -0.04 | 744_Stigmastanol | 0.56243 | 0.001 |
| -0.72 | -0.20 | 814_NA | 0.56148 | 0.001 |
| -0.14 | 0.74 | 269_NA | 0.56071 | 0.001 |
| -0.74 | -0.07 | 738_NA | 0.55845 | 0.001 |
| -0.10 | 0.74 | 566_NA | 0.55248 | 0.001 |
| 0.67 | -0.32 | 582_NA | 0.55081 | 0.001 |
| 0.74 | -0.06 | 441_NA | 0.54489 | 0.001 |
| -0.32 | 0.66 | 818_NA | 0.53430 | 0.001 |
| -0.27 | 0.68 | 753_NA | 0.53223 | 0.001 |
| 0.71 | -0.16 | 317_Gluconic acid | 0.53140 | 0.001 |
| 0.70 | -0.19 | 351_*Myo*-Inositol | 0.53023 | 0.001 |
| 0.69 | -0.24 | 551_NA | 0.52630 | 0.001 |
| 0.24 | 0.68 | 58_3-Hydroxybutanoic acid (*β*-Hydroxybutyric acid,) | 0.52575 | 0.001 |
| -0.72 | -0.02 | 676_1-Octacosanol | 0.52392 | 0.001 |
| -0.30 | 0.65 | 802_NA | 0.52100 | 0.001 |
| -0.70 | -0.18 | 724_Stigmasterol | 0.52079 | 0.001 |

| PCoA Axis1 | PCoA Axis2 | Feature | $R^2$ | p-value |
|---|---|---|---|---|
| 0.60 | 0.40 | 512_NA | 0.51776 | 0.001 |
| 0.71 | 0.08 | 295_NA | 0.51658 | 0.001 |
| -0.53 | -0.48 | 432_NA | 0.51235 | 0.001 |
| 0.53 | 0.48 | 144_Ascorbic acid | 0.51184 | 0.001 |
| -0.61 | -0.37 | 55_3-Hydroxypropanoic acid (Hydracrylic acid) | 0.50923 | 0.001 |
| -0.33 | 0.63 | 514_NA | 0.50603 | 0.001 |
| 0.67 | -0.25 | 528_NA | 0.50424 | 0.001 |
| 0.17 | 0.68 | 609_NA | 0.49659 | 0.001 |
| 0.70 | -0.08 | 256_NA | 0.49409 | 0.001 |
| 0.18 | 0.68 | 649_NA | 0.48902 | 0.001 |
| -0.69 | -0.09 | 477_Eicosanoic acid (Arachidic acid) | 0.48824 | 0.001 |
| -0.69 | -0.11 | 713_Campesterol | 0.48811 | 0.001 |
| -0.48 | 0.50 | 815_Hentriacontanoic acid (Hentriacontylic acid) | 0.48322 | 0.001 |
| -0.48 | -0.50 | 647_NA | 0.48102 | 0.001 |
| -0.23 | 0.65 | 521_NA | 0.48095 | 0.001 |
| 0.54 | -0.43 | 191_Arabinose | 0.48009 | 0.001 |
| 0.37 | 0.58 | 811_NA | 0.47897 | 0.001 |
| 0.17 | 0.67 | 199_NA | 0.47586 | 0.001 |
| 0.57 | -0.39 | 188_NA | 0.47555 | 0.001 |
| 0.67 | 0.16 | 150_Threitol | 0.47255 | 0.001 |
| 0.66 | -0.17 | 230_3,6-Anhydrogalactose | 0.47015 | 0.001 |
| 0.45 | 0.52 | 31_2-Oxopropanoic acid (Pyruvic acid) | 0.46993 | 0.001 |
| 0.62 | -0.28 | 489_NA | 0.46761 | 0.001 |
| 0.54 | -0.41 | 507_NA | 0.46053 | 0.001 |
| -0.32 | -0.59 | 483_NA | 0.45722 | 0.001 |
| 0.19 | 0.65 | 423_NA | 0.45581 | 0.001 |
| -0.26 | 0.62 | 923_NA | 0.45400 | 0.001 |
| -0.65 | -0.16 | 727_NA | 0.44892 | 0.001 |
| 0.32 | 0.59 | 313_NA | 0.44718 | 0.001 |
| -0.19 | 0.64 | 568_NA | 0.44620 | 0.001 |
| -0.23 | -0.62 | 764_NA | 0.44455 | 0.001 |
| 0.66 | -0.04 | 155_Pyroglutamic acid | 0.44111 | 0.001 |
| -0.21 | -0.63 | 461_NA | 0.44055 | 0.001 |
| -0.61 | -0.26 | 717_NA | 0.43735 | 0.001 |
| -0.57 | 0.34 | 578_NA | 0.43642 | 0.001 |
| -0.35 | -0.56 | 711_5-Methoxysalicylic acid | 0.43476 | 0.001 |
| 0.23 | 0.62 | 425_NA | 0.43376 | 0.001 |
| -0.23 | 0.61 | 903_Isobutyl tetratriacontyl ether | 0.43184 | 0.001 |
| -0.23 | -0.61 | 496_NA | 0.43169 | 0.001 |

| PCoA Axis1 | PCoA Axis2 | Feature | $R^2$ | p-value |
|---|---|---|---|---|
| 0.53 | 0.39 | 5_Propane-1,2-diol (Propylene glycol) | 0.43119 | 0.001 |
| -0.66 | 0.02 | 516_NA | 0.43087 | 0.001 |
| 0.66 | 0.04 | 120_Threonine | 0.43081 | 0.001 |
| 0.64 | -0.15 | 404_NA | 0.43074 | 0.001 |
| 0.21 | 0.62 | 220_NA | 0.42943 | 0.001 |
| 0.38 | 0.54 | 27_Hydroxyacetic acid (Glycolic acid) | 0.42881 | 0.001 |
| 0.56 | -0.34 | 281_Glucose | 0.42652 | 0.001 |
| 0.26 | 0.60 | 116_NA | 0.42626 | 0.001 |
| 0.62 | 0.19 | 149_Threitol | 0.42563 | 0.001 |
| -0.65 | 0.04 | 644_Hexacosanoic acid (Cerotic acid) | 0.42366 | 0.001 |
| -0.57 | -0.31 | 338_Hexadecanoic acid (Palmitic acid) | 0.42192 | 0.001 |
| -0.29 | -0.58 | 478_Eicosanoic acid (Arachidic acid) | 0.41892 | 0.001 |
| -0.20 | -0.61 | 451_NA | 0.41823 | 0.001 |
| -0.12 | -0.63 | 603_NA | 0.41473 | 0.001 |
| -0.16 | -0.62 | 392_(9*Z*)-Octadecenoic acid (Oleic acid) | 0.41147 | 0.001 |
| 0.60 | -0.22 | 108_2,3-Diaminopropionic acid (3-Amino-alanine) | 0.40905 | 0.001 |
| -0.06 | -0.64 | 549_2-Oleoylglycerol (2-monoolein) | 0.40878 | 0.001 |
| 0.57 | -0.28 | 564_NA | 0.40673 | 0.001 |
| -0.64 | 0.00 | 393_(9*Z*)-Octadecenoic acid (Oleic acid) | 0.40433 | 0.001 |
| 0.63 | -0.04 | 156_$\gamma$-Aminobutanoic acid (GABA) | 0.40344 | 0.001 |
| -0.02 | -0.63 | 560_1-Oleoylglycerol (1-monoolein) | 0.40107 | 0.001 |
| -0.01 | 0.63 | 584_(*Z*)-13-Docosenamide | 0.40101 | 0.001 |
| -0.13 | -0.62 | 945_NA | 0.40057 | 0.001 |
| -0.46 | 0.43 | 599_NA | 0.39964 | 0.001 |
| -0.32 | -0.55 | 325_14-Methylhexadecanoic acid | 0.39907 | 0.001 |
| -0.23 | 0.59 | 884_NA | 0.39814 | 0.001 |
| -0.52 | 0.35 | 362_NA | 0.39275 | 0.001 |
| 0.60 | -0.17 | 95_Propane-1,2,3-triol (Glycerol) | 0.39014 | 0.001 |
| -0.62 | 0.09 | 558_NA | 0.38882 | 0.001 |
| -0.58 | -0.22 | 614_1-Hexacosanol | 0.38615 | 0.001 |
| 0.50 | -0.36 | 502_NA | 0.38208 | 0.001 |
| 0.48 | 0.38 | 131_3,4-Dihydroxybutanoic acid | 0.38194 | 0.001 |
| -0.19 | -0.59 | 755_NA | 0.38062 | 0.001 |
| 0.58 | -0.20 | 535_NA | 0.37857 | 0.001 |
| -0.12 | -0.60 | 330_NA | 0.37810 | 0.001 |
| 0.52 | 0.33 | 796_NA | 0.37802 | 0.001 |
| 0.60 | -0.11 | 389_NA | 0.37110 | 0.001 |
| -0.32 | 0.52 | 850_NA | 0.36656 | 0.001 |
| -0.57 | 0.20 | 465_NA | 0.36498 | 0.001 |

| PCoA Axis1 | PCoA Axis2 | Feature | $R^2$ | p-value |
|---|---|---|---|---|
| 0.31 | 0.52 | 234_Ribonic acid | 0.36468 | 0.001 |
| 0.56 | -0.22 | 454_NA | 0.36281 | 0.001 |
| 0.25 | 0.55 | 90_NA | 0.36071 | 0.001 |
| 0.10 | -0.59 | 173_2-Deoxyarabitol | 0.35980 | 0.001 |
| 0.52 | -0.30 | 122_2-Hydroxybutanoic acid ($\alpha$-hydroxybutyric acid) | 0.35670 | 0.001 |
| -0.26 | -0.54 | 403_Octadecanoic acid (Stearic acid) | 0.35441 | 0.001 |
| 0.58 | 0.13 | 612_NA | 0.35247 | 0.001 |
| 0.58 | -0.12 | 194_NA | 0.34928 | 0.001 |
| -0.10 | -0.58 | 386_Ethyl palmitate | 0.34790 | 0.001 |
| 0.59 | -0.05 | 523_Sucrose | 0.34757 | 0.001 |
| 0.48 | 0.34 | 67_NA | 0.34636 | 0.001 |
| 0.49 | -0.33 | 103_1,2,3-Butanetriol | 0.34585 | 0.001 |
| 0.45 | -0.37 | 298_NA | 0.34302 | 0.001 |
| -0.13 | -0.57 | 376_NA | 0.34230 | 0.001 |
| 0.57 | -0.13 | 206_NA | 0.34044 | 0.001 |
| -0.23 | 0.53 | 411_NA | 0.33900 | 0.001 |
| 0.35 | -0.46 | 296_NA | 0.33634 | 0.001 |
| 0.36 | -0.45 | 398_NA | 0.33590 | 0.001 |
| 0.56 | -0.12 | 434_NA | 0.33373 | 0.001 |
| -0.17 | -0.55 | 394_(9*Z*)-Octadecenoic acid (Oleic acid) | 0.33281 | 0.001 |
| 0.42 | 0.39 | 16_Butane-2,3-diol | 0.32608 | 0.001 |
| 0.57 | -0.04 | 255_Citric acid | 0.32345 | 0.001 |
| 0.55 | -0.16 | 547_NA | 0.32334 | 0.001 |
| -0.17 | -0.54 | 562_NA | 0.32192 | 0.001 |
| -0.48 | -0.31 | 370_Heptadecanoic acid (Margaric acid) | 0.31935 | 0.001 |
| -0.12 | -0.55 | 379_NA | 0.31491 | 0.001 |
| 0.34 | 0.44 | 50_1,2-Ethanedioic acid (Oxalic acid) | 0.31323 | 0.001 |
| 0.44 | -0.34 | 229_NA | 0.30865 | 0.001 |
| -0.54 | -0.11 | 374_Methyl 2-hydroxystearate | 0.30596 | 0.001 |
| 0.43 | -0.35 | 118_NA | 0.30201 | 0.001 |
| 0.53 | 0.13 | 583_NA | 0.29986 | 0.001 |
| -0.33 | 0.43 | 795_Glutinol | 0.29632 | 0.001 |
| -0.19 | -0.51 | 334_NA | 0.29379 | 0.001 |
| -0.49 | -0.22 | 799_NA | 0.29303 | 0.001 |
| -0.49 | -0.24 | 824_NA | 0.29269 | 0.001 |
| 0.39 | -0.38 | 227_NA | 0.29117 | 0.001 |
| -0.13 | -0.52 | 695_NA | 0.28873 | 0.001 |
| 0.53 | -0.08 | 428_NA | 0.28587 | 0.001 |
| 0.47 | -0.25 | 196_NA | 0.28283 | 0.001 |

| PCoA Axis1 | PCoA Axis2 | Feature | $R^2$ | p-value |
|---|---|---|---|---|
| 0.45 | -0.27 | 593_Melibiose | 0.28041 | 0.001 |
| -0.48 | -0.22 | 849_NA | 0.27940 | 0.001 |
| 0.52 | -0.06 | 383_NA | 0.27882 | 0.001 |
| 0.15 | 0.50 | 924_NA | 0.27349 | 0.001 |
| -0.14 | -0.50 | 499_NA | 0.27283 | 0.001 |
| 0.52 | 0.05 | 111_2-Butenedioic acid (Fumaric acid) | 0.27276 | 0.001 |
| 0.12 | 0.50 | 337_NA | 0.26489 | 0.001 |
| -0.48 | -0.18 | 20_NA | 0.26428 | 0.001 |
| 0.14 | 0.49 | 904_Butyl octacosyl ether | 0.26393 | 0.001 |
| -0.51 | 0.07 | 876_NA | 0.26096 | 0.001 |
| 0.47 | -0.20 | 356_*Myo*-Inositol | 0.25906 | 0.001 |
| 0.50 | -0.09 | 758_NA | 0.25884 | 0.001 |
| 0.44 | 0.26 | 405_galactinol | 0.25804 | 0.001 |
| 0.15 | 0.48 | 470_NA | 0.25640 | 0.001 |
| 0.33 | 0.38 | 21_2-Hydroxypropanoic acid (Lactic Acid) | 0.25586 | 0.001 |
| -0.49 | 0.11 | 617_NA | 0.25329 | 0.001 |
| -0.45 | -0.22 | 899_NA | 0.25202 | 0.001 |
| 0.43 | 0.25 | 674_NA | 0.24621 | 0.001 |
| 0.12 | 0.48 | 879_NA | 0.24617 | 0.001 |
| 0.47 | -0.14 | 569_NA | 0.24481 | 0.001 |
| 0.15 | 0.47 | 498_NA | 0.24367 | 0.001 |
| -0.48 | -0.13 | 391_(9*Z*)-Octadecenoic acid (Oleic acid) | 0.24366 | 0.001 |
| -0.14 | 0.47 | 270_Tetradecanoic acid (Myristic acid) | 0.23904 | 0.001 |
| 0.29 | 0.39 | 439_NA | 0.23557 | 0.001 |
| -0.44 | -0.19 | 805_NA | 0.23202 | 0.001 |
| -0.37 | 0.30 | 798_Lanosterol | 0.22883 | 0.001 |
| -0.27 | -0.39 | 485_NA | 0.22882 | 0.001 |
| 0.21 | 0.43 | 710_Heptacosanal | 0.22807 | 0.001 |
| -0.43 | -0.20 | 618_NA | 0.22686 | 0.001 |
| 0.37 | -0.30 | 917_NA | 0.22399 | 0.001 |
| -0.06 | -0.47 | 882_NA | 0.22365 | 0.001 |
| 0.47 | 0.08 | 305_Galactose | 0.22283 | 0.001 |
| 0.13 | 0.45 | 73_3-Hydroxyisovaleric acid | 0.22251 | 0.001 |
| 0.21 | -0.42 | 271_Adonitol | 0.22238 | 0.001 |
| -0.08 | -0.46 | 793_NA | 0.22069 | 0.001 |
| 0.44 | -0.16 | 368_Mannitol | 0.21871 | 0.001 |
| -0.30 | -0.36 | 648_1,2-Epoxyoctadecane | 0.21759 | 0.001 |
| 0.46 | -0.07 | 804_NA | 0.21708 | 0.001 |
| 0.29 | 0.36 | 169_NA | 0.21499 | 0.001 |

| PCoA Axis1 | PCoA Axis2 | Feature | $R^2$ | p-value |
|---|---|---|---|---|
| -0.23 | 0.40 | 929_NA | 0.21391 | 0.001 |
| 0.45 | -0.12 | 565_NA | 0.21274 | 0.001 |
| -0.07 | -0.46 | 956_NA | 0.21248 | 0.001 |
| 0.16 | 0.43 | 119_NA | 0.21207 | 0.001 |
| -0.45 | -0.11 | 703_NA | 0.21102 | 0.001 |
| 0.46 | -0.06 | 598_NA | 0.21056 | 0.001 |
| 0.07 | 0.45 | 486_NA | 0.20883 | 0.001 |
| 0.42 | -0.18 | 552_NA | 0.20393 | 0.001 |
| 0.28 | 0.36 | 70_Propanedioic acid (Malonic acid) | 0.20229 | 0.001 |
| 0.36 | -0.26 | 689_NA | 0.19993 | 0.001 |
| -0.32 | -0.31 | 806_NA | 0.19926 | 0.001 |
| 0.22 | 0.39 | 431_NA | 0.19857 | 0.001 |
| 0.43 | -0.10 | 285_NA | 0.19520 | 0.001 |
| 0.42 | -0.14 | 488_NA | 0.19485 | 0.001 |
| 0.16 | -0.41 | 257_3,4-Dihydroxybenzoic acid (Protocatechuic acid) | 0.19327 | 0.001 |
| -0.37 | -0.23 | 739_NA | 0.18972 | 0.001 |
| -0.42 | 0.08 | 658_NA | 0.18541 | 0.001 |
| -0.30 | -0.31 | 519_NA | 0.18333 | 0.001 |
| 0.42 | -0.08 | 668_Chlorogenic acid | 0.18024 | 0.001 |
| 0.16 | 0.39 | 590_NA | 0.17808 | 0.001 |
| -0.14 | 0.40 | 761_NA | 0.17653 | 0.001 |
| -0.10 | -0.40 | 921_NA | 0.17374 | 0.001 |
| 0.16 | 0.38 | 706_NA | 0.16834 | 0.001 |
| 0.14 | 0.38 | 12_NA | 0.16800 | 0.001 |
| -0.14 | 0.37 | 621_NA | 0.15914 | 0.001 |
| -0.28 | 0.53 | 908_NA | 0.36399 | 0.002 |
| 0.50 | -0.07 | 121_NA | 0.25184 | 0.002 |
| -0.06 | -0.49 | 513_NA | 0.24617 | 0.002 |
| 0.12 | 0.47 | 262_NA | 0.23606 | 0.002 |
| -0.47 | -0.03 | 641_NA | 0.22368 | 0.002 |
| -0.01 | 0.43 | 554_NA | 0.18091 | 0.002 |
| 0.35 | 0.24 | 14_2-Hydroxypropanoic acid (Lactic acid) | 0.18004 | 0.002 |
| 0.41 | 0.09 | 329_*Myo*-Inositol | 0.17953 | 0.002 |
| -0.42 | 0.04 | 680_NA | 0.17564 | 0.002 |
| 0.41 | -0.01 | 690_NA | 0.17215 | 0.002 |
| -0.21 | -0.35 | 211_NA | 0.16860 | 0.002 |
| -0.09 | 0.39 | 779_NA | 0.16164 | 0.002 |
| -0.40 | -0.02 | 907_Nonadecanenitrile | 0.16142 | 0.002 |
| -0.33 | -0.23 | 467_NA | 0.16099 | 0.002 |

| PCoA Axis1 | PCoA Axis2 | Feature | $R^2$ | p-value |
|---|---|---|---|---|
| 0.35 | -0.20 | 390_NA | 0.15987 | 0.002 |
| -0.31 | -0.24 | 677_NA | 0.15260 | 0.002 |
| -0.35 | -0.17 | 766_NA | 0.15081 | 0.002 |
| 0.11 | 0.42 | 136_NA | 0.19250 | 0.003 |
| 0.09 | 0.41 | 520_NA | 0.17573 | 0.003 |
| 0.28 | 0.25 | 237_NA | 0.14262 | 0.003 |
| 0.34 | 0.13 | 228_Aconitic acid | 0.13567 | 0.003 |
| -0.30 | 0.24 | 728_NA | 0.14306 | 0.004 |
| 0.25 | 0.28 | 247_*p*-Coumaric acid | 0.14016 | 0.004 |
| 0.37 | -0.05 | 559_NA | 0.13614 | 0.004 |
| 0.36 | 0.06 | 546_NA | 0.13440 | 0.004 |
| -0.36 | 0.00 | 900_Tritriacontanoic acid (Psyllic acid) | 0.12910 | 0.004 |
| -0.06 | 0.35 | 848_NA | 0.12425 | 0.004 |
| 0.37 | 0.00 | 537_Mannobiose | 0.14007 | 0.005 |
| -0.33 | 0.16 | 284_NA | 0.13151 | 0.005 |
| 0.36 | -0.01 | 463_NA | 0.12971 | 0.007 |
| -0.35 | 0.10 | 830_NA | 0.13110 | 0.008 |
| 0.08 | 0.33 | 344_NA | 0.11860 | 0.008 |
| 0.20 | 0.29 | 659_NA | 0.12795 | 0.009 |
| -0.19 | 0.28 | 594_NA | 0.11228 | 0.010 |
| 0.33 | -0.07 | 272_NA | 0.11088 | 0.010 |
| -0.22 | 0.27 | 385_NA | 0.12047 | 0.011 |
| 0.31 | -0.13 | 217_Arabitol | 0.11347 | 0.011 |
| 0.01 | 0.33 | 179_NA | 0.11071 | 0.012 |
| 0.24 | -0.23 | 504_NA | 0.10931 | 0.012 |
| -0.31 | -0.11 | 922_NA | 0.10944 | 0.013 |
| -0.02 | -0.32 | 757_NA | 0.10074 | 0.016 |
| 0.15 | 0.29 | 388_NA | 0.10785 | 0.017 |
| -0.25 | -0.21 | 619_NA | 0.10354 | 0.017 |
| 0.08 | 0.31 | 622_NA | 0.10129 | 0.017 |
| 0.33 | -0.01 | 801_NA | 0.10636 | 0.019 |
| 0.14 | 0.28 | 553_NA | 0.09937 | 0.020 |
| -0.30 | 0.02 | 364_Heneicosane | 0.09213 | 0.022 |
| 0.29 | -0.05 | 186_NA | 0.08407 | 0.022 |
| 0.24 | 0.16 | 500_NA | 0.08248 | 0.022 |
| 0.29 | -0.05 | 794_NA | 0.08781 | 0.028 |
| 0.25 | -0.17 | 555_Trehalose | 0.09146 | 0.030 |
| 0.23 | 0.18 | 68_NA | 0.08638 | 0.040 |
| -0.27 | 0.10 | 579_NA | 0.08484 | 0.040 |

| PCoA Axis1 | PCoA Axis2 | Feature | $R^2$ | p-value |
|---|---|---|---|---|
| 0.03 | 0.28 | 589_NA | 0.08173 | 0.041 |
| 0.27 | 0.09 | 113_1,2,3-Butanetriol | 0.07911 | 0.042 |
| 0.26 | -0.05 | 85_NA | 0.07209 | 0.045 |

## Table S4. Spearman Correlations between Metabolite Intensities and PCoA Axes[a]

| Feature | PCoA Axis 1 (r) | p-value _PCoA1 | PCoA Axis 2 (r) | p-value _PCoA2 | max_abs _cor |
|---|---|---|---|---|---|
| 605_Nonacosane | -0.945 | 0 | -0.051 | 0.663 | 0.945 |
| 312_NA | 0.914 | 0 | -0.015 | 0.899 | 0.914 |
| 440_Nonadecanoic acid (Nonadecylic acid) | -0.91 | 0 | -0.084 | 0.47 | 0.91 |
| 492_NA | -0.903 | 0 | 0.005 | 0.965 | 0.903 |
| 556_Tricosanoic acid (Tricosylic acid) | -0.9 | 0 | 0.139 | 0.233 | 0.9 |
| 744_Stigmastanol | -0.892 | 0 | 0.099 | 0.398 | 0.892 |
| 294_Glucitol | 0.892 | 0 | -0.091 | 0.438 | 0.892 |
| 548_Heptacosane | -0.888 | 0 | -0.184 | 0.114 | 0.888 |
| 292_Mannose | 0.887 | 0 | 0.101 | 0.388 | 0.887 |
| 613_Pentacosanoic acid (Pentacosylic acid) | -0.886 | 0 | 0.287 | 0.013 | 0.886 |
| 287_Talose | 0.883 | 0 | -0.111 | 0.341 | 0.883 |
| 155_Pyroglutamic acid | 0.881 | 0 | -0.028 | 0.813 | 0.881 |
| 215_NA | 0.873 | 0 | -0.149 | 0.2 | 0.873 |
| 317_Gluconic acid | 0.871 | 0 | 0.006 | 0.962 | 0.871 |
| 724_Stigmasterol | -0.87 | 0 | -0.069 | 0.556 | 0.87 |
| 792_NA | -0.322 | 0.005 | 0.867 | 0 | 0.867 |
| 503_Heneicosanoic acid (Heneicosylic acid) | -0.859 | 0 | 0.328 | 0.004 | 0.859 |
| 713_Campesterol | -0.855 | 0 | 0.002 | 0.984 | 0.855 |
| 437_NA | -0.851 | 0 | -0.047 | 0.686 | 0.851 |
| 366_NA | 0.85 | 0 | -0.082 | 0.482 | 0.85 |
| 524_Docosanoic acid (Behenic acid) | -0.846 | 0 | 0.282 | 0.014 | 0.846 |
| 300_NA | 0.844 | 0 | -0.006 | 0.961 | 0.844 |
| 329_*Myo*-Inositol | 0.842 | 0 | 0.163 | 0.162 | 0.842 |
| 208_Xylitol | 0.84 | 0 | 0.138 | 0.237 | 0.84 |
| 741_*β*-Sitosterol | -0.838 | 0 | 0.255 | 0.027 | 0.838 |
| 27_Hydroxyacetic acid (Glycolic acid) | 0.836 | 0 | 0.024 | 0.838 | 0.836 |
| 420_NA | -0.311 | 0.007 | 0.835 | 0 | 0.835 |
| 275_Fructose | 0.83 | 0 | -0.046 | 0.691 | 0.83 |
| 551_NA | 0.829 | 0 | -0.105 | 0.37 | 0.829 |
| 280_NA | 0.827 | 0 | -0.083 | 0.479 | 0.827 |
| 735_NA | -0.824 | 0 | -0.288 | 0.012 | 0.824 |
| 156_*γ*-Aminobutanoic acid (GABA) | 0.822 | 0 | 0.236 | 0.042 | 0.822 |
| 144_Ascorbic acid | 0.822 | 0 | 0.093 | 0.426 | 0.822 |
| 738_NA | -0.821 | 0 | 0.054 | 0.644 | 0.821 |
| 158_Threonic acid | 0.812 | 0 | 0.193 | 0.097 | 0.812 |
| 268_NA | 0.81 | 0 | 0.004 | 0.974 | 0.81 |

| Feature | PCoA Axis 1 (r) | p-value _PCoA1 | PCoA Axis 2 (r) | p-value _PCoA2 | max_abs _cor |
|---|---|---|---|---|---|
| 671_NA | -0.808 | 0 | -0.378 | 0.001 | 0.808 |
| 814_NA | -0.805 | 0 | -0.167 | 0.151 | 0.805 |
| 441_NA | 0.8 | 0 | -0.208 | 0.073 | 0.8 |
| 242_Vanillic acid | 0.8 | 0 | -0.085 | 0.467 | 0.8 |
| 603_NA | -0.013 | 0.914 | -0.799 | 0 | 0.799 |
| 817_1-Dotriacontanol | -0.416 | 0 | 0.798 | 0 | 0.798 |
| 700_Octacosanoic acid (Montanic acid) | -0.798 | 0 | 0.347 | 0.002 | 0.798 |
| 644_Hexacosanoic acid (Cerotic acid) | -0.797 | 0 | 0.193 | 0.097 | 0.797 |
| 676_1-Octacosanol | -0.795 | 0 | 0.112 | 0.338 | 0.795 |
| 649_NA | -0.091 | 0.435 | 0.792 | 0 | 0.792 |
| 477_Eicosanoic acid (Arachidic acid) | -0.79 | 0 | 0.083 | 0.48 | 0.79 |
| 586_Tetracosanoic acid (Lignoceric acid) | -0.788 | 0 | 0.343 | 0.003 | 0.788 |
| 717_NA | -0.786 | 0 | -0.154 | 0.185 | 0.786 |
| 351_*Myo*-Inositol | 0.785 | 0 | -0.034 | 0.771 | 0.785 |
| 611_NA | -0.779 | 0 | -0.555 | 0 | 0.779 |
| 109_2,3-Dihydroxypropanoic acid (Glyceric acid) | 0.779 | 0 | 0.25 | 0.031 | 0.779 |
| 20_NA | -0.775 | 0 | -0.225 | 0.052 | 0.775 |
| 432_NA | -0.761 | 0 | -0.163 | 0.163 | 0.761 |
| 727_NA | -0.761 | 0 | -0.117 | 0.316 | 0.761 |
| 614_1-Hexacosanol | -0.76 | 0 | -0.136 | 0.246 | 0.76 |
| 701_NA | -0.759 | 0 | -0.006 | 0.962 | 0.759 |
| 818_NA | -0.175 | 0.134 | 0.755 | 0 | 0.755 |
| 429_Tricosane | -0.755 | 0 | 0.445 | 0 | 0.755 |
| 564_NA | 0.755 | 0 | 0.054 | 0.647 | 0.755 |
| 516_NA | -0.754 | 0 | 0.159 | 0.173 | 0.754 |
| 234_Ribonic acid | 0.752 | 0 | 0.343 | 0.003 | 0.752 |
| 608_Nonacosane | 0.751 | 0 | 0.367 | 0.001 | 0.751 |
| 206_NA | 0.747 | 0 | -0.004 | 0.976 | 0.747 |
| 512_NA | 0.744 | 0 | 0.33 | 0.004 | 0.744 |
| 600_NA | 0.739 | 0 | 0.171 | 0.143 | 0.739 |
| 237_NA | 0.738 | 0 | 0.278 | 0.016 | 0.738 |
| 295_NA | 0.737 | 0 | 0.153 | 0.189 | 0.737 |
| 795_Glutinol | -0.39 | 0.001 | 0.726 | 0 | 0.726 |
| 256_NA | 0.722 | 0 | 0.157 | 0.179 | 0.722 |
| 338_Hexadecanoic acid (Palmitic acid) | -0.72 | 0 | -0.151 | 0.194 | 0.72 |
| 386_Ethyl palmitate | 0.037 | 0.754 | -0.719 | 0 | 0.719 |
| 165_NA | 0.715 | 0 | -0.044 | 0.709 | 0.715 |
| 566_NA | -0.148 | 0.206 | 0.712 | 0 | 0.712 |

| Feature | PCoA Axis 1 (r) | p-value _PCoA1 | PCoA Axis 2 (r) | p-value _PCoA2 | max_abs _cor |
|---|---|---|---|---|---|
| 191_Arabinose | 0.712 | 0 | -0.131 | 0.262 | 0.712 |
| 612_NA | 0.711 | 0 | 0.036 | 0.76 | 0.711 |
| 710_Heptacosanal | 0.704 | 0 | 0.346 | 0.003 | 0.704 |
| 150_Threitol | 0.699 | 0 | 0.209 | 0.072 | 0.699 |
| 149_Threitol | 0.698 | 0 | 0.263 | 0.023 | 0.698 |
| 558_NA | -0.698 | 0 | 0.243 | 0.036 | 0.698 |
| 188_NA | 0.698 | 0 | -0.11 | 0.349 | 0.698 |
| 461_NA | -0.247 | 0.033 | -0.697 | 0 | 0.697 |
| 269_NA | -0.324 | 0.005 | 0.695 | 0 | 0.695 |
| 5_Propane-1,2-diol (Propylene glycol) | 0.69 | 0 | 0.247 | 0.033 | 0.69 |
| 785_Triacontanoic acid (Melissic acid) | -0.689 | 0 | 0.385 | 0.001 | 0.689 |
| 451_NA | -0.267 | 0.021 | -0.687 | 0 | 0.687 |
| 356_*Myo*-Inositol | 0.684 | 0 | -0.167 | 0.151 | 0.684 |
| 483_NA | -0.385 | 0.001 | -0.678 | 0 | 0.678 |
| 679_Cholesterol | -0.61 | 0 | 0.677 | 0 | 0.677 |
| 299_NA | -0.676 | 0 | -0.059 | 0.614 | 0.676 |
| 111_2-Butenedioic acid (Fumaric acid) | 0.676 | 0 | 0.009 | 0.936 | 0.676 |
| 695_NA | -0.128 | 0.272 | -0.673 | 0 | 0.673 |
| 469_NA | -0.513 | 0 | -0.67 | 0 | 0.67 |
| 21_2-Hydroxypropanoic acid (Lactic Acid) | 0.669 | 0 | 0.368 | 0.001 | 0.669 |
| 95_Propane-1,2,3-triol (Glycerol) | 0.667 | 0 | -0.078 | 0.505 | 0.667 |
| 523_Sucrose | 0.666 | 0 | 0.15 | 0.2 | 0.666 |
| 729_Tritriacontane | -0.527 | 0 | 0.663 | 0 | 0.663 |
| 31_2-Oxopropanoic acid (Pyruvic acid) | 0.663 | 0 | 0.142 | 0.224 | 0.663 |
| 811_NA | 0.661 | 0 | 0.201 | 0.084 | 0.661 |
| 14_2-Hydroxypropanoic acid (Lactic acid) | 0.658 | 0 | 0.236 | 0.042 | 0.658 |
| 385_NA | -0.152 | 0.191 | 0.657 | 0 | 0.657 |
| 630_NA | -0.56 | 0 | 0.655 | 0 | 0.655 |
| 131_3,4-Dihydroxybutanoic acid | 0.654 | 0 | 0.076 | 0.515 | 0.654 |
| 374_Methyl 2-hydroxystearate | -0.653 | 0 | 0.024 | 0.838 | 0.653 |
| 283_NA | 0.022 | 0.852 | 0.649 | 0 | 0.649 |
| 293_Pentadecanoic acid (Pentadecylic acid) | -0.649 | 0 | 0.572 | 0 | 0.649 |
| 120_Threonine | 0.649 | 0 | 0.246 | 0.034 | 0.649 |
| 465_NA | -0.647 | 0 | 0.275 | 0.017 | 0.647 |
| 876_NA | -0.647 | 0 | -0.054 | 0.644 | 0.647 |
| 16_Butane-2,3-diol | 0.646 | 0 | 0.324 | 0.005 | 0.646 |
| 582_NA | 0.645 | 0 | -0.263 | 0.023 | 0.645 |
| 393_(9*Z*)-Octadecenoic acid (Oleic acid) | -0.644 | 0 | 0.105 | 0.369 | 0.644 |

| Feature | PCoA Axis 1 (r) | p-value _PCoA1 | PCoA Axis 2 (r) | p-value _PCoA2 | max_abs _cor |
|---|---|---|---|---|---|
| 560_1-Oleoylglycerol (1-monoolein) | 0.192 | 0.098 | -0.643 | 0 | 0.643 |
| 392_(9*Z*)-Octadecenoic acid (Oleic acid) | -0.323 | 0.005 | -0.642 | 0 | 0.642 |
| 478_Eicosanoic acid (Arachidic acid) | -0.258 | 0.025 | -0.64 | 0 | 0.64 |
| 194_NA | 0.64 | 0 | 0.094 | 0.423 | 0.64 |
| 319_Hexadecanoic acid (Palmitic acid) | -0.327 | 0.004 | 0.638 | 0 | 0.638 |
| 667_Hentriacontane | -0.638 | 0 | 0.597 | 0 | 0.638 |
| 297_*p*-Coumaric acid | 0.633 | 0 | 0.49 | 0 | 0.633 |
| 798_Lanosterol | -0.46 | 0 | 0.632 | 0 | 0.632 |
| 740_1-Triacontanol | -0.632 | 0 | 0.493 | 0 | 0.632 |
| 378_Phytol | -0.287 | 0.013 | 0.631 | 0 | 0.631 |
| 103_1,2,3-Butanetriol | 0.631 | 0 | -0.087 | 0.459 | 0.631 |
| 849_NA | -0.628 | 0 | -0.164 | 0.159 | 0.628 |
| 389_NA | 0.627 | 0 | 0.191 | 0.1 | 0.627 |
| 383_NA | 0.626 | 0 | -0.013 | 0.912 | 0.626 |
| 668_Chlorogenic acid | 0.626 | 0 | -0.008 | 0.947 | 0.626 |
| 923_NA | -0.341 | 0.003 | 0.624 | 0 | 0.624 |
| 694_NA | -0.542 | 0 | 0.623 | 0 | 0.623 |
| 658_NA | -0.622 | 0 | 0.233 | 0.045 | 0.622 |
| 496_NA | -0.245 | 0.034 | -0.621 | 0 | 0.621 |
| 55_3-Hydroxypropanoic acid (Hydracrylic acid) | -0.621 | 0 | -0.185 | 0.113 | 0.621 |
| 108_2,3-Diaminopropionic acid (3-Amino-alanine) | 0.62 | 0 | -0.103 | 0.379 | 0.62 |
| 641_NA | -0.619 | 0 | 0.005 | 0.965 | 0.619 |
| 313_NA | 0.616 | 0 | 0.218 | 0.06 | 0.616 |
| 824_NA | -0.615 | 0 | -0.243 | 0.036 | 0.615 |
| 404_NA | 0.614 | 0 | -0.105 | 0.367 | 0.614 |
| 370_Heptadecanoic acid (Margaric acid) | -0.614 | 0 | 0.056 | 0.636 | 0.614 |
| 764_NA | -0.297 | 0.01 | -0.613 | 0 | 0.613 |
| 173_2-Deoxyarabitol | 0.179 | 0.125 | -0.613 | 0 | 0.613 |
| 569_NA | 0.607 | 0 | -0.114 | 0.331 | 0.607 |
| 318_4-Hydroxy-3-methoxymandelic acid | 0.51 | 0 | 0.606 | 0 | 0.606 |
| 316_NA | 0.606 | 0 | 0.369 | 0.001 | 0.606 |
| 555_Trehalose | 0.605 | 0 | -0.235 | 0.043 | 0.605 |
| 665_NA | -0.308 | 0.007 | 0.602 | 0 | 0.602 |
| 945_NA | -0.13 | 0.265 | -0.599 | 0 | 0.599 |
| 578_NA | -0.597 | 0 | 0.236 | 0.042 | 0.597 |
| 434_NA | 0.595 | 0 | -0.185 | 0.112 | 0.595 |
| 227_NA | 0.594 | 0 | 0.131 | 0.262 | 0.594 |
| 230_3,6-Anhydrogalactose | 0.591 | 0 | 0.128 | 0.273 | 0.591 |

| Feature | PCoA Axis 1 (r) | p-value _PCoA1 | PCoA Axis 2 (r) | p-value _PCoA2 | max_abs _cor |
|---|---|---|---|---|---|
| 647_NA | -0.542 | 0 | -0.588 | 0 | 0.588 |
| 802_NA | -0.192 | 0.099 | 0.587 | 0 | 0.587 |
| 325_14-Methylhexadecanoic acid | -0.586 | 0 | -0.219 | 0.06 | 0.586 |
| 58_3-Hydroxybutanoic acid ($\beta$-Hydroxybutyric acid,) | 0.584 | 0 | 0.152 | 0.192 | 0.584 |
| 255_Citric acid | 0.583 | 0 | 0.335 | 0.003 | 0.583 |
| 454_NA | 0.58 | 0 | -0.029 | 0.804 | 0.58 |
| 680_NA | -0.579 | 0 | 0.289 | 0.012 | 0.579 |
| 760_NA | -0.5 | 0 | -0.578 | 0 | 0.578 |
| 617_NA | -0.577 | 0 | 0.255 | 0.027 | 0.577 |
| 796_NA | 0.574 | 0 | 0.289 | 0.012 | 0.574 |
| 362_NA | -0.572 | 0 | 0.264 | 0.022 | 0.572 |
| 547_NA | 0.572 | 0 | -0.241 | 0.038 | 0.572 |
| 761_NA | -0.572 | 0 | 0.166 | 0.153 | 0.572 |
| 799_NA | -0.57 | 0 | -0.207 | 0.075 | 0.57 |
| 599_NA | -0.57 | 0 | 0.12 | 0.305 | 0.57 |
| 118_NA | 0.568 | 0 | -0.269 | 0.02 | 0.568 |
| 793_NA | -0.378 | 0.001 | -0.567 | 0 | 0.567 |
| 199_NA | 0.172 | 0.14 | 0.565 | 0 | 0.565 |
| 549_2-Oleoylglycerol (2-monoolein) | -0.183 | 0.117 | -0.563 | 0 | 0.563 |
| 507_NA | 0.563 | 0 | -0.452 | 0 | 0.563 |
| 907_Nonadecanenitrile | -0.562 | 0 | 0.075 | 0.522 | 0.562 |
| 565_NA | 0.555 | 0 | -0.158 | 0.176 | 0.555 |
| 753_NA | -0.42 | 0 | 0.554 | 0 | 0.554 |
| 755_NA | -0.315 | 0.006 | -0.549 | 0 | 0.549 |
| 405_galactinol | 0.544 | 0 | 0.054 | 0.643 | 0.544 |
| 546_NA | 0.543 | 0 | -0.086 | 0.463 | 0.543 |
| 528_NA | 0.543 | 0 | 0.004 | 0.971 | 0.543 |
| 257_3,4-Dihydroxybenzoic acid (Protocatechuic acid) | 0.157 | 0.179 | -0.54 | 0 | 0.54 |
| 659_NA | 0.54 | 0 | 0.113 | 0.334 | 0.54 |
| 900_Tritriacontanoic acid (Psyllic acid) | -0.54 | 0 | -0.019 | 0.868 | 0.54 |
| 425_NA | 0.539 | 0 | 0.228 | 0.049 | 0.539 |
| 361_Heptadecanoic acid (Margaric acid) | -0.476 | 0 | 0.537 | 0 | 0.537 |
| 229_NA | 0.535 | 0 | 0.081 | 0.487 | 0.535 |
| 542_NA | -0.127 | 0.278 | 0.534 | 0 | 0.534 |
| 535_NA | 0.529 | 0 | -0.259 | 0.025 | 0.529 |
| 121_NA | 0.528 | 0 | -0.147 | 0.208 | 0.528 |
| 711_5-Methoxysalicylic acid | -0.517 | 0 | -0.526 | 0 | 0.526 |
| 281_Glucose | 0.523 | 0 | -0.129 | 0.27 | 0.523 |

| Feature | PCoA Axis 1 (r) | p-value _PCoA1 | PCoA Axis 2 (r) | p-value _PCoA2 | max_abs _cor |
|---|---|---|---|---|---|
| 815_Hentriacontanoic acid (Hentriacontylic acid) | -0.516 | 0 | 0.522 | 0 | 0.522 |
| 703_NA | -0.521 | 0 | -0.129 | 0.269 | 0.521 |
| 956_NA | -0.059 | 0.612 | -0.519 | 0 | 0.519 |
| 903_Isobutyl tetratriacontyl ether | -0.156 | 0.18 | 0.517 | 0 | 0.517 |
| 468_NA | -0.194 | 0.096 | 0.512 | 0 | 0.512 |
| 537_Mannobiose | 0.511 | 0 | -0.106 | 0.367 | 0.511 |
| 488_NA | 0.509 | 0 | -0.207 | 0.075 | 0.509 |
| 298_NA | 0.509 | 0 | -0.159 | 0.172 | 0.509 |
| 247_*p*-Coumaric acid | 0.508 | 0 | 0.375 | 0.001 | 0.508 |
| 75_NA | 0.507 | 0 | 0.108 | 0.356 | 0.507 |
| 220_NA | 0.506 | 0 | 0.423 | 0 | 0.506 |
| 485_NA | -0.506 | 0 | -0.276 | 0.017 | 0.506 |
| 284_NA | -0.372 | 0.001 | 0.504 | 0 | 0.504 |
| 531_NA | 0.017 | 0.887 | -0.504 | 0 | 0.504 |
| 428_NA | 0.5 | 0 | -0.263 | 0.023 | 0.5 |
| 568_NA | -0.279 | 0.016 | 0.499 | 0 | 0.499 |
| 364_Heneicosane | -0.498 | 0 | 0.242 | 0.037 | 0.498 |
| 882_NA | -0.123 | 0.292 | -0.495 | 0 | 0.495 |
| 489_NA | 0.492 | 0 | -0.015 | 0.899 | 0.492 |
| 584_(*Z*)-13-Docosenamide | -0.172 | 0.141 | 0.49 | 0 | 0.49 |
| 228_Aconitic acid | 0.489 | 0 | -0.148 | 0.205 | 0.489 |
| 728_NA | -0.294 | 0.011 | 0.488 | 0 | 0.488 |
| 83_Urea | 0.336 | 0.003 | 0.485 | 0 | 0.485 |
| 365_2-Methylpentadecanoic acid | -0.483 | 0 | 0.47 | 0 | 0.483 |
| 391_(9*Z*)-Octadecenoic acid (Oleic acid) | -0.479 | 0 | -0.01 | 0.931 | 0.479 |
| 908_NA | -0.23 | 0.048 | 0.478 | 0 | 0.478 |
| 368_Mannitol | 0.477 | 0 | -0.078 | 0.507 | 0.477 |
| 285_NA | 0.475 | 0 | 0.218 | 0.06 | 0.475 |
| 830_NA | -0.474 | 0 | 0.24 | 0.038 | 0.474 |
| 766_NA | -0.474 | 0 | -0.145 | 0.213 | 0.474 |
| 67_NA | 0.473 | 0 | 0.195 | 0.093 | 0.473 |
| 196_NA | 0.472 | 0 | -0.259 | 0.025 | 0.472 |
| 648_1,2-Epoxyoctadecane | -0.165 | 0.157 | -0.465 | 0 | 0.465 |
| 330_NA | -0.314 | 0.006 | -0.462 | 0 | 0.462 |
| 677_NA | -0.279 | 0.016 | -0.462 | 0 | 0.462 |
| 804_NA | 0.462 | 0 | -0.065 | 0.579 | 0.462 |
| 770_NA | -0.154 | 0.186 | 0.455 | 0 | 0.455 |
| 514_NA | -0.455 | 0 | 0.374 | 0.001 | 0.455 |

| Feature | PCoA Axis 1 (r) | p-value _PCoA1 | PCoA Axis 2 (r) | p-value _PCoA2 | max_abs _cor |
|---|---|---|---|---|---|
| 609_NA | -0.207 | 0.075 | 0.452 | 0 | 0.452 |
| 500_NA | 0.449 | 0 | -0.262 | 0.023 | 0.449 |
| 618_NA | -0.447 | 0 | -0.105 | 0.367 | 0.447 |
| 398_NA | 0.443 | 0 | -0.126 | 0.28 | 0.443 |
| 554_NA | -0.439 | 0 | 0.219 | 0.059 | 0.439 |
| 116_NA | 0.438 | 0 | 0.115 | 0.324 | 0.438 |
| 423_NA | 0.436 | 0 | 0.343 | 0.003 | 0.436 |
| 70_Propanedioic acid (Malonic acid) | 0.434 | 0 | -0.077 | 0.511 | 0.434 |
| 502_NA | 0.433 | 0 | -0.377 | 0.001 | 0.433 |
| 305_Galactose | 0.372 | 0.001 | 0.423 | 0 | 0.423 |
| 739_NA | -0.422 | 0 | -0.299 | 0.01 | 0.422 |
| 498_NA | 0.422 | 0 | -0.112 | 0.336 | 0.422 |
| 262_NA | 0.421 | 0 | 0.326 | 0.004 | 0.421 |
| 296_NA | 0.317 | 0.006 | -0.42 | 0 | 0.42 |
| 49_1,2-Ethanedioic acid (Oxalic acid) | -0.301 | 0.009 | -0.413 | 0 | 0.413 |
| 559_NA | 0.412 | 0 | 0.251 | 0.03 | 0.412 |
| 403_Octadecanoic acid (Stearic acid) | -0.409 | 0 | -0.403 | 0 | 0.409 |
| 71_NA | 0.155 | 0.185 | 0.409 | 0 | 0.409 |
| 270_Tetradecanoic acid (Myristic acid) | -0.395 | 0.001 | 0.408 | 0 | 0.408 |
| 113_1,2,3-Butanetriol | 0.399 | 0 | -0.005 | 0.968 | 0.399 |
| 759_NA | -0.256 | 0.027 | 0.398 | 0 | 0.398 |
| 521_NA | -0.07 | 0.552 | 0.398 | 0 | 0.398 |
| 90_NA | 0.394 | 0.001 | 0.194 | 0.095 | 0.394 |
| 439_NA | 0.394 | 0.001 | 0.159 | 0.172 | 0.394 |
| 674_NA | 0.394 | 0.001 | -0.109 | 0.352 | 0.394 |
| 590_NA | -0.068 | 0.562 | 0.39 | 0.001 | 0.39 |
| 136_NA | 0.388 | 0.001 | 0.364 | 0.001 | 0.388 |
| 922_NA | -0.252 | 0.029 | -0.387 | 0.001 | 0.387 |
| 806_NA | -0.195 | 0.094 | -0.385 | 0.001 | 0.385 |
| 119_NA | 0.086 | 0.463 | 0.384 | 0.001 | 0.384 |
| 865_NA | -0.196 | 0.092 | 0.383 | 0.001 | 0.383 |
| 805_NA | -0.376 | 0.001 | -0.327 | 0.004 | 0.376 |
| 520_NA | 0.044 | 0.709 | 0.373 | 0.001 | 0.373 |
| 411_NA | -0.37 | 0.001 | 0.277 | 0.016 | 0.37 |
| 583_NA | 0.37 | 0.001 | 0.151 | 0.194 | 0.37 |
| 929_NA | -0.369 | 0.001 | 0.33 | 0.004 | 0.369 |
| 899_NA | -0.369 | 0.001 | -0.273 | 0.018 | 0.369 |
| 273_NA | 0.368 | 0.001 | -0.336 | 0.003 | 0.368 |

| Feature | PCoA Axis 1 (r) | p-value _PCoA1 | PCoA Axis 2 (r) | p-value _PCoA2 | max_abs _cor |
|---|---|---|---|---|---|
| 562_NA | -0.131 | 0.26 | -0.366 | 0.001 | 0.366 |
| 924_NA | 0.027 | 0.815 | 0.366 | 0.001 | 0.366 |
| 267_NA | 0.063 | 0.593 | 0.365 | 0.001 | 0.365 |
| 621_NA | -0.028 | 0.812 | 0.364 | 0.001 | 0.364 |
| 217_Arabitol | 0.361 | 0.002 | -0.02 | 0.868 | 0.361 |
| 218_NA | 0.039 | 0.738 | 0.358 | 0.002 | 0.358 |
| 394_(9*Z*)-Octadecenoic acid (Oleic acid) | -0.042 | 0.723 | -0.357 | 0.002 | 0.357 |
| 901_1-Tetratriacontanol | -0.357 | 0.002 | -0.132 | 0.26 | 0.357 |
| 271_Adonitol | 0.068 | 0.562 | -0.351 | 0.002 | 0.351 |
| 337_NA | 0.232 | 0.046 | 0.351 | 0.002 | 0.351 |
| 505_NA | -0.171 | 0.142 | 0.348 | 0.002 | 0.348 |
| 794_NA | 0.346 | 0.002 | -0.166 | 0.155 | 0.346 |
| 467_NA | -0.346 | 0.002 | -0.059 | 0.615 | 0.346 |
| 847_NA | -0.345 | 0.003 | 0.046 | 0.697 | 0.345 |
| 50_1,2-Ethanedioic acid (Oxalic acid) | 0.341 | 0.003 | 0.129 | 0.27 | 0.341 |
| 863_NA | 0.264 | 0.023 | -0.334 | 0.004 | 0.334 |
| 486_NA | -0.138 | 0.236 | 0.321 | 0.005 | 0.321 |
| 186_NA | 0.318 | 0.006 | 0.09 | 0.441 | 0.318 |
| 757_NA | -0.014 | 0.902 | -0.315 | 0.006 | 0.315 |
| 388_NA | 0.083 | 0.479 | 0.315 | 0.006 | 0.315 |
| 431_NA | 0.312 | 0.007 | 0.119 | 0.308 | 0.312 |
| 594_NA | -0.123 | 0.291 | 0.311 | 0.007 | 0.311 |
| 379_NA | -0.207 | 0.075 | -0.304 | 0.008 | 0.304 |
| 844_NA | -0.304 | 0.008 | 0.142 | 0.223 | 0.304 |
| 552_NA | 0.187 | 0.107 | -0.303 | 0.008 | 0.303 |
| 853_NA | 0.04 | 0.732 | 0.303 | 0.008 | 0.303 |
| 601_NA | -0.243 | 0.036 | 0.302 | 0.009 | 0.302 |
| 376_NA | -0.158 | 0.176 | -0.301 | 0.009 | 0.301 |
| 579_NA | -0.251 | 0.03 | 0.291 | 0.012 | 0.291 |
| 767_NA | -0.284 | 0.014 | -0.027 | 0.817 | 0.284 |
| 85_NA | -0.283 | 0.014 | 0.188 | 0.106 | 0.283 |
| 918_NA | -0.04 | 0.732 | 0.28 | 0.015 | 0.28 |
| 689_NA | 0.279 | 0.016 | 0.178 | 0.126 | 0.279 |
| 179_NA | -0.278 | 0.016 | 0.042 | 0.718 | 0.278 |
| 848_NA | -0.074 | 0.53 | 0.275 | 0.017 | 0.275 |
| 73_3-Hydroxyisovaleric acid | 0.271 | 0.019 | 0 | 0.999 | 0.271 |
| 602_NA | 0.27 | 0.02 | -0.194 | 0.095 | 0.27 |
| 122_2-Hydroxybutanoic acid ($\alpha$-hydroxybutyric acid) | 0.268 | 0.021 | -0.122 | 0.297 | 0.268 |

| Feature | PCoA Axis 1 (r) | p-value _PCoA1 | PCoA Axis 2 (r) | p-value _PCoA2 | max_abs _cor |
|---|---|---|---|---|---|
| 690_NA | 0.264 | 0.022 | 0.097 | 0.405 | 0.264 |
| 499_NA | -0.1 | 0.393 | -0.261 | 0.024 | 0.261 |
| 504_NA | 0.252 | 0.029 | 0.038 | 0.743 | 0.252 |
| 340_NA | -0.251 | 0.03 | 0.124 | 0.287 | 0.251 |
| 198_NA | 0.21 | 0.07 | 0.25 | 0.031 | 0.25 |
| 670_NA | 0.246 | 0.034 | -0.077 | 0.508 | 0.246 |
| 513_NA | 0.241 | 0.037 | -0.15 | 0.198 | 0.241 |
| 884_NA | -0.237 | 0.041 | 0.217 | 0.061 | 0.237 |
| 272_NA | 0.236 | 0.041 | -0.008 | 0.945 | 0.236 |
| 344_NA | 0.102 | 0.384 | 0.23 | 0.048 | 0.23 |
| 683_NA | -0.088 | 0.45 | 0.229 | 0.048 | 0.229 |
| 553_NA | 0.229 | 0.049 | 0.144 | 0.218 | 0.229 |
| 539_NA | -0.107 | 0.362 | 0.228 | 0.05 | 0.228 |
| 68_NA | 0.226 | 0.051 | -0.062 | 0.596 | 0.226 |
| 638_NA | -0.224 | 0.053 | 0.059 | 0.613 | 0.224 |
| 598_NA | 0.221 | 0.057 | -0.164 | 0.161 | 0.221 |
| 779_NA | -0.085 | 0.466 | 0.219 | 0.059 | 0.219 |
| 619_NA | -0.217 | 0.062 | -0.013 | 0.911 | 0.217 |
| 540_Sucrose | 0.091 | 0.436 | 0.21 | 0.07 | 0.21 |
| 854_NA | -0.064 | 0.587 | 0.209 | 0.071 | 0.209 |
| 801_NA | 0.022 | 0.848 | 0.208 | 0.074 | 0.208 |
| 706_NA | 0.068 | 0.56 | 0.205 | 0.078 | 0.205 |
| 758_NA | 0.171 | 0.142 | -0.199 | 0.087 | 0.199 |
| 947_NA | -0.129 | 0.268 | 0.198 | 0.088 | 0.198 |
| 883_NA | -0.197 | 0.09 | 0.174 | 0.136 | 0.197 |
| 12_NA | 0.026 | 0.827 | -0.193 | 0.097 | 0.193 |
| 850_NA | -0.157 | 0.179 | 0.193 | 0.098 | 0.193 |
| 593_Melibiose | 0.193 | 0.098 | -0.144 | 0.216 | 0.193 |
| 470_NA | -0.19 | 0.103 | 0.066 | 0.574 | 0.19 |
| 166_NA | 0.185 | 0.113 | -0.187 | 0.108 | 0.187 |
| 334_NA | -0.021 | 0.859 | -0.184 | 0.113 | 0.184 |
| 917_NA | 0.14 | 0.229 | -0.182 | 0.117 | 0.182 |
| 94_NA | -0.182 | 0.119 | -0.027 | 0.816 | 0.182 |
| 925_NA | -0.179 | 0.125 | 0.11 | 0.347 | 0.179 |
| 211_NA | -0.126 | 0.282 | -0.175 | 0.134 | 0.175 |
| 239_NA | -0.175 | 0.134 | 0.124 | 0.288 | 0.175 |
| 169_NA | 0.174 | 0.136 | -0.081 | 0.489 | 0.174 |
| 845_NA | -0.173 | 0.137 | -0.075 | 0.523 | 0.173 |

| Feature | PCoA Axis 1 (r) | p-value _PCoA1 | PCoA Axis 2 (r) | p-value _PCoA2 | max_abs _cor |
|---|---|---|---|---|---|
| 687_NA | 0.172 | 0.14 | -0.12 | 0.304 | 0.172 |
| 526_Glucose-1-phosphate | 0.141 | 0.228 | 0.163 | 0.161 | 0.163 |
| 390_NA | 0.162 | 0.165 | -0.064 | 0.583 | 0.162 |
| 519_NA | -0.155 | 0.183 | -0.158 | 0.175 | 0.158 |
| 697_NA | -0.109 | 0.353 | 0.157 | 0.178 | 0.157 |
| 589_NA | -0.03 | 0.795 | 0.142 | 0.224 | 0.142 |
| 904_Butyl octacosyl ether | 0.037 | 0.753 | 0.122 | 0.297 | 0.122 |
| 463_NA | -0.117 | 0.318 | 0.073 | 0.533 | 0.117 |
| 622_NA | 0.003 | 0.978 | 0.103 | 0.38 | 0.103 |
| 879_NA | -0.094 | 0.421 | 0.063 | 0.592 | 0.094 |
| 631_NA | -0.074 | 0.525 | -0.018 | 0.875 | 0.074 |
| 887_NA | -0.022 | 0.853 | 0.053 | 0.651 | 0.053 |
| 921_NA | -0.045 | 0.702 | 0.052 | 0.656 | 0.052 |
| 418_NA | -0.002 | 0.986 | -0.051 | 0.666 | 0.051 |
| 681_NA | 0.018 | 0.88 | 0.037 | 0.755 | 0.037 |
| 948_NA | 0.015 | 0.895 | 0.031 | 0.791 | 0.031 |
| 410_NA | 0.031 | 0.794 | -0.026 | 0.821 | 0.031 |

[a]Correlation coefficients (r) represent the association between metabolite intensities and the first two PCoA axes. p-values were calculated using Spearman correlation tests. max_abs_cor represents the maximum absolute correlation coefficient between metabolite intensities and the first two PCoA axes.

**Table S5. Classification Accuracy and Balanced Accuracy for Each Fold during Repeated 10-Fold Cross-validation of the Random Forest Model**

| Repeat | Fold | Accuracy | Balanced Accuracy |
|---|---|---|---|
| 1 | 1 | 1 | 1 |
| 1 | 2 | 1 | 1 |
| 1 | 3 | 1 | 1 |
| 1 | 4 | 1 | 1 |
| 1 | 5 | 1 | 1 |
| 1 | 6 | 1 | 1 |
| 1 | 7 | 1 | 1 |
| 1 | 8 | 1 | 1 |
| 1 | 9 | 1 | 1 |
| 1 | 10 | 1 | 1 |
| 2 | 1 | 1 | 1 |
| 2 | 2 | 1 | 1 |
| 2 | 3 | 1 | 1 |
| 2 | 4 | 1 | 1 |
| 2 | 5 | 1 | 1 |
| 2 | 6 | 1 | 1 |
| 2 | 7 | 1 | 1 |
| 2 | 8 | 1 | 1 |
| 2 | 9 | 1 | 1 |
| 2 | 10 | 1 | 1 |
| 3 | 1 | 1 | 1 |
| 3 | 2 | 1 | 1 |
| 3 | 3 | 1 | 1 |
| 3 | 4 | 1 | 1 |
| 3 | 5 | 1 | 1 |
| 3 | 6 | 1 | 1 |
| 3 | 7 | 1 | 1 |
| 3 | 8 | 1 | 1 |
| 3 | 9 | 1 | 1 |
| 3 | 10 | 1 | 1 |
| 4 | 1 | 1 | 1 |
| 4 | 2 | 1 | 1 |
| 4 | 3 | 1 | 1 |
| 4 | 4 | 1 | 1 |
| 4 | 5 | 1 | 1 |
| 4 | 6 | 1 | 1 |

| Repeat | Fold | Accuracy | Balanced Accuracy |
|---|---|---|---|
| 4 | 7 | 1 | 1 |
| 4 | 8 | 1 | 1 |
| 4 | 9 | 1 | 1 |
| 4 | 10 | 1 | 1 |
| 5 | 1 | 1 | 1 |
| 5 | 2 | 1 | 1 |
| 5 | 3 | 1 | 1 |
| 5 | 4 | 1 | 1 |
| 5 | 5 | 1 | 1 |
| 5 | 6 | 1 | 1 |
| 5 | 7 | 1 | 1 |
| 5 | 8 | 1 | 1 |
| 5 | 9 | 1 | 1 |
| 5 | 10 | 1 | 1 |
| 6 | 1 | 1 | 1 |
| 6 | 2 | 1 | 1 |
| 6 | 3 | 1 | 1 |
| 6 | 4 | 1 | 1 |
| 6 | 5 | 1 | 1 |
| 6 | 6 | 1 | 1 |
| 6 | 7 | 1 | 1 |
| 6 | 8 | 1 | 1 |
| 6 | 9 | 1 | 1 |
| 6 | 10 | 1 | 1 |
| 7 | 1 | 1 | 1 |
| 7 | 2 | 1 | 1 |
| 7 | 3 | 1 | 1 |
| 7 | 4 | 1 | 1 |
| 7 | 5 | 1 | 1 |
| 7 | 6 | 1 | 1 |
| 7 | 7 | 1 | 1 |
| 7 | 8 | 1 | 1 |
| 7 | 9 | 1 | 1 |
| 7 | 10 | 1 | 1 |
| 8 | 1 | 1 | 1 |
| 8 | 2 | 1 | 1 |
| 8 | 3 | 1 | 1 |
| 8 | 4 | 1 | 1 |
| 8 | 5 | 1 | 1 |

| Repeat | Fold | Accuracy | Balanced Accuracy |
|---|---|---|---|
| 8 | 6 | 1 | 1 |
| 8 | 7 | 1 | 1 |
| 8 | 8 | 1 | 1 |
| 8 | 9 | 1 | 1 |
| 8 | 10 | 1 | 1 |
| 9 | 1 | 1 | 1 |
| 9 | 2 | 1 | 1 |
| 9 | 3 | 1 | 1 |
| 9 | 4 | 1 | 1 |
| 9 | 5 | 1 | 1 |
| 9 | 6 | 1 | 1 |
| 9 | 7 | 1 | 1 |
| 9 | 8 | 1 | 1 |
| 9 | 9 | 1 | 1 |
| 9 | 10 | 1 | 1 |
| 10 | 1 | 1 | 1 |
| 10 | 2 | 1 | 1 |
| 10 | 3 | 1 | 1 |
| 10 | 4 | 1 | 1 |
| 10 | 5 | 1 | 1 |
| 10 | 6 | 1 | 1 |
| 10 | 7 | 1 | 1 |
| 10 | 8 | 1 | 1 |
| 10 | 9 | 1 | 1 |
| 10 | 10 | 1 | 1 |
| 11 | 1 | 1 | 1 |
| 11 | 2 | 1 | 1 |
| 11 | 3 | 1 | 1 |
| 11 | 4 | 1 | 1 |
| 11 | 5 | 1 | 1 |
| 11 | 6 | 1 | 1 |
| 11 | 7 | 1 | 1 |
| 11 | 8 | 1 | 1 |
| 11 | 9 | 1 | 1 |
| 11 | 10 | 1 | 1 |
| 12 | 1 | 1 | 1 |
| 12 | 2 | 1 | 1 |
| 12 | 3 | 1 | 1 |
| 12 | 4 | 1 | 1 |

| Repeat | Fold | Accuracy | Balanced Accuracy |
|---|---|---|---|
| 12 | 5 | 1 | 1 |
| 12 | 6 | 1 | 1 |
| 12 | 7 | 1 | 1 |
| 12 | 8 | 1 | 1 |
| 12 | 9 | 1 | 1 |
| 12 | 10 | 1 | 1 |
| 13 | 1 | 1 | 1 |
| 13 | 2 | 1 | 1 |
| 13 | 3 | 1 | 1 |
| 13 | 4 | 1 | 1 |
| 13 | 5 | 1 | 1 |
| 13 | 6 | 1 | 1 |
| 13 | 7 | 1 | 1 |
| 13 | 8 | 1 | 1 |
| 13 | 9 | 1 | 1 |
| 13 | 10 | 1 | 1 |
| 14 | 1 | 1 | 1 |
| 14 | 2 | 1 | 1 |
| 14 | 3 | 1 | 1 |
| 14 | 4 | 1 | 1 |
| 14 | 5 | 1 | 1 |
| 14 | 6 | 1 | 1 |
| 14 | 7 | 1 | 1 |
| 14 | 8 | 1 | 1 |
| 14 | 9 | 1 | 1 |
| 14 | 10 | 1 | 1 |
| 15 | 1 | 1 | 1 |
| 15 | 2 | 1 | 1 |
| 15 | 3 | 1 | 1 |
| 15 | 4 | 1 | 1 |
| 15 | 5 | 1 | 1 |
| 15 | 6 | 1 | 1 |
| 15 | 7 | 1 | 1 |
| 15 | 8 | 1 | 1 |
| 15 | 9 | 1 | 1 |
| 15 | 10 | 1 | 1 |
| 16 | 1 | 1 | 1 |
| 16 | 2 | 1 | 1 |
| 16 | 3 | 1 | 1 |

| Repeat | Fold | Accuracy | Balanced Accuracy |
|---|---|---|---|
| 16 | 4 | 1 | 1 |
| 16 | 5 | 1 | 1 |
| 16 | 6 | 1 | 1 |
| 16 | 7 | 1 | 1 |
| 16 | 8 | 1 | 1 |
| 16 | 9 | 1 | 1 |
| 16 | 10 | 1 | 1 |
| 17 | 1 | 1 | 1 |
| 17 | 2 | 1 | 1 |
| 17 | 3 | 1 | 1 |
| 17 | 4 | 1 | 1 |
| 17 | 5 | 1 | 1 |
| 17 | 6 | 1 | 1 |
| 17 | 7 | 1 | 1 |
| 17 | 8 | 1 | 1 |
| 17 | 9 | 1 | 1 |
| 17 | 10 | 1 | 1 |
| 18 | 1 | 1 | 1 |
| 18 | 2 | 1 | 1 |
| 18 | 3 | 1 | 1 |
| 18 | 4 | 1 | 1 |
| 18 | 5 | 1 | 1 |
| 18 | 6 | 1 | 1 |
| 18 | 7 | 1 | 1 |
| 18 | 8 | 1 | 1 |
| 18 | 9 | 1 | 1 |
| 18 | 10 | 1 | 1 |
| 19 | 1 | 1 | 1 |
| 19 | 2 | 1 | 1 |
| 19 | 3 | 1 | 1 |
| 19 | 4 | 1 | 1 |
| 19 | 5 | 1 | 1 |
| 19 | 6 | 1 | 1 |
| 19 | 7 | 1 | 1 |
| 19 | 8 | 1 | 1 |
| 19 | 9 | 1 | 1 |
| 19 | 10 | 1 | 1 |
| 20 | 1 | 1 | 1 |
| 20 | 2 | 1 | 1 |

| Repeat | Fold | Accuracy | Balanced Accuracy |
|---|---|---|---|
| 20 | 3 | 1 | 1 |
| 20 | 4 | 1 | 1 |
| 20 | 5 | 1 | 1 |
| 20 | 6 | 1 | 1 |
| 20 | 7 | 1 | 1 |
| 20 | 8 | 1 | 1 |
| 20 | 9 | 1 | 1 |
| 20 | 10 | 1 | 1 |

**Table S6. Pooled Confusion Matrix Summarizing Random Forest Classification Results across all Repeated Cross-validation Runs**

| Actual Class | Predicted Class | Number of Sample |
|---|---|---|
| Bagasse | Bagasse | 300 |
| Filter cake | Bagasse | 0 |
| Stems | Bagasse | 0 |
| Tops | Bagasse | 0 |
| Trash | Bagasse | 0 |
| Bagasse | Filter cake | 0 |
| Filter cake | Filter cake | 300 |
| Stems | Filter cake | 0 |
| Tops | Filter cake | 0 |
| Trash | Filter cake | 0 |
| Bagasse | Stems | 0 |
| Filter cake | Stems | 0 |
| Stems | Stems | 300 |
| Tops | Stems | 0 |
| Trash | Stems | 0 |
| Bagasse | Tops | 0 |
| Filter cake | Tops | 0 |
| Stems | Tops | 0 |
| Tops | Tops | 300 |
| Trash | Tops | 0 |
| Bagasse | Trash | 0 |
| Filter cake | Trash | 0 |
| Stems | Trash | 0 |
| Tops | Trash | 0 |
| Trash | Trash | 300 |

# Table S7. Metabolite Importance Scores Based on Random Forest Classification

| Cluster index | Putative Compound | Bagasse | Bagasse *p*-Value | Filter cake | Filter cake *p*-Value | Stems | Stems *p*-Value | Tops | Tops *p*-Value | Trash | Trash *p*-Value | Mean Decrease Accuracy | Mean Decrease Accuracy *p*-Value | Mean Decrease Gini | Mean Decrease Gini *p*-Value |
|---|---|---|---|---|---|---|---|---|---|---|---|---|---|---|---|
| 601 | NA | 3.93 | 0.00 | 3.41 | 0.00 | 3.14 | 0.00 | 3.61 | 0.01 | 5.14 | 0.00 | 5.13 | 0.00 | 0.67 | 0.00 |
| 461 | NA | 4.23 | 0.00 | 3.89 | 0.00 | 3.49 | 0.00 | 3.87 | 0.00 | 4.67 | 0.00 | 5.05 | 0.00 | 0.74 | 0.00 |
| 901 | 1-Tetratriacontanol | 4.58 | 0.00 | 4.22 | 0.00 | 3.86 | 0.00 | 3.75 | 0.00 | 4.65 | 0.00 | 4.98 | 0.00 | 0.73 | 0.00 |
| 319 | Hexadecanoic acid (Palmitic acid) | 4.24 | 0.00 | 4.13 | 0.00 | 2.88 | 0.00 | 3.28 | 0.00 | 4.58 | 0.00 | 4.60 | 0.00 | 0.64 | 0.00 |
| 392 | (9*Z*)-Octadecenoic acid (Oleic acid) | 3.57 | 0.00 | 3.38 | 0.00 | 4.33 | 0.00 | 2.96 | 0.00 | 3.63 | 0.00 | 4.53 | 0.00 | 0.53 | 0.00 |
| 558 | NA | 3.23 | 0.00 | 4.63 | 0.00 | 2.92 | 0.00 | 2.43 | 0.00 | 2.75 | 0.00 | 4.52 | 0.00 | 0.51 | 0.00 |
| 792 | NA | 3.02 | 0.00 | 2.53 | 0.00 | 2.55 | 0.00 | 4.22 | 0.00 | 2.47 | 0.00 | 4.38 | 0.00 | 0.53 | 0.00 |
| 795 | Glutinol | 3.32 | 0.00 | 1.86 | 0.02 | 3.71 | 0.00 | 2.86 | 0.00 | 3.09 | 0.00 | 4.37 | 0.00 | 0.56 | 0.00 |
| 542 | NA | 3.83 | 0.00 | 3.14 | 0.00 | 2.93 | 0.00 | 4.37 | 0.00 | 3.27 | 0.00 | 4.31 | 0.00 | 0.51 | 0.00 |
| 614 | 1-Hexacosanol | 3.65 | 0.00 | 4.30 | 0.00 | 3.17 | 0.00 | 2.73 | 0.00 | 3.69 | 0.00 | 4.25 | 0.00 | 0.50 | 0.00 |
| 753 | NA | 2.71 | 0.01 | 2.86 | 0.00 | 2.62 | 0.00 | 3.80 | 0.00 | 2.10 | 0.01 | 4.20 | 0.00 | 0.48 | 0.00 |
| 334 | NA | 2.83 | 0.01 | 2.54 | 0.00 | 4.20 | 0.00 | 2.89 | 0.00 | 2.62 | 0.00 | 4.15 | 0.00 | 0.45 | 0.00 |
| 907 | Nonadecanenitrile | 3.86 | 0.00 | 3.76 | 0.00 | 2.59 | 0.00 | 2.45 | 0.01 | 3.58 | 0.00 | 4.08 | 0.00 | 0.44 | 0.00 |
| 391 | (9*Z*)-Octadecenoic acid (Oleic acid) | 4.03 | 0.00 | 2.67 | 0.00 | 2.67 | 0.00 | 1.99 | 0.01 | 2.49 | 0.00 | 3.97 | 0.00 | 0.42 | 0.00 |
| 330 | NA | 3.30 | 0.00 | 2.60 | 0.00 | 4.01 | 0.00 | 3.00 | 0.00 | 3.06 | 0.00 | 3.95 | 0.00 | 0.40 | 0.00 |
| 365 | 2-Methylpentadecanoic acid | 2.68 | 0.00 | 2.62 | 0.00 | 2.31 | 0.01 | 3.95 | 0.00 | 3.09 | 0.00 | 3.91 | 0.00 | 0.40 | 0.00 |
| 385 | NA | 4.07 | 0.00 | 3.34 | 0.00 | 3.05 | 0.01 | 1.89 | 0.01 | 2.70 | 0.00 | 3.90 | 0.00 | 0.44 | 0.00 |
| 665 | NA | 3.43 | 0.00 | 2.81 | 0.00 | 2.39 | 0.00 | 3.21 | 0.00 | 3.76 | 0.00 | 3.89 | 0.00 | 0.45 | 0.00 |
| 798 | Lanosterol | 3.19 | 0.00 | 2.25 | 0.00 | 3.51 | 0.00 | 2.28 | 0.00 | 3.16 | 0.00 | 3.85 | 0.00 | 0.39 | 0.00 |
| 269 | NA | 3.52 | 0.00 | 2.78 | 0.00 | 2.32 | 0.00 | 3.59 | 0.00 | 2.92 | 0.00 | 3.82 | 0.00 | 0.40 | 0.00 |
| 194 | NA | 2.95 | 0.00 | 3.82 | 0.00 | 1.89 | 0.01 | 2.56 | 0.00 | 2.95 | 0.00 | 3.81 | 0.00 | 0.39 | 0.00 |
| 759 | NA | 3.31 | 0.00 | 2.29 | 0.00 | 2.85 | 0.00 | 3.90 | 0.00 | 3.15 | 0.00 | 3.79 | 0.00 | 0.40 | 0.00 |
| 679 | Cholesterol | 2.36 | 0.00 | 2.81 | 0.00 | 3.80 | 0.00 | 2.46 | 0.00 | 2.34 | 0.00 | 3.76 | 0.00 | 0.40 | 0.00 |
| 505 | NA | 2.47 | 0.00 | 3.26 | 0.00 | 2.96 | 0.00 | 2.21 | 0.01 | 3.18 | 0.00 | 3.73 | 0.00 | 0.39 | 0.00 |
| 695 | NA | 2.69 | 0.00 | 3.01 | 0.01 | 3.00 | 0.00 | 2.75 | 0.00 | 3.11 | 0.00 | 3.71 | 0.00 | 0.40 | 0.00 |
| 393 | (9*Z*)-Octadecenoic acid (Oleic acid) | 2.70 | 0.00 | 3.65 | 0.00 | 1.93 | 0.01 | 2.34 | 0.00 | 2.69 | 0.00 | 3.65 | 0.00 | 0.35 | 0.00 |
| 376 | NA | 3.05 | 0.00 | 2.87 | 0.01 | 3.57 | 0.00 | 2.50 | 0.00 | 2.32 | 0.01 | 3.61 | 0.00 | 0.37 | 0.00 |
| 728 | NA | 3.14 | 0.00 | 1.28 | 0.11 | 1.98 | 0.01 | 2.53 | 0.00 | 3.36 | 0.00 | 3.61 | 0.00 | 0.32 | 0.00 |
| 386 | Ethyl palmitate | 2.38 | 0.01 | 1.53 | 0.06 | 3.51 | 0.00 | 2.71 | 0.00 | 2.25 | 0.00 | 3.59 | 0.00 | 0.36 | 0.00 |
| 293 | Pentadecanoic acid (Pentadecylic acid) | 2.01 | 0.00 | 2.31 | 0.00 | 2.12 | 0.01 | 3.59 | 0.00 | 2.68 | 0.00 | 3.49 | 0.00 | 0.33 | 0.00 |
| 378 | Phytol | 1.86 | 0.01 | 2.39 | 0.01 | 2.51 | 0.00 | 3.49 | 0.00 | 2.78 | 0.00 | 3.48 | 0.00 | 0.32 | 0.00 |
| 770 | NA | 2.47 | 0.01 | 1.15 | 0.14 | 2.49 | 0.00 | 1.61 | 0.06 | 3.41 | 0.00 | 3.47 | 0.00 | 0.31 | 0.00 |
| 173 | 2-Deoxyarabitol | 2.81 | 0.00 | 2.55 | 0.00 | 2.23 | 0.01 | 2.09 | 0.01 | 3.10 | 0.00 | 3.44 | 0.00 | 0.32 | 0.00 |
| 549 | 2-Oleoylglycerol (2-monoolein) | 2.50 | 0.00 | 2.27 | 0.01 | 3.42 | 0.00 | 2.46 | 0.01 | 2.48 | 0.00 | 3.37 | 0.00 | 0.31 | 0.01 |
| 514 | NA | 2.82 | 0.00 | 2.15 | 0.01 | 1.74 | 0.01 | 2.70 | 0.00 | 2.21 | 0.00 | 3.36 | 0.00 | 0.30 | 0.00 |
| 521 | NA | 3.00 | 0.00 | 2.07 | 0.01 | 1.74 | 0.01 | 3.04 | 0.00 | 2.47 | 0.00 | 3.36 | 0.00 | 0.34 | 0.00 |
| 513 | NA | 2.62 | 0.00 | 1.35 | 0.12 | 2.34 | 0.01 | 1.42 | 0.07 | 3.03 | 0.00 | 3.27 | 0.00 | 0.31 | 0.00 |
| 394 | (9*Z*)-Octadecenoic acid (Oleic acid) | 2.83 | 0.00 | -1.49 | 0.95 | 3.10 | 0.00 | 2.14 | 0.01 | 2.34 | 0.01 | 3.26 | 0.01 | 0.31 | 0.01 |
| 283 | NA | 2.39 | 0.00 | 2.30 | 0.00 | 2.33 | 0.01 | 3.25 | 0.00 | 2.11 | 0.02 | 3.25 | 0.00 | 0.29 | 0.00 |
| 818 | NA | 2.95 | 0.00 | 3.15 | 0.00 | 1.35 | 0.09 | 2.45 | 0.00 | 2.34 | 0.01 | 3.25 | 0.00 | 0.28 | 0.00 |
| 158 | Threonic acid | 2.92 | 0.00 | 3.27 | 0.00 | -1.00 | 0.89 | 1.59 | 0.06 | 2.27 | 0.00 | 3.24 | 0.00 | 0.29 | 0.00 |
| 420 | NA | 2.17 | 0.01 | 2.57 | 0.00 | 2.35 | 0.01 | 3.12 | 0.00 | 2.19 | 0.01 | 3.21 | 0.00 | 0.28 | 0.00 |
| 227 | NA | 3.01 | 0.00 | 2.53 | 0.00 | 1.79 | 0.01 | 2.25 | 0.00 | 1.89 | 0.01 | 3.20 | 0.00 | 0.28 | 0.00 |
| 560 | 1-Oleoylglycerol (1-monoolein) | 2.48 | 0.00 | 2.73 | 0.00 | 3.19 | 0.00 | 2.31 | 0.00 | 2.13 | 0.02 | 3.19 | 0.00 | 0.27 | 0.00 |
| 883 | NA | 2.90 | 0.00 | 0.19 | 0.38 | 1.64 | 0.05 | 2.75 | 0.00 | 2.50 | 0.00 | 3.09 | 0.00 | 0.30 | 0.00 |
| 681 | NA | 2.66 | 0.00 | 2.23 | 0.01 | 2.47 | 0.00 | 2.16 | 0.01 | 3.08 | 0.01 | 3.07 | 0.01 | 0.26 | 0.01 |
| 218 | NA | 2.46 | 0.00 | 2.21 | 0.01 | 0.00 | 0.53 | 1.58 | 0.06 | 2.82 | 0.00 | 2.98 | 0.00 | 0.25 | 0.01 |

| Cluster index | Putative Compound | Bagasse | Bagasse *p*-Value | Filter cake | Filter cake *p*-Value | Stems | Stems *p*-Value | Tops | Tops *p*-Value | Trash | Trash *p*-Value | Mean Decrease Accuracy | Mean Decrease Accuracy *p*-Value | Mean Decrease Gini | Mean Decrease Gini *p*-Value |
|---|---|---|---|---|---|---|---|---|---|---|---|---|---|---|---|
| 361 | Heptadecanoic acid (Margaric acid) | 1.89 | 0.01 | 2.75 | 0.00 | 2.72 | 0.00 | 2.22 | 0.00 | 2.71 | 0.00 | 2.97 | 0.00 | 0.26 | 0.00 |
| 922 | NA | 2.30 | 0.01 | 0.72 | 0.25 | 1.46 | 0.04 | 2.42 | 0.01 | 2.99 | 0.00 | 2.95 | 0.00 | 0.24 | 0.01 |
| 799 | NA | 1.87 | 0.03 | 2.88 | 0.00 | 2.37 | 0.00 | 1.26 | 0.16 | 2.18 | 0.01 | 2.84 | 0.01 | 0.25 | 0.00 |
| 817 | 1-Dotriacontanol | 1.87 | 0.01 | 1.82 | 0.02 | 2.72 | 0.00 | 2.11 | 0.00 | 1.88 | 0.01 | 2.79 | 0.00 | 0.22 | 0.00 |
| 824 | NA | 2.21 | 0.00 | 2.61 | 0.00 | 0.52 | 0.33 | 2.00 | 0.01 | 2.57 | 0.01 | 2.77 | 0.00 | 0.27 | 0.00 |
| 740 | 1-Triacontanol | 1.86 | 0.01 | 1.92 | 0.01 | 2.80 | 0.00 | 1.54 | 0.04 | 2.38 | 0.00 | 2.72 | 0.00 | 0.21 | 0.00 |
| 945 | NA | 2.14 | 0.01 | 0.95 | 0.27 | 2.63 | 0.01 | 1.41 | 0.11 | 2.06 | 0.01 | 2.72 | 0.00 | 0.17 | 0.07 |
| 502 | NA | 2.47 | 0.00 | 1.89 | 0.02 | 1.98 | 0.02 | 1.41 | 0.13 | 2.51 | 0.00 | 2.70 | 0.01 | 0.20 | 0.01 |
| 849 | NA | 1.27 | 0.10 | 2.56 | 0.00 | 1.87 | 0.00 | 2.14 | 0.00 | 2.09 | 0.01 | 2.70 | 0.00 | 0.19 | 0.00 |
| 468 | NA | 2.27 | 0.01 | 1.74 | 0.03 | 1.64 | 0.04 | 2.61 | 0.00 | 1.22 | 0.14 | 2.67 | 0.00 | 0.20 | 0.01 |
| 478 | Eicosanoic acid (Arachidic acid) | 2.25 | 0.00 | 2.10 | 0.01 | 0.00 | 0.61 | 1.53 | 0.05 | 2.39 | 0.00 | 2.62 | 0.00 | 0.20 | 0.01 |
| 853 | NA | 1.29 | 0.12 | 1.95 | 0.03 | 1.99 | 0.03 | 2.53 | 0.01 | 1.86 | 0.04 | 2.50 | 0.01 | 0.20 | 0.04 |
| 755 | NA | 2.16 | 0.01 | -0.27 | 0.61 | 2.36 | 0.01 | 1.30 | 0.12 | 1.90 | 0.02 | 2.50 | 0.00 | 0.18 | 0.03 |
| 469 | NA | 0.00 | 0.59 | 1.87 | 0.02 | 1.64 | 0.03 | 1.30 | 0.09 | 2.40 | 0.01 | 2.49 | 0.00 | 0.14 | 0.04 |
| 234 | Ribonic acid | 1.94 | 0.01 | 2.39 | 0.01 | 1.41 | 0.09 | 1.23 | 0.08 | 2.04 | 0.00 | 2.36 | 0.00 | 0.15 | 0.01 |
| 285 | NA | 1.47 | 0.05 | 1.73 | 0.05 | 1.35 | 0.15 | 1.39 | 0.10 | 1.53 | 0.04 | 2.36 | 0.02 | 0.10 | 0.44 |
| 496 | NA | 1.57 | 0.05 | 1.30 | 0.09 | 2.41 | 0.00 | 1.39 | 0.13 | 2.20 | 0.01 | 2.35 | 0.01 | 0.20 | 0.02 |
| 923 | NA | 1.00 | 0.20 | 1.39 | 0.09 | 0.00 | 0.50 | 2.39 | 0.01 | 2.05 | 0.01 | 2.35 | 0.01 | 0.16 | 0.03 |
| 713 | Campesterol | 1.01 | 0.09 | 2.37 | 0.00 | 1.74 | 0.01 | 1.61 | 0.02 | 2.01 | 0.00 | 2.34 | 0.01 | 0.15 | 0.00 |
| 903 | Isobutyl tetratriacontyl ether | 1.53 | 0.05 | 1.85 | 0.04 | 0.00 | 0.50 | 1.66 | 0.05 | 1.64 | 0.04 | 2.32 | 0.02 | 0.14 | 0.10 |
| 694 | NA | 0.42 | 0.35 | 1.23 | 0.15 | 1.59 | 0.05 | 2.12 | 0.01 | 1.26 | 0.11 | 2.29 | 0.01 | 0.14 | 0.05 |
| 729 | Tritriacontane | 1.60 | 0.04 | 0.00 | 0.63 | 1.83 | 0.01 | 1.73 | 0.01 | 1.61 | 0.04 | 2.29 | 0.00 | 0.15 | 0.03 |
| 884 | NA | 1.64 | 0.04 | -1.00 | 0.82 | 0.00 | 0.52 | 2.34 | 0.01 | 1.42 | 0.06 | 2.24 | 0.01 | 0.14 | 0.25 |
| 648 | 1,2-Epoxyoctadecane | 1.96 | 0.01 | 1.00 | 0.26 | 2.00 | 0.01 | 0.00 | 0.53 | 1.00 | 0.25 | 2.18 | 0.02 | 0.11 | 0.45 |
| 677 | NA | 1.56 | 0.03 | 2.15 | 0.01 | 1.00 | 0.22 | 1.00 | 0.22 | 0.00 | 0.55 | 2.18 | 0.02 | 0.09 | 0.43 |
| 109 | 2,3-Dihydroxypropanoic acid (Glyceric acid) | 2.16 | 0.00 | 2.26 | 0.01 | 0.00 | 0.60 | 1.00 | 0.25 | 1.00 | 0.21 | 2.17 | 0.01 | 0.15 | 0.00 |
| 379 | NA | 1.97 | 0.02 | 1.00 | 0.19 | 2.23 | 0.00 | -1.00 | 0.83 | 2.01 | 0.01 | 2.16 | 0.01 | 0.13 | 0.28 |
| 703 | NA | 1.00 | 0.25 | 1.99 | 0.01 | 1.96 | 0.01 | 1.00 | 0.26 | 0.59 | 0.25 | 2.16 | 0.01 | 0.12 | 0.26 |
| 609 | NA | 1.42 | 0.09 | 1.57 | 0.07 | 0.00 | 0.52 | 2.16 | 0.01 | 1.93 | 0.02 | 2.16 | 0.02 | 0.13 | 0.18 |
| 676 | 1-Octacosanol | 1.00 | 0.24 | 2.15 | 0.00 | 1.73 | 0.01 | 1.32 | 0.10 | 1.95 | 0.01 | 2.15 | 0.00 | 0.13 | 0.02 |
| 119 | NA | 1.87 | 0.04 | 1.00 | 0.24 | 1.40 | 0.09 | 1.40 | 0.13 | 1.66 | 0.06 | 2.15 | 0.03 | 0.11 | 0.44 |
| 220 | NA | 1.91 | 0.01 | 1.37 | 0.11 | 1.87 | 0.01 | 1.37 | 0.08 | 1.52 | 0.04 | 2.14 | 0.00 | 0.13 | 0.09 |
| 437 | NA | 1.00 | 0.25 | 1.97 | 0.01 | 1.00 | 0.26 | 1.24 | 0.13 | 2.08 | 0.00 | 2.12 | 0.01 | 0.12 | 0.05 |
| 689 | NA | 2.14 | 0.02 | 1.29 | 0.14 | 0.00 | 0.52 | 1.40 | 0.11 | 1.00 | 0.25 | 2.11 | 0.02 | 0.13 | 0.33 |
| 297 | *p*-Coumaric acid | 1.85 | 0.03 | 1.41 | 0.10 | 0.42 | 0.29 | 1.33 | 0.10 | 1.97 | 0.01 | 2.09 | 0.02 | 0.11 | 0.12 |
| 531 | NA | 2.00 | 0.01 | 1.42 | 0.05 | 1.15 | 0.09 | 1.90 | 0.01 | 1.70 | 0.04 | 2.09 | 0.01 | 0.10 | 0.37 |
| 318 | 4-Hydroxy-3-methoxymandelic acid | 1.88 | 0.02 | 1.65 | 0.04 | 1.41 | 0.11 | 1.54 | 0.05 | 1.67 | 0.03 | 2.07 | 0.01 | 0.12 | 0.06 |
| 317 | Gluconic acid | 1.91 | 0.01 | 2.11 | 0.00 | 0.00 | 0.62 | 1.35 | 0.11 | 1.00 | 0.26 | 2.07 | 0.01 | 0.10 | 0.09 |
| 599 | NA | 1.57 | 0.04 | 1.00 | 0.26 | 0.00 | 0.59 | 1.65 | 0.03 | 1.74 | 0.04 | 2.06 | 0.02 | 0.11 | 0.30 |
| 296 | NA | 1.08 | 0.16 | 1.95 | 0.02 | 1.97 | 0.03 | 0.00 | 0.54 | 1.74 | 0.04 | 2.05 | 0.04 | 0.11 | 0.37 |
| 667 | Hentriacontane | 2.36 | 0.00 | -1.00 | 0.91 | 1.00 | 0.24 | 1.79 | 0.02 | 1.27 | 0.11 | 2.05 | 0.02 | 0.14 | 0.02 |
| 451 | NA | 1.89 | 0.01 | 1.90 | 0.01 | 1.32 | 0.11 | 1.41 | 0.10 | 1.90 | 0.01 | 2.03 | 0.03 | 0.12 | 0.13 |
| 284 | NA | 1.00 | 0.28 | 1.00 | 0.26 | 1.35 | 0.08 | 1.00 | 0.27 | 1.88 | 0.02 | 1.99 | 0.03 | 0.08 | 0.61 |
| 483 | NA | 1.00 | 0.24 | 0.16 | 0.42 | 1.97 | 0.01 | 1.00 | 0.34 | 2.00 | 0.01 | 1.97 | 0.03 | 0.12 | 0.15 |
| 196 | NA | 1.70 | 0.02 | 1.67 | 0.05 | 1.00 | 0.21 | 1.40 | 0.10 | 1.00 | 0.29 | 1.97 | 0.02 | 0.10 | 0.36 |
| 717 | NA | 0.00 | 0.63 | 2.02 | 0.01 | 1.00 | 0.26 | 0.00 | 0.62 | 1.58 | 0.03 | 1.97 | 0.03 | 0.13 | 0.02 |
| 908 | NA | 1.94 | 0.01 | 1.70 | 0.03 | 1.00 | 0.17 | 1.49 | 0.05 | 1.29 | 0.12 | 1.96 | 0.01 | 0.12 | 0.09 |
| 313 | NA | 0.85 | 0.30 | 1.95 | 0.01 | 1.00 | 0.27 | 1.00 | 0.26 | 1.00 | 0.25 | 1.94 | 0.03 | 0.10 | 0.24 |
| 500 | NA | 1.42 | 0.07 | 1.32 | 0.12 | 1.87 | 0.03 | 1.00 | 0.22 | 1.37 | 0.12 | 1.93 | 0.03 | 0.10 | 0.48 |
| 398 | NA | 1.37 | 0.08 | 2.01 | 0.01 | 1.66 | 0.05 | 0.00 | 0.62 | 1.58 | 0.06 | 1.86 | 0.04 | 0.09 | 0.51 |

| **Cluster index** | **Putative Compound** | **Bagasse** | **Bagasse *p*-Value** | **Filter cake** | **Filter cake *p*-Value** | **Stems** | **Stems *p*-Value** | **Tops** | **Tops *p*-Value** | **Trash** | **Trash *p*-Value** | **Mean Decrease Accuracy** | **Mean Decrease Accuracy *p*-Value** | **Mean Decrease Gini** | **Mean Decrease Gini *p*-Value** |
|---|---|---|---|---|---|---|---|---|---|---|---|---|---|---|---|
| 802 | NA | 1.51 | 0.05 | 1.35 | 0.09 | 0.00 | 0.58 | 1.71 | 0.04 | 0.00 | 0.53 | 1.84 | 0.04 | 0.10 | 0.40 |
| 921 | NA | 1.00 | 0.19 | 1.74 | 0.03 | 1.42 | 0.07 | 1.00 | 0.25 | 1.00 | 0.24 | 1.79 | 0.04 | 0.07 | 0.86 |
| 58 | 3-Hydroxybutanoic acid ($\beta$-Hydroxybutyric acid,) | 1.00 | 0.25 | 1.00 | 0.21 | 1.00 | 0.21 | 1.42 | 0.05 | 1.00 | 0.27 | 1.78 | 0.02 | 0.06 | 0.69 |
| 217 | Arabitol | 1.90 | 0.02 | 0.00 | 0.54 | -1.00 | 0.84 | 0.00 | 0.52 | 1.00 | 0.23 | 1.74 | 0.04 | 0.11 | 0.47 |
| 850 | NA | 1.60 | 0.07 | 1.42 | 0.07 | 0.00 | 0.59 | 1.73 | 0.03 | 1.55 | 0.02 | 1.74 | 0.03 | 0.06 | 0.79 |
| 298 | NA | 1.37 | 0.10 | 0.00 | 0.54 | 1.40 | 0.09 | 0.00 | 0.57 | 1.00 | 0.28 | 1.74 | 0.04 | 0.07 | 0.64 |
| 546 | NA | 1.30 | 0.09 | 1.69 | 0.04 | 0.00 | 0.55 | 0.00 | 0.51 | 1.18 | 0.13 | 1.74 | 0.04 | 0.09 | 0.60 |
| 690 | NA | 1.00 | 0.18 | 1.00 | 0.21 | 1.66 | 0.05 | 0.00 | 0.53 | 1.72 | 0.04 | 1.74 | 0.05 | 0.08 | 0.77 |
| 228 | Aconitic acid | -0.30 | 0.61 | 1.58 | 0.05 | 1.41 | 0.09 | 1.00 | 0.21 | 1.25 | 0.13 | 1.73 | 0.04 | 0.11 | 0.24 |
| 305 | Galactose | 1.36 | 0.13 | 1.42 | 0.05 | 1.39 | 0.11 | 0.00 | 0.60 | 1.00 | 0.26 | 1.72 | 0.08 | 0.06 | 0.77 |
| 568 | NA | 1.00 | 0.25 | 1.27 | 0.11 | 0.00 | 0.59 | 1.39 | 0.10 | 1.00 | 0.23 | 1.72 | 0.04 | 0.06 | 0.71 |
| 329 | *Myo*-Inositol | 1.63 | 0.04 | 1.00 | 0.29 | 1.00 | 0.24 | 1.25 | 0.10 | -1.00 | 0.91 | 1.72 | 0.03 | 0.07 | 0.38 |
| 340 | NA | 1.27 | 0.13 | 1.00 | 0.29 | 1.00 | 0.27 | 1.00 | 0.24 | 1.00 | 0.24 | 1.72 | 0.07 | 0.03 | 0.99 |
| 270 | Tetradecanoic acid (Myristic acid) | 1.00 | 0.25 | 0.00 | 0.62 | 0.00 | 0.60 | 1.71 | 0.03 | 1.37 | 0.11 | 1.71 | 0.04 | 0.08 | 0.43 |
| 247 | *p*-Coumaric acid | 1.71 | 0.03 | 0.00 | 0.55 | 0.00 | 0.51 | 1.29 | 0.13 | 1.00 | 0.27 | 1.70 | 0.05 | 0.04 | 0.93 |
| 900 | Tritriacontanoic acid (Psyllic acid) | 1.18 | 0.13 | 1.00 | 0.23 | 0.00 | 0.54 | 1.25 | 0.09 | 1.69 | 0.04 | 1.70 | 0.04 | 0.08 | 0.62 |
| 83 | Urea | 1.42 | 0.12 | 1.00 | 0.29 | 1.00 | 0.26 | 1.00 | 0.26 | 1.00 | 0.19 | 1.67 | 0.07 | 0.05 | 0.87 |
| 739 | NA | 0.39 | 0.37 | 1.72 | 0.04 | 1.00 | 0.25 | 1.00 | 0.26 | 0.00 | 0.55 | 1.66 | 0.04 | 0.08 | 0.71 |
| 199 | NA | 1.00 | 0.27 | 1.00 | 0.24 | 1.39 | 0.11 | 0.00 | 0.55 | 1.20 | 0.15 | 1.66 | 0.05 | 0.07 | 0.73 |
| 876 | NA | 1.41 | 0.08 | 1.23 | 0.12 | 0.00 | 0.53 | 1.00 | 0.26 | 1.00 | 0.27 | 1.65 | 0.04 | 0.05 | 0.86 |
| 793 | NA | 1.24 | 0.16 | 0.00 | 0.58 | 1.60 | 0.04 | 1.00 | 0.28 | 0.00 | 0.59 | 1.65 | 0.08 | 0.06 | 0.66 |
| 403 | Octadecanoic acid (Stearic acid) | -0.39 | 0.63 | 1.00 | 0.33 | 1.53 | 0.05 | 0.00 | 0.58 | 1.59 | 0.04 | 1.64 | 0.08 | 0.08 | 0.42 |
| 229 | NA | 1.37 | 0.11 | 1.81 | 0.01 | -1.00 | 0.84 | 0.00 | 0.58 | 0.00 | 0.59 | 1.64 | 0.08 | 0.10 | 0.37 |
| 292 | Mannose | 0.00 | 0.67 | 1.66 | 0.02 | 0.00 | 0.63 | 1.00 | 0.26 | 1.00 | 0.24 | 1.63 | 0.04 | 0.05 | 0.59 |
| 735 | NA | 1.42 | 0.05 | 1.61 | 0.03 | 1.00 | 0.29 | 1.39 | 0.08 | -1.00 | 0.91 | 1.60 | 0.04 | 0.08 | 0.21 |
| 578 | NA | 1.39 | 0.11 | 1.00 | 0.23 | 1.00 | 0.27 | 1.00 | 0.26 | 1.18 | 0.11 | 1.57 | 0.06 | 0.07 | 0.66 |
| 764 | NA | 0.59 | 0.34 | -1.00 | 0.86 | 1.36 | 0.10 | 1.00 | 0.24 | 0.00 | 0.59 | 1.56 | 0.08 | 0.07 | 0.61 |
| 362 | NA | 1.36 | 0.09 | 0.00 | 0.58 | 0.00 | 0.60 | 1.41 | 0.09 | 1.00 | 0.26 | 1.55 | 0.06 | 0.06 | 0.62 |
| 608 | Nonacosane | -0.34 | 0.63 | 1.42 | 0.04 | 1.00 | 0.26 | 0.00 | 0.56 | 0.00 | 0.58 | 1.55 | 0.05 | 0.06 | 0.65 |
| 75 | NA | 0.95 | 0.20 | 1.00 | 0.24 | 1.00 | 0.26 | 0.00 | 0.53 | 1.00 | 0.25 | 1.55 | 0.06 | 0.07 | 0.62 |
| 485 | NA | 0.00 | 0.58 | 1.00 | 0.25 | 1.00 | 0.22 | 0.00 | 0.59 | 1.42 | 0.09 | 1.55 | 0.09 | 0.04 | 0.94 |
| 647 | NA | 1.42 | 0.06 | 1.35 | 0.07 | 1.41 | 0.08 | 1.42 | 0.09 | 1.00 | 0.29 | 1.54 | 0.08 | 0.06 | 0.64 |
| 724 | Stigmasterol | 1.00 | 0.26 | 1.51 | 0.03 | 0.00 | 0.62 | 1.00 | 0.27 | 1.39 | 0.07 | 1.51 | 0.08 | 0.08 | 0.13 |
| 111 | 2-Butenedioic acid (Fumaric acid) | 1.79 | 0.02 | 1.34 | 0.13 | 1.00 | 0.25 | 0.00 | 0.51 | 1.00 | 0.26 | 1.47 | 0.06 | 0.06 | 0.78 |
| 507 | NA | 1.13 | 0.13 | 1.00 | 0.28 | 1.36 | 0.11 | 0.00 | 0.55 | 1.37 | 0.11 | 1.44 | 0.10 | 0.07 | 0.63 |
| 630 | NA | 1.20 | 0.10 | 0.00 | 0.60 | 1.00 | 0.25 | 1.29 | 0.11 | 0.00 | 0.58 | 1.44 | 0.07 | 0.06 | 0.60 |
| 727 | NA | 1.00 | 0.22 | 1.00 | 0.25 | 0.00 | 0.62 | 1.00 | 0.20 | 1.17 | 0.09 | 1.43 | 0.07 | 0.04 | 0.80 |
| 211 | NA | 1.00 | 0.22 | 1.64 | 0.04 | 1.00 | 0.24 | 0.00 | 0.55 | 1.00 | 0.21 | 1.43 | 0.08 | 0.07 | 0.73 |
| 71 | NA | 0.08 | 0.39 | 1.00 | 0.25 | 0.00 | 0.53 | 0.00 | 0.56 | 0.00 | 0.56 | 1.42 | 0.13 | 0.03 | 1.00 |
| 425 | NA | 1.00 | 0.20 | 0.00 | 0.51 | 0.00 | 0.55 | 0.00 | 0.55 | 1.39 | 0.10 | 1.42 | 0.08 | 0.03 | 0.99 |
| 760 | NA | 1.42 | 0.08 | 1.00 | 0.27 | 0.00 | 0.53 | 1.00 | 0.27 | 0.00 | 0.56 | 1.42 | 0.08 | 0.03 | 0.98 |
| 410 | NA | 1.35 | 0.15 | 0.00 | 0.55 | 0.00 | 0.50 | 1.00 | 0.18 | 1.00 | 0.22 | 1.42 | 0.10 | 0.04 | 0.97 |
| 411 | NA | 1.30 | 0.14 | 0.00 | 0.56 | -1.00 | 0.81 | 2.10 | 0.01 | -0.19 | 0.60 | 1.42 | 0.09 | 0.13 | 0.23 |
| 179 | NA | 1.00 | 0.24 | 0.00 | 0.57 | 0.08 | 0.35 | 0.00 | 0.52 | 1.39 | 0.09 | 1.41 | 0.07 | 0.03 | 0.98 |
| 956 | NA | -1.00 | 0.81 | 0.00 | 0.51 | 1.58 | 0.07 | 1.00 | 0.22 | 1.42 | 0.11 | 1.41 | 0.11 | 0.07 | 0.88 |
| 605 | Nonacosane | 0.00 | 0.61 | 1.00 | 0.26 | 1.42 | 0.07 | 0.00 | 0.60 | 1.00 | 0.26 | 1.41 | 0.12 | 0.04 | 0.79 |
| 539 | NA | 1.42 | 0.09 | -0.38 | 0.67 | 0.00 | 0.52 | 1.00 | 0.25 | 0.00 | 0.55 | 1.40 | 0.11 | 0.06 | 0.80 |
| 744 | Stigmastanol | 1.00 | 0.26 | 1.42 | 0.04 | 1.00 | 0.21 | 0.00 | 0.62 | 0.00 | 0.66 | 1.40 | 0.10 | 0.05 | 0.52 |
| 50 | 1,2-Ethanedioic acid (Oxalic acid) | 0.00 | 0.53 | 1.00 | 0.28 | 1.00 | 0.23 | 1.00 | 0.20 | 1.00 | 0.26 | 1.40 | 0.11 | 0.05 | 0.90 |

| Cluster index | Putative Compound | Bagasse | Bagasse *p*-Value | Filter cake | Filter cake *p*-Value | Stems | Stems *p*-Value | Tops | Tops *p*-Value | Trash | Trash *p*-Value | Mean Decrease Accuracy | Mean Decrease Accuracy *p*-Value | Mean Decrease Gini | Mean Decrease Gini *p*-Value |
|---|---|---|---|---|---|---|---|---|---|---|---|---|---|---|---|
| 262 | NA | 1.35 | 0.12 | 1.00 | 0.26 | 0.00 | 0.56 | 0.00 | 0.57 | 1.00 | 0.25 | 1.39 | 0.11 | 0.05 | 0.92 |
| 523 | Sucrose | 1.00 | 0.23 | 0.00 | 0.60 | 1.00 | 0.30 | 1.00 | 0.28 | 1.00 | 0.22 | 1.38 | 0.12 | 0.01 | 0.99 |
| 499 | NA | 1.42 | 0.05 | 1.32 | 0.12 | 1.33 | 0.11 | 1.00 | 0.27 | 1.00 | 0.27 | 1.38 | 0.12 | 0.05 | 0.85 |
| 186 | NA | 0.00 | 0.55 | 1.00 | 0.26 | 1.00 | 0.20 | 0.00 | 0.49 | 0.00 | 0.51 | 1.38 | 0.09 | 0.01 | 1.00 |
| 275 | Fructose | 1.00 | 0.31 | 1.26 | 0.11 | 0.00 | 0.65 | 0.00 | 0.63 | 1.00 | 0.21 | 1.37 | 0.11 | 0.05 | 0.71 |
| 805 | NA | 0.00 | 0.56 | 1.42 | 0.05 | 0.00 | 0.52 | 0.00 | 0.55 | 1.00 | 0.28 | 1.36 | 0.11 | 0.03 | 0.97 |
| 806 | NA | 1.42 | 0.06 | 1.00 | 0.21 | 1.00 | 0.26 | 0.00 | 0.51 | 1.00 | 0.23 | 1.35 | 0.11 | 0.05 | 0.95 |
| 504 | NA | 1.40 | 0.09 | 1.28 | 0.12 | 0.00 | 0.53 | 0.00 | 0.50 | 0.00 | 0.48 | 1.35 | 0.11 | 0.05 | 0.94 |
| 611 | NA | 1.00 | 0.25 | 1.00 | 0.24 | 0.00 | 0.57 | 1.00 | 0.23 | 1.00 | 0.29 | 1.34 | 0.11 | 0.03 | 0.87 |
| 404 | NA | 1.00 | 0.21 | 0.00 | 0.59 | 1.00 | 0.23 | 0.00 | 0.51 | 0.00 | 0.55 | 1.33 | 0.11 | 0.03 | 0.97 |
| 603 | NA | 1.57 | 0.04 | -1.00 | 0.86 | 1.42 | 0.08 | 1.37 | 0.12 | 0.00 | 0.54 | 1.33 | 0.15 | 0.06 | 0.85 |
| 649 | NA | 1.33 | 0.12 | 0.00 | 0.59 | 0.00 | 0.57 | 1.32 | 0.13 | 0.00 | 0.58 | 1.32 | 0.13 | 0.05 | 0.84 |
| 621 | NA | 1.00 | 0.22 | 1.41 | 0.07 | 0.00 | 0.57 | 1.00 | 0.30 | 1.00 | 0.28 | 1.32 | 0.13 | 0.03 | 0.95 |
| 255 | Citric acid | 0.00 | 0.57 | 1.42 | 0.05 | 0.00 | 0.61 | 1.00 | 0.27 | 0.00 | 0.51 | 1.32 | 0.11 | 0.03 | 0.98 |
| 671 | NA | 1.00 | 0.23 | 0.00 | 0.64 | 0.00 | 0.62 | 1.24 | 0.12 | 1.00 | 0.29 | 1.32 | 0.10 | 0.04 | 0.72 |
| 924 | NA | 1.00 | 0.22 | 1.00 | 0.25 | 1.00 | 0.26 | 1.00 | 0.27 | 1.00 | 0.26 | 1.31 | 0.15 | 0.03 | 0.99 |
| 312 | NA | 1.09 | 0.12 | 1.13 | 0.11 | 1.00 | 0.26 | 0.00 | 0.58 | 0.00 | 0.63 | 1.30 | 0.11 | 0.06 | 0.47 |
| 116 | NA | 1.00 | 0.24 | 0.00 | 0.56 | 0.00 | 0.53 | 0.00 | 0.56 | 1.37 | 0.09 | 1.29 | 0.13 | 0.05 | 0.88 |
| 429 | Tricosane | 1.00 | 0.23 | 0.00 | 0.60 | 1.00 | 0.23 | -1.00 | 0.90 | 1.00 | 0.27 | 1.29 | 0.13 | 0.03 | 0.96 |
| 103 | 1,2,3-Butanetriol | 1.00 | 0.21 | 1.00 | 0.26 | 1.38 | 0.08 | 1.00 | 0.26 | 1.00 | 0.23 | 1.28 | 0.13 | 0.03 | 0.92 |
| 113 | 1,2,3-Butanetriol | 1.00 | 0.26 | 1.00 | 0.26 | 1.00 | 0.22 | 1.00 | 0.22 | 0.00 | 0.54 | 1.25 | 0.17 | 0.03 | 0.99 |
| 169 | NA | 1.42 | 0.10 | 1.00 | 0.25 | 1.00 | 0.25 | 1.00 | 0.21 | 0.00 | 0.52 | 1.24 | 0.15 | 0.04 | 0.98 |
| 548 | Heptacosane | 1.00 | 0.23 | 1.00 | 0.27 | 1.00 | 0.23 | 0.00 | 0.66 | 1.00 | 0.26 | 1.24 | 0.15 | 0.05 | 0.59 |
| 559 | NA | 1.29 | 0.14 | -0.07 | 0.61 | 1.41 | 0.10 | 0.00 | 0.54 | 0.00 | 0.57 | 1.24 | 0.13 | 0.05 | 0.86 |
| 454 | NA | 1.00 | 0.29 | 1.39 | 0.12 | 1.00 | 0.25 | 0.00 | 0.59 | 0.00 | 0.58 | 1.16 | 0.19 | 0.05 | 0.87 |
| 801 | NA | 1.37 | 0.11 | 1.00 | 0.22 | 0.00 | 0.53 | -1.00 | 0.81 | 1.00 | 0.22 | 1.14 | 0.15 | 0.04 | 0.95 |
| 256 | NA | 1.00 | 0.23 | 1.00 | 0.28 | -1.00 | 0.87 | 1.00 | 0.22 | 1.00 | 0.25 | 1.10 | 0.13 | 0.04 | 0.91 |
| 49 | 1,2-Ethanedioic acid (Oxalic acid) | 1.00 | 0.20 | 1.00 | 0.27 | 0.00 | 0.52 | 0.00 | 0.52 | -1.00 | 0.86 | 1.00 | 0.17 | 0.03 | 0.99 |
| 55 | 3-Hydroxypropanoic acid (Hydracrylic acid) | 1.00 | 0.26 | 0.00 | 0.60 | 0.00 | 0.55 | 0.00 | 0.50 | 0.00 | 0.55 | 1.00 | 0.16 | 0.01 | 1.00 |
| 70 | Propanedioic acid (Malonic acid) | 0.00 | 0.52 | 1.00 | 0.25 | 0.00 | 0.53 | 0.00 | 0.55 | 1.00 | 0.27 | 1.00 | 0.19 | 0.02 | 1.00 |
| 118 | NA | 0.00 | 0.50 | 0.00 | 0.55 | 1.00 | 0.23 | 1.00 | 0.29 | 0.00 | 0.57 | 1.00 | 0.23 | 0.03 | 0.99 |
| 165 | NA | 0.00 | 0.59 | 1.00 | 0.23 | 0.00 | 0.60 | 0.00 | 0.61 | 0.00 | 0.60 | 1.00 | 0.20 | 0.01 | 1.00 |
| 166 | NA | 1.00 | 0.23 | 0.00 | 0.54 | 1.00 | 0.23 | 0.00 | 0.56 | 0.00 | 0.51 | 1.00 | 0.15 | 0.01 | 1.00 |
| 191 | Arabinose | 0.00 | 0.56 | 1.00 | 0.26 | 0.00 | 0.62 | 0.00 | 0.63 | -1.00 | 0.86 | 1.00 | 0.16 | 0.03 | 0.95 |
| 230 | 3,6-Anhydrogalactose | 1.00 | 0.24 | 1.00 | 0.24 | 0.00 | 0.56 | -1.00 | 0.87 | 1.00 | 0.29 | 1.00 | 0.17 | 0.03 | 0.98 |
| 237 | NA | 0.00 | 0.61 | 0.00 | 0.61 | 1.00 | 0.23 | 0.00 | 0.51 | 0.00 | 0.57 | 1.00 | 0.20 | 0.01 | 1.00 |
| 280 | NA | 1.00 | 0.28 | 0.00 | 0.65 | 0.00 | 0.58 | 1.00 | 0.26 | 1.00 | 0.26 | 1.00 | 0.22 | 0.02 | 0.96 |
| 281 | Glucose | 0.00 | 0.54 | 1.00 | 0.20 | 0.00 | 0.54 | 0.00 | 0.59 | 0.00 | 0.58 | 1.00 | 0.17 | 0.01 | 1.00 |
| 351 | *Myo*-Inositol | 1.00 | 0.25 | 0.00 | 0.59 | 0.00 | 0.58 | 1.00 | 0.26 | 0.00 | 0.62 | 1.00 | 0.15 | 0.01 | 1.00 |
| 370 | Heptadecanoic acid (Margaric acid) | 1.00 | 0.26 | 0.00 | 0.55 | 0.00 | 0.60 | 0.00 | 0.60 | 1.00 | 0.23 | 1.00 | 0.18 | 0.01 | 1.00 |
| 383 | NA | 0.00 | 0.56 | 1.00 | 0.26 | 0.00 | 0.52 | 0.00 | 0.58 | 1.00 | 0.25 | 1.00 | 0.19 | 0.03 | 0.98 |
| 389 | NA | 1.00 | 0.24 | 1.00 | 0.27 | 0.00 | 0.59 | 0.00 | 0.54 | 0.00 | 0.58 | 1.00 | 0.15 | 0.03 | 0.98 |
| 423 | NA | 0.00 | 0.55 | 0.00 | 0.58 | 0.00 | 0.56 | 1.00 | 0.23 | 1.00 | 0.25 | 1.00 | 0.19 | 0.01 | 1.00 |
| 434 | NA | 1.00 | 0.26 | 1.00 | 0.24 | 1.00 | 0.24 | 0.00 | 0.58 | 0.00 | 0.63 | 1.00 | 0.20 | 0.03 | 0.97 |
| 463 | NA | 1.00 | 0.26 | 0.00 | 0.50 | 0.00 | 0.54 | 0.00 | 0.49 | 0.00 | 0.56 | 1.00 | 0.15 | 0.01 | 1.00 |
| 470 | NA | 1.00 | 0.23 | 0.00 | 0.53 | 1.00 | 0.23 | 0.00 | 0.52 | 0.00 | 0.52 | 1.00 | 0.19 | 0.01 | 1.00 |
| 477 | Eicosanoic acid (Arachidic acid) | 0.00 | 0.65 | 0.00 | 0.60 | 0.00 | 0.63 | 1.00 | 0.25 | -1.00 | 0.90 | 1.00 | 0.19 | 0.01 | 0.99 |
| 488 | NA | -1.00 | 0.84 | 1.00 | 0.21 | 1.00 | 0.21 | 0.00 | 0.58 | 0.00 | 0.55 | 1.00 | 0.16 | 0.03 | 0.99 |
| 520 | NA | 0.00 | 0.52 | 0.00 | 0.57 | 0.00 | 0.50 | 1.00 | 0.23 | 1.00 | 0.25 | 1.00 | 0.15 | 0.03 | 0.98 |
| 524 | Docosanoic acid (Behenic acid) | -1.00 | 0.89 | 1.00 | 0.23 | 1.00 | 0.27 | 0.00 | 0.67 | 1.00 | 0.25 | 1.00 | 0.17 | 0.03 | 0.84 |

| Cluster index | Putative Compound | Bagasse | Bagasse *p*-Value | Filter cake | Filter cake *p*-Value | Stems | Stems *p*-Value | Tops | Tops *p*-Value | Trash | Trash *p*-Value | Mean Decrease Accuracy | Mean Decrease Accuracy *p*-Value | Mean Decrease Gini | Mean Decrease Gini *p*-Value |
|---|---|---|---|---|---|---|---|---|---|---|---|---|---|---|---|
| 540 | NA | 0.00 | 0.51 | 1.00 | 0.25 | 0.00 | 0.55 | 0.00 | 0.56 | 1.00 | 0.25 | 1.00 | 0.18 | 0.03 | 0.99 |
| 555 | Trehalose | 0.00 | 0.56 | 1.00 | 0.27 | 0.00 | 0.58 | 0.00 | 0.57 | 0.00 | 0.60 | 1.00 | 0.22 | 0.03 | 0.96 |
| 564 | NA | 0.00 | 0.61 | 1.00 | 0.23 | 0.00 | 0.58 | 0.00 | 0.54 | 0.00 | 0.59 | 1.00 | 0.18 | 0.02 | 0.98 |
| 566 | NA | 1.00 | 0.28 | 1.00 | 0.27 | 0.00 | 0.54 | 0.00 | 0.51 | 0.00 | 0.56 | 1.00 | 0.20 | 0.05 | 0.86 |
| 569 | NA | 0.00 | 0.54 | 1.00 | 0.27 | 0.00 | 0.59 | 0.00 | 0.56 | 1.00 | 0.28 | 1.00 | 0.19 | 0.03 | 0.98 |
| 579 | NA | 0.00 | 0.53 | 0.00 | 0.57 | 0.00 | 0.51 | 0.00 | 0.50 | 1.00 | 0.28 | 1.00 | 0.19 | 0.01 | 1.00 |
| 582 | NA | 0.00 | 0.56 | -1.00 | 0.86 | 1.00 | 0.25 | 0.00 | 0.55 | 0.00 | 0.58 | 1.00 | 0.23 | 0.01 | 1.00 |
| 644 | Hexacosanoic acid (Cerotic acid) | 1.00 | 0.28 | 0.00 | 0.61 | 0.00 | 0.56 | 0.00 | 0.60 | 0.00 | 0.63 | 1.00 | 0.21 | 0.02 | 0.96 |
| 670 | NA | 1.00 | 0.31 | 1.00 | 0.26 | 1.00 | 0.25 | 0.00 | 0.57 | 0.00 | 0.61 | 1.00 | 0.23 | 0.02 | 0.98 |
| 700 | Octacosanoic acid (Montanic acid) | 1.00 | 0.28 | 0.00 | 0.63 | 1.00 | 0.25 | 0.00 | 0.64 | 0.00 | 0.59 | 1.00 | 0.18 | 0.01 | 1.00 |
| 757 | NA | 1.00 | 0.20 | 1.00 | 0.24 | 0.00 | 0.56 | 0.00 | 0.54 | 0.00 | 0.57 | 1.00 | 0.20 | 0.01 | 1.00 |
| 766 | NA | 1.00 | 0.24 | 1.00 | 0.29 | 0.00 | 0.56 | 0.00 | 0.51 | 0.00 | 0.54 | 1.00 | 0.17 | 0.03 | 1.00 |
| 779 | NA | 1.00 | 0.23 | 1.00 | 0.23 | 1.00 | 0.25 | 1.00 | 0.26 | 0.00 | 0.54 | 1.00 | 0.22 | 0.03 | 1.00 |
| 785 | Triacontanoic acid (Melissic acid) | 0.00 | 0.59 | 0.00 | 0.60 | 1.00 | 0.28 | 0.00 | 0.58 | 1.00 | 0.30 | 1.00 | 0.19 | 0.03 | 0.97 |
| 811 | NA | 0.00 | 0.58 | 0.00 | 0.55 | 0.00 | 0.55 | 0.00 | 0.52 | 1.00 | 0.29 | 1.00 | 0.19 | 0.01 | 0.99 |
| 854 | NA | 1.00 | 0.20 | -1.00 | 0.83 | 0.00 | 0.49 | 0.00 | 0.54 | 1.00 | 0.18 | 1.00 | 0.20 | 0.03 | 0.99 |
| 68 | NA | 1.00 | 0.22 | 1.00 | 0.26 | 1.00 | 0.29 | 0.00 | 0.53 | 0.00 | 0.58 | 1.00 | 0.25 | 0.02 | 0.99 |
| 94 | NA | 1.00 | 0.23 | 0.00 | 0.45 | 0.00 | 0.50 | 0.00 | 0.58 | 1.00 | 0.23 | 1.00 | 0.17 | 0.02 | 1.00 |
| 122 | 2-Hydroxybutanoic acid ($\alpha$-hydroxybutyric acid) | 1.00 | 0.23 | 0.00 | 0.52 | 0.00 | 0.54 | 0.00 | 0.52 | 0.00 | 0.51 | 1.00 | 0.23 | 0.01 | 1.00 |
| 136 | NA | 0.00 | 0.49 | 0.00 | 0.55 | 1.00 | 0.23 | 0.00 | 0.59 | 0.00 | 0.52 | 1.00 | 0.17 | 0.01 | 1.00 |
| 155 | Pyroglutamic acid | 1.00 | 0.24 | 0.00 | 0.66 | 0.00 | 0.63 | 1.00 | 0.23 | 0.00 | 0.67 | 1.00 | 0.20 | 0.01 | 1.00 |
| 215 | NA | 1.00 | 0.22 | 0.00 | 0.67 | 1.00 | 0.23 | 0.00 | 0.60 | 1.00 | 0.31 | 1.00 | 0.22 | 0.03 | 0.90 |
| 272 | NA | 0.00 | 0.56 | 0.00 | 0.52 | 0.00 | 0.54 | 0.00 | 0.55 | 1.00 | 0.23 | 1.00 | 0.20 | 0.01 | 1.00 |
| 273 | NA | 0.00 | 0.55 | 0.00 | 0.55 | 1.00 | 0.26 | 1.00 | 0.30 | 0.00 | 0.50 | 1.00 | 0.24 | 0.02 | 1.00 |
| 299 | NA | 0.00 | 0.59 | 1.00 | 0.21 | 0.00 | 0.55 | 0.00 | 0.57 | 0.00 | 0.58 | 1.00 | 0.17 | 0.00 | 1.00 |
| 300 | NA | 1.00 | 0.26 | 0.00 | 0.61 | 0.00 | 0.58 | 0.00 | 0.59 | 1.00 | 0.25 | 1.00 | 0.19 | 0.01 | 1.00 |
| 356 | *Myo*-Inositol | 0.00 | 0.57 | 1.00 | 0.25 | 0.00 | 0.52 | 0.00 | 0.60 | 0.00 | 0.54 | 1.00 | 0.21 | 0.01 | 1.00 |
| 366 | NA | -1.00 | 0.90 | 1.00 | 0.30 | 0.00 | 0.56 | 0.00 | 0.63 | 0.00 | 0.63 | 1.00 | 0.21 | 0.03 | 0.96 |
| 388 | NA | 1.00 | 0.19 | 0.00 | 0.49 | 1.00 | 0.21 | 0.00 | 0.57 | 0.00 | 0.56 | 1.00 | 0.18 | 0.03 | 1.00 |
| 390 | NA | 0.00 | 0.51 | 0.00 | 0.50 | 0.00 | 0.53 | 0.00 | 0.53 | 1.00 | 0.21 | 1.00 | 0.18 | 0.03 | 1.00 |
| 418 | NA | 1.00 | 0.23 | 1.00 | 0.24 | 0.00 | 0.49 | 0.00 | 0.51 | 0.00 | 0.52 | 1.00 | 0.19 | 0.01 | 1.00 |
| 467 | NA | 1.00 | 0.23 | 0.00 | 0.59 | 0.00 | 0.55 | 0.00 | 0.58 | 0.00 | 0.52 | 1.00 | 0.20 | 0.01 | 1.00 |
| 489 | NA | 0.00 | 0.54 | 1.00 | 0.25 | 0.00 | 0.56 | 0.00 | 0.53 | 0.00 | 0.52 | 1.00 | 0.16 | 0.01 | 1.00 |
| 492 | NA | 0.00 | 0.65 | 0.00 | 0.60 | 0.00 | 0.62 | 1.00 | 0.27 | 1.00 | 0.27 | 1.00 | 0.21 | 0.01 | 1.00 |
| 553 | NA | 1.00 | 0.23 | 0.00 | 0.54 | 0.00 | 0.48 | 0.00 | 0.54 | 1.00 | 0.24 | 1.00 | 0.17 | 0.01 | 1.00 |
| 668 | Chlorogenic acid | 1.00 | 0.24 | 1.00 | 0.27 | 0.00 | 0.58 | 0.00 | 0.56 | 0.00 | 0.56 | 1.00 | 0.17 | 0.02 | 0.99 |
| 683 | NA | 0.00 | 0.52 | 0.00 | 0.57 | 0.00 | 0.50 | 1.00 | 0.24 | 1.00 | 0.19 | 1.00 | 0.16 | 0.03 | 1.00 |
| 701 | NA | 0.00 | 0.62 | 1.00 | 0.22 | 1.00 | 0.26 | 0.00 | 0.57 | 0.00 | 0.59 | 1.00 | 0.19 | 0.00 | 1.00 |
| 815 | Hentriacontanoic acid (Hentriacontylic acid) | 1.00 | 0.24 | 1.00 | 0.26 | 0.00 | 0.57 | 1.00 | 0.26 | 0.00 | 0.57 | 1.00 | 0.21 | 0.01 | 1.00 |
| 844 | NA | 0.00 | 0.50 | 1.00 | 0.22 | 1.00 | 0.20 | 0.00 | 0.52 | 0.00 | 0.54 | 1.00 | 0.15 | 0.02 | 1.00 |
| 847 | NA | 1.00 | 0.27 | 0.00 | 0.58 | 0.00 | 0.55 | 1.00 | 0.25 | 0.00 | 0.51 | 1.00 | 0.21 | 0.02 | 1.00 |
| 904 | Butyl octacosyl ether | 0.00 | 0.55 | 0.00 | 0.54 | 0.00 | 0.57 | 1.00 | 0.25 | 0.00 | 0.53 | 1.00 | 0.21 | 0.03 | 0.99 |
| 594 | NA | 0.00 | 0.63 | 1.00 | 0.29 | -1.00 | 0.87 | 1.36 | 0.12 | 0.08 | 0.39 | 0.84 | 0.23 | 0.06 | 0.71 |
| 899 | NA | 1.00 | 0.21 | 1.39 | 0.08 | 0.16 | 0.38 | 0.00 | 0.55 | -1.00 | 0.86 | 0.77 | 0.23 | 0.07 | 0.76 |
| 257 | 3,4-Dihydroxybenzoic acid (Protocatechuic acid) | -1.00 | 0.83 | 0.00 | 0.57 | 0.00 | 0.53 | 1.00 | 0.24 | -0.68 | 0.71 | 0.33 | 0.36 | 0.03 | 0.98 |
| 706 | NA | -1.00 | 0.84 | 0.00 | 0.50 | 0.00 | 0.54 | 1.00 | 0.21 | 0.00 | 0.57 | 0.09 | 0.44 | 0.02 | 1.00 |
| 5 | Propane-1,2-diol (Propylene glycol) | 0.00 | 0.54 | 0.00 | 0.58 | 0.00 | 0.55 | 0.00 | 0.60 | 0.00 | 0.58 | 0.00 | 0.45 | 0.00 | 1.00 |
| 12 | NA | 0.00 | 0.55 | 0.00 | 0.57 | 0.00 | 0.57 | 0.00 | 0.56 | 0.00 | 0.54 | 0.00 | 0.49 | 0.00 | 1.00 |
| 14 | 2-Hydroxypropanoic acid (Lactic acid) | 0.00 | 0.57 | 0.00 | 0.56 | 0.00 | 0.51 | 0.00 | 0.58 | 0.00 | 0.54 | 0.00 | 0.45 | 0.00 | 1.00 |
| 16 | Butane-2,3-diol | 0.00 | 0.58 | 0.00 | 0.51 | 0.00 | 0.53 | 0.00 | 0.56 | 0.00 | 0.57 | 0.00 | 0.46 | 0.00 | 1.00 |

| Cluster index | Putative Compound | Bagasse | Bagasse *p*-Value | Filter cake | Filter cake *p*-Value | Stems | Stems *p*-Value | Tops | Tops *p*-Value | Trash | Trash *p*-Value | Mean Decrease Accuracy | Mean Decrease Accuracy *p*-Value | Mean Decrease Gini | Mean Decrease Gini *p*-Value |
|---|---|---|---|---|---|---|---|---|---|---|---|---|---|---|---|
| 20 | NA | 0.00 | 0.55 | 0.00 | 0.52 | 0.00 | 0.57 | 0.00 | 0.56 | 0.00 | 0.58 | 0.00 | 0.43 | 0.00 | 1.00 |
| 21 | 2-Hydroxypropanoic acid (Lactic Acid) | 0.00 | 0.61 | 0.00 | 0.54 | 0.00 | 0.58 | 0.00 | 0.62 | 0.00 | 0.60 | 0.00 | 0.52 | 0.00 | 1.00 |
| 27 | Hydroxyacetic acid (Glycolic acid) | 0.00 | 0.62 | 0.00 | 0.61 | 0.00 | 0.65 | 0.00 | 0.65 | 0.00 | 0.61 | 0.00 | 0.52 | 0.00 | 1.00 |
| 31 | 2-Oxopropanoic acid (Pyruvic acid) | 0.00 | 0.52 | 0.00 | 0.57 | 0.00 | 0.58 | 0.00 | 0.58 | 0.00 | 0.57 | 0.00 | 0.50 | 0.00 | 1.00 |
| 67 | NA | 0.00 | 0.56 | 0.00 | 0.54 | 0.00 | 0.57 | 0.00 | 0.56 | 0.00 | 0.53 | 0.00 | 0.45 | 0.00 | 1.00 |
| 73 | 3-Hydroxyisovaleric acid | 0.00 | 0.57 | 0.00 | 0.54 | 0.00 | 0.57 | 0.00 | 0.56 | 0.00 | 0.56 | 0.00 | 0.49 | 0.00 | 1.00 |
| 85 | NA | 0.00 | 0.56 | 0.00 | 0.55 | 0.00 | 0.53 | 0.00 | 0.50 | 0.00 | 0.51 | 0.00 | 0.45 | 0.00 | 1.00 |
| 90 | NA | 0.00 | 0.55 | 0.00 | 0.52 | 0.00 | 0.55 | 0.00 | 0.57 | 0.00 | 0.59 | 0.00 | 0.51 | 0.00 | 1.00 |
| 95 | Propane-1,2,3-triol (Glycerol) | 0.00 | 0.58 | 0.00 | 0.53 | 0.00 | 0.61 | 0.00 | 0.64 | 0.00 | 0.56 | 0.00 | 0.47 | 0.00 | 1.00 |
| 108 | 2,3-Diaminopropionic acid (3-Amino-alanine) | 0.00 | 0.54 | 0.00 | 0.54 | 0.00 | 0.54 | 0.00 | 0.58 | 0.00 | 0.57 | 0.00 | 0.46 | 0.00 | 1.00 |
| 120 | Threonine | 0.00 | 0.55 | 0.00 | 0.64 | 0.00 | 0.52 | 0.00 | 0.59 | 0.00 | 0.61 | 0.00 | 0.49 | 0.00 | 1.00 |
| 121 | NA | 0.00 | 0.54 | 0.00 | 0.55 | 0.00 | 0.50 | 0.00 | 0.56 | 0.00 | 0.57 | 0.00 | 0.46 | 0.00 | 1.00 |
| 131 | 3,4-Dihydroxybutanoic acid | 0.00 | 0.59 | 0.00 | 0.61 | 0.00 | 0.60 | 0.00 | 0.57 | 0.00 | 0.56 | 0.00 | 0.44 | 0.00 | 1.00 |
| 144 | Ascorbic acid | 0.00 | 0.61 | 0.00 | 0.62 | 0.00 | 0.57 | 0.00 | 0.61 | 0.00 | 0.56 | 0.00 | 0.49 | 0.00 | 1.00 |
| 149 | Threitol | 0.00 | 0.57 | 0.00 | 0.57 | 0.00 | 0.58 | 0.00 | 0.53 | 0.00 | 0.61 | 0.00 | 0.43 | 0.00 | 1.00 |
| 150 | Threitol | 0.00 | 0.62 | 0.00 | 0.63 | 0.00 | 0.65 | 0.00 | 0.58 | 0.00 | 0.56 | 0.00 | 0.50 | 0.00 | 1.00 |
| 156 | $\gamma$-Aminobutanoic acid (GABA) | 0.00 | 0.61 | 0.00 | 0.62 | 0.00 | 0.59 | 0.00 | 0.59 | 0.00 | 0.64 | 0.00 | 0.50 | 0.00 | 1.00 |
| 188 | NA | 0.00 | 0.61 | 0.00 | 0.58 | 0.00 | 0.59 | 0.00 | 0.54 | 0.00 | 0.60 | 0.00 | 0.50 | 0.00 | 1.00 |
| 198 | NA | 0.00 | 0.63 | 0.00 | 0.56 | 0.00 | 0.50 | 0.00 | 0.57 | 0.00 | 0.54 | 0.00 | 0.50 | 0.00 | 1.00 |
| 206 | NA | 0.00 | 0.59 | 0.00 | 0.57 | 0.00 | 0.54 | 0.00 | 0.58 | 0.00 | 0.59 | 0.00 | 0.46 | 0.00 | 1.00 |
| 208 | Xylitol | 0.00 | 0.60 | 0.00 | 0.54 | 0.00 | 0.59 | 0.00 | 0.63 | 0.00 | 0.55 | 0.00 | 0.46 | 0.00 | 1.00 |
| 242 | Vanillic acid | 0.00 | 0.57 | 0.00 | 0.58 | 0.00 | 0.52 | 0.00 | 0.57 | 0.00 | 0.59 | 0.00 | 0.45 | 0.00 | 1.00 |
| 267 | NA | 0.00 | 0.56 | 0.00 | 0.51 | 0.00 | 0.49 | 0.00 | 0.56 | 0.00 | 0.52 | 0.00 | 0.47 | 0.00 | 1.00 |
| 268 | NA | 0.00 | 0.58 | 0.00 | 0.66 | 0.00 | 0.58 | 0.00 | 0.62 | 0.00 | 0.63 | 0.00 | 0.51 | 0.00 | 1.00 |
| 271 | Adonitol | 0.00 | 0.61 | 0.00 | 0.54 | 0.00 | 0.56 | 0.00 | 0.51 | 0.00 | 0.55 | 0.00 | 0.45 | 0.00 | 1.00 |
| 287 | Talose | 0.00 | 0.64 | 0.00 | 0.66 | 0.00 | 0.65 | 0.00 | 0.58 | 0.00 | 0.62 | 0.00 | 0.50 | 0.00 | 1.00 |
| 294 | Glucitol | 0.00 | 0.58 | 0.00 | 0.66 | 0.00 | 0.64 | 0.00 | 0.63 | 0.00 | 0.63 | 0.00 | 0.50 | 0.00 | 1.00 |
| 316 | NA | 0.00 | 0.56 | 0.00 | 0.61 | 0.00 | 0.59 | 0.00 | 0.55 | 0.00 | 0.57 | 0.00 | 0.48 | 0.00 | 1.00 |
| 325 | 14-Methylhexadecanoic acid | 0.00 | 0.52 | 0.00 | 0.54 | 0.00 | 0.56 | 0.00 | 0.51 | 0.00 | 0.59 | 0.00 | 0.48 | 0.00 | 1.00 |
| 337 | NA | 0.00 | 0.61 | 0.00 | 0.62 | 0.00 | 0.57 | 0.00 | 0.52 | 0.00 | 0.52 | 0.00 | 0.48 | 0.00 | 1.00 |
| 338 | Hexadecanoic acid (Palmitic acid) | 0.00 | 0.61 | 0.00 | 0.64 | 0.00 | 0.59 | 0.00 | 0.60 | 0.00 | 0.58 | 0.00 | 0.52 | 0.00 | 1.00 |
| 344 | NA | 0.00 | 0.56 | 0.00 | 0.53 | 0.00 | 0.51 | 0.00 | 0.57 | 0.00 | 0.51 | 0.00 | 0.50 | 0.00 | 1.00 |
| 364 | Heneicosane | 0.00 | 0.56 | 0.00 | 0.54 | 0.00 | 0.55 | 0.00 | 0.56 | 0.00 | 0.59 | 0.00 | 0.49 | 0.00 | 1.00 |
| 368 | Mannitol | 0.00 | 0.56 | 0.00 | 0.58 | 0.00 | 0.51 | 0.00 | 0.55 | 0.00 | 0.57 | 0.00 | 0.51 | 0.00 | 1.00 |
| 374 | Methyl 2-hydroxystearate | 0.00 | 0.56 | 0.00 | 0.55 | 0.00 | 0.60 | 0.00 | 0.59 | 0.00 | 0.52 | 0.00 | 0.46 | 0.01 | 1.00 |
| 405 | galactinol | 0.00 | 0.56 | 0.00 | 0.57 | 0.00 | 0.59 | 0.00 | 0.57 | 0.00 | 0.58 | 0.00 | 0.54 | 0.00 | 1.00 |
| 428 | NA | 0.00 | 0.57 | 0.00 | 0.55 | 0.00 | 0.54 | 0.00 | 0.53 | 0.00 | 0.50 | 0.00 | 0.48 | 0.00 | 1.00 |
| 431 | NA | 0.00 | 0.55 | 0.00 | 0.56 | 0.00 | 0.51 | 0.00 | 0.52 | 0.00 | 0.55 | 0.00 | 0.47 | 0.00 | 1.00 |
| 432 | NA | 0.00 | 0.60 | 0.00 | 0.60 | 0.00 | 0.54 | 0.00 | 0.64 | 0.00 | 0.58 | 0.00 | 0.48 | 0.00 | 1.00 |
| 439 | NA | 0.00 | 0.51 | 0.00 | 0.56 | 0.00 | 0.51 | 0.00 | 0.52 | 0.00 | 0.56 | 0.00 | 0.48 | 0.00 | 1.00 |
| 440 | Nonadecanoic acid (Nonadecylic acid) | 0.00 | 0.60 | 0.00 | 0.62 | 0.00 | 0.59 | 0.00 | 0.58 | 0.00 | 0.65 | 0.00 | 0.47 | 0.00 | 1.00 |
| 441 | NA | 0.00 | 0.59 | 0.00 | 0.61 | 0.00 | 0.61 | 0.00 | 0.62 | 0.00 | 0.61 | 0.00 | 0.48 | 0.00 | 1.00 |
| 465 | NA | 0.00 | 0.55 | 0.00 | 0.57 | 0.00 | 0.58 | 0.00 | 0.54 | 0.00 | 0.56 | 0.00 | 0.48 | 0.00 | 1.00 |
| 486 | NA | 0.00 | 0.52 | 0.00 | 0.53 | 0.00 | 0.48 | 0.00 | 0.58 | 0.00 | 0.52 | 0.00 | 0.49 | 0.00 | 1.00 |
| 498 | NA | 0.00 | 0.57 | 0.00 | 0.56 | 0.00 | 0.58 | 0.00 | 0.58 | 0.00 | 0.56 | 0.00 | 0.51 | 0.00 | 1.00 |
| 503 | Heneicosanoic acid (Heneicosylic acid) | 0.00 | 0.66 | 0.00 | 0.68 | 0.00 | 0.59 | 0.00 | 0.65 | 0.00 | 0.63 | 0.00 | 0.59 | 0.01 | 1.00 |
| 512 | NA | 0.00 | 0.63 | 0.00 | 0.62 | 0.00 | 0.59 | 0.00 | 0.54 | 0.00 | 0.59 | 0.00 | 0.53 | 0.00 | 1.00 |
| 516 | NA | 0.00 | 0.57 | 0.00 | 0.56 | 0.00 | 0.56 | -1.00 | 0.88 | 1.00 | 0.25 | 0.00 | 0.46 | 0.01 | 1.00 |
| 519 | NA | 0.00 | 0.57 | 0.00 | 0.55 | 0.00 | 0.55 | 0.00 | 0.53 | 0.00 | 0.55 | 0.00 | 0.48 | 0.00 | 1.00 |
| 526 | Glucose-1-phosphate | 0.00 | 0.51 | 0.00 | 0.51 | 0.00 | 0.46 | 0.00 | 0.50 | 0.00 | 0.57 | 0.00 | 0.45 | 0.00 | 1.00 |

| Cluster index | Putative Compound | Bagasse | Bagasse *p*-Value | Filter cake | Filter cake *p*-Value | Stems | Stems *p*-Value | Tops | Tops *p*-Value | Trash | Trash *p*-Value | Mean Decrease Accuracy | Mean Decrease Accuracy *p*-Value | Mean Decrease Gini | Mean Decrease Gini *p*-Value |
|---|---|---|---|---|---|---|---|---|---|---|---|---|---|---|---|
| 528 | NA | 0.00 | 0.54 | 0.00 | 0.52 | 0.00 | 0.52 | 0.00 | 0.53 | 0.00 | 0.56 | 0.00 | 0.46 | 0.00 | 1.00 |
| 535 | NA | 0.00 | 0.55 | 0.00 | 0.57 | 0.00 | 0.57 | 0.00 | 0.54 | 0.00 | 0.55 | 0.00 | 0.49 | 0.00 | 1.00 |
| 537 | Mannobiose | 0.00 | 0.55 | 0.00 | 0.54 | 0.00 | 0.59 | 0.00 | 0.47 | 0.00 | 0.59 | 0.00 | 0.50 | 0.00 | 1.00 |
| 547 | NA | 0.00 | 0.53 | 0.00 | 0.52 | 0.00 | 0.50 | 0.00 | 0.53 | 0.00 | 0.58 | 0.00 | 0.45 | 0.00 | 1.00 |
| 551 | NA | 0.00 | 0.56 | 0.00 | 0.55 | 0.00 | 0.57 | 0.00 | 0.64 | 0.00 | 0.62 | 0.00 | 0.47 | 0.00 | 1.00 |
| 552 | NA | 0.00 | 0.54 | 0.00 | 0.50 | 0.00 | 0.57 | 0.00 | 0.61 | 0.00 | 0.58 | 0.00 | 0.45 | 0.00 | 1.00 |
| 554 | NA | 0.00 | 0.53 | 0.00 | 0.52 | 0.00 | 0.49 | 0.00 | 0.56 | 0.00 | 0.51 | 0.00 | 0.51 | 0.00 | 1.00 |
| 556 | Tricosanoic acid (Tricosylic acid) | 0.00 | 0.59 | 0.00 | 0.61 | 0.00 | 0.64 | 0.00 | 0.61 | 0.00 | 0.64 | 0.00 | 0.47 | 0.00 | 1.00 |
| 562 | NA | 0.00 | 0.50 | 0.00 | 0.50 | 0.00 | 0.46 | 0.00 | 0.54 | 0.00 | 0.50 | 0.00 | 0.39 | 0.00 | 1.00 |
| 565 | NA | 0.00 | 0.53 | 0.00 | 0.56 | 0.00 | 0.50 | 0.00 | 0.51 | 0.00 | 0.55 | 0.00 | 0.43 | 0.00 | 1.00 |
| 583 | NA | 0.00 | 0.55 | 0.00 | 0.55 | 0.00 | 0.54 | 0.00 | 0.56 | 0.00 | 0.55 | 0.00 | 0.47 | 0.00 | 1.00 |
| 584 | (*Z*)-13-Docosenamide | 0.00 | 0.67 | 0.00 | 0.59 | 0.00 | 0.59 | 0.00 | 0.59 | 0.00 | 0.64 | 0.00 | 0.53 | 0.01 | 1.00 |
| 586 | Tetracosanoic acid (Lignoceric acid) | 0.00 | 0.59 | 0.00 | 0.66 | 0.00 | 0.61 | 0.00 | 0.64 | 0.00 | 0.66 | 0.00 | 0.50 | 0.00 | 1.00 |
| 589 | NA | 0.00 | 0.48 | 0.00 | 0.48 | 0.00 | 0.51 | 0.00 | 0.51 | 0.00 | 0.52 | 0.00 | 0.46 | 0.00 | 1.00 |
| 593 | Melibiose | 0.00 | 0.54 | 0.00 | 0.56 | 0.00 | 0.52 | 0.00 | 0.57 | 0.00 | 0.53 | 0.00 | 0.52 | 0.00 | 1.00 |
| 598 | NA | 0.00 | 0.55 | 0.00 | 0.51 | 0.00 | 0.57 | 0.00 | 0.55 | 0.00 | 0.56 | 0.00 | 0.49 | 0.00 | 1.00 |
| 600 | NA | 0.00 | 0.53 | 0.00 | 0.56 | 0.00 | 0.63 | 0.00 | 0.56 | 0.00 | 0.64 | 0.00 | 0.52 | 0.00 | 1.00 |
| 602 | NA | 0.00 | 0.50 | 0.00 | 0.58 | 0.00 | 0.50 | 0.00 | 0.58 | 0.00 | 0.58 | 0.00 | 0.48 | 0.00 | 1.00 |
| 612 | NA | 0.00 | 0.58 | 0.00 | 0.53 | 0.00 | 0.56 | 0.00 | 0.58 | 0.00 | 0.58 | 0.00 | 0.42 | 0.00 | 1.00 |
| 613 | Pentacosanoic acid (Pentacosylic acid) | 0.00 | 0.64 | 0.00 | 0.62 | 0.00 | 0.65 | 0.00 | 0.64 | 0.00 | 0.60 | 0.00 | 0.50 | 0.00 | 1.00 |
| 617 | NA | 0.00 | 0.56 | 0.00 | 0.57 | 0.00 | 0.61 | 0.00 | 0.53 | 0.00 | 0.57 | 0.00 | 0.50 | 0.00 | 1.00 |
| 618 | NA | 0.00 | 0.52 | 0.00 | 0.60 | 0.00 | 0.53 | 0.00 | 0.58 | 0.00 | 0.56 | 0.00 | 0.51 | 0.00 | 1.00 |
| 619 | NA | 0.00 | 0.52 | 0.00 | 0.54 | 0.00 | 0.56 | 0.00 | 0.55 | 0.00 | 0.49 | 0.00 | 0.47 | 0.00 | 1.00 |
| 622 | NA | 0.00 | 0.51 | 0.00 | 0.50 | 0.00 | 0.51 | 0.00 | 0.53 | 0.00 | 0.48 | 0.00 | 0.47 | 0.00 | 1.00 |
| 631 | NA | 0.00 | 0.51 | 0.00 | 0.55 | 0.00 | 0.52 | 0.00 | 0.58 | 0.00 | 0.51 | 0.00 | 0.50 | 0.00 | 1.00 |
| 638 | NA | 0.00 | 0.54 | 0.00 | 0.52 | 0.00 | 0.48 | 0.00 | 0.53 | 0.00 | 0.56 | 0.00 | 0.48 | 0.00 | 1.00 |
| 658 | NA | 0.00 | 0.56 | 0.00 | 0.58 | 0.00 | 0.52 | 0.00 | 0.56 | 0.00 | 0.55 | 0.00 | 0.44 | 0.00 | 1.00 |
| 659 | NA | 0.00 | 0.53 | 0.00 | 0.53 | 0.00 | 0.60 | 0.00 | 0.61 | 0.00 | 0.52 | 0.00 | 0.49 | 0.00 | 1.00 |
| 674 | NA | 0.00 | 0.54 | 0.00 | 0.48 | 0.00 | 0.52 | 0.00 | 0.52 | 0.00 | 0.54 | 0.00 | 0.50 | 0.00 | 1.00 |
| 680 | NA | 0.00 | 0.52 | 0.00 | 0.57 | 0.00 | 0.57 | 0.00 | 0.56 | 0.00 | 0.53 | 0.00 | 0.48 | 0.00 | 1.00 |
| 687 | NA | 0.00 | 0.46 | 0.00 | 0.53 | 0.00 | 0.52 | 0.00 | 0.53 | 0.00 | 0.52 | 0.00 | 0.43 | 0.00 | 1.00 |
| 697 | NA | 0.00 | 0.50 | 0.00 | 0.58 | 0.00 | 0.51 | 0.00 | 0.50 | 0.00 | 0.49 | 0.00 | 0.45 | 0.00 | 1.00 |
| 710 | Heptacosanal | 0.00 | 0.55 | 0.00 | 0.56 | 0.00 | 0.62 | 0.00 | 0.58 | 0.00 | 0.55 | 0.00 | 0.49 | 0.00 | 1.00 |
| 711 | 5-Methoxysalicylic acid | 0.00 | 0.60 | 0.00 | 0.54 | 0.00 | 0.51 | 0.00 | 0.58 | 0.00 | 0.63 | 0.00 | 0.51 | 0.00 | 1.00 |
| 738 | NA | 0.00 | 0.60 | 0.00 | 0.58 | 0.00 | 0.60 | 0.00 | 0.60 | 0.00 | 0.54 | 0.00 | 0.49 | 0.00 | 1.00 |
| 741 | *β*-Sitosterol | 0.00 | 0.62 | 0.00 | 0.63 | 0.00 | 0.66 | 0.00 | 0.64 | 0.00 | 0.66 | 0.00 | 0.52 | 0.00 | 1.00 |
| 758 | NA | 0.00 | 0.54 | 0.00 | 0.55 | 0.00 | 0.61 | 0.00 | 0.58 | 0.00 | 0.60 | 0.00 | 0.53 | 0.00 | 1.00 |
| 761 | NA | 0.00 | 0.55 | 0.00 | 0.57 | 0.00 | 0.60 | 0.00 | 0.58 | 0.00 | 0.51 | 0.00 | 0.46 | 0.01 | 1.00 |
| 794 | NA | 0.00 | 0.53 | 0.00 | 0.55 | 0.00 | 0.49 | 0.00 | 0.55 | 0.00 | 0.51 | 0.00 | 0.48 | 0.00 | 1.00 |
| 796 | NA | 0.00 | 0.50 | 0.00 | 0.57 | 0.00 | 0.59 | 0.00 | 0.58 | 0.00 | 0.57 | 0.00 | 0.48 | 0.00 | 1.00 |
| 804 | NA | 0.00 | 0.55 | 0.00 | 0.55 | 0.00 | 0.55 | 0.00 | 0.55 | 0.00 | 0.60 | 0.00 | 0.54 | 0.00 | 1.00 |
| 814 | NA | 0.00 | 0.62 | 0.00 | 0.61 | 0.00 | 0.60 | 0.00 | 0.61 | 0.00 | 0.58 | 0.00 | 0.51 | 0.00 | 1.00 |
| 830 | NA | 0.00 | 0.60 | 0.00 | 0.56 | 0.00 | 0.58 | 0.00 | 0.57 | 0.00 | 0.57 | 0.00 | 0.48 | 0.00 | 1.00 |
| 848 | NA | 0.00 | 0.60 | 0.00 | 0.55 | 0.00 | 0.52 | 0.00 | 0.50 | 0.00 | 0.52 | 0.00 | 0.47 | 0.00 | 1.00 |
| 863 | NA | 0.00 | 0.52 | 0.00 | 0.53 | 0.00 | 0.50 | 0.00 | 0.59 | 0.00 | 0.56 | 0.00 | 0.48 | 0.00 | 1.00 |
| 865 | NA | 0.00 | 0.55 | 0.00 | 0.55 | 0.00 | 0.55 | 0.00 | 0.56 | 0.00 | 0.53 | 0.00 | 0.47 | 0.00 | 1.00 |
| 882 | NA | 0.00 | 0.59 | 0.00 | 0.48 | 0.00 | 0.59 | 0.00 | 0.56 | 0.00 | 0.53 | 0.00 | 0.49 | 0.00 | 1.00 |
| 887 | NA | 0.00 | 0.50 | 0.00 | 0.51 | 0.00 | 0.51 | 0.00 | 0.49 | 0.00 | 0.56 | 0.00 | 0.48 | 0.00 | 1.00 |
| 917 | NA | 0.00 | 0.49 | 0.00 | 0.54 | 0.00 | 0.50 | 0.00 | 0.55 | 0.00 | 0.50 | 0.00 | 0.47 | 0.00 | 1.00 |
| 918 | NA | 0.00 | 0.45 | 0.00 | 0.50 | 0.00 | 0.50 | 0.00 | 0.50 | 0.00 | 0.53 | 0.00 | 0.44 | 0.01 | 1.00 |

| Cluster index | Putative Compound | Bagasse | Bagasse *p*-Value | Filter cake | Filter cake *p*-Value | Stems | Stems *p*-Value | Tops | Tops *p*-Value | Trash | Trash *p*-Value | Mean Decrease Accuracy | Mean Decrease Accuracy *p*-Value | Mean Decrease Gini | Mean Decrease Gini *p*-Value |
|---|---|---|---|---|---|---|---|---|---|---|---|---|---|---|---|
| 925 | NA | 0.00 | 0.50 | 0.00 | 0.56 | 0.00 | 0.53 | 0.00 | 0.52 | 0.00 | 0.53 | 0.00 | 0.50 | 0.00 | 1.00 |
| 929 | NA | 0.00 | 0.52 | 0.00 | 0.47 | 0.00 | 0.48 | 0.00 | 0.52 | 0.00 | 0.53 | 0.00 | 0.48 | 0.00 | 1.00 |
| 947 | NA | 0.00 | 0.55 | 0.00 | 0.57 | 0.00 | 0.47 | 0.00 | 0.53 | 0.00 | 0.55 | 0.00 | 0.44 | 0.00 | 1.00 |
| 948 | NA | 0.00 | 0.51 | 0.00 | 0.57 | 0.00 | 0.50 | 0.00 | 0.49 | 0.00 | 0.49 | 0.00 | 0.45 | 0.00 | 1.00 |
| 767 | NA | 0.00 | 0.51 | 0.00 | 0.50 | 0.00 | 0.55 | -1.00 | 0.82 | 1.00 | 0.23 | -0.05 | 0.49 | 0.01 | 1.00 |
| 295 | NA | -1.00 | 0.87 | 0.00 | 0.60 | 0.00 | 0.60 | 0.00 | 0.61 | 0.00 | 0.63 | -1.00 | 0.83 | 0.01 | 1.00 |
| 590 | NA | -1.00 | 0.88 | 0.00 | 0.53 | 0.00 | 0.53 | 0.00 | 0.50 | 0.00 | 0.53 | -1.00 | 0.79 | 0.02 | 1.00 |
| 845 | NA | -1.00 | 0.83 | -1.00 | 0.84 | 0.00 | 0.56 | 0.00 | 0.52 | 0.00 | 0.58 | -1.00 | 0.81 | 0.01 | 1.00 |
| 239 | NA | 0.00 | 0.51 | 0.00 | 0.53 | 0.00 | 0.57 | 0.00 | 0.50 | -1.00 | 0.82 | -1.00 | 0.79 | 0.01 | 1.00 |
| 641 | NA | 0.00 | 0.54 | 0.00 | 0.54 | 0.00 | 0.53 | 0.00 | 0.53 | -1.00 | 0.84 | -1.00 | 0.80 | 0.01 | 1.00 |
| 879 | NA | -1.00 | 0.81 | 0.00 | 0.51 | 0.00 | 0.51 | 0.00 | 0.50 | 0.00 | 0.51 | -1.00 | 0.77 | 0.02 | 1.00 |

**Table S8. Putative Metabolites in the Heatmap of Figure 7 with Corresponding Cluster Indices**

| Feature | Cluster |
|---|---|
| 270_Tetradecanoic acid | cluster2 |
| 297_*p*-Coumaric acid | cluster2 |
| 83_Urea | cluster2 |
| 234_Ribonic acid | cluster2 |
| 27_Glycolic acid | cluster2 |
| 109_Glyceric acid | cluster2 |
| 21_Lactic Acid | cluster2 |
| 560_1-Oleoylglycerol | cluster2 |
| 403_Octadecanoic acid | cluster2 |
| 392_9-Octadecenoic acid | cluster2 |
| 338_Hexadecanoic acid | cluster2 |
| 370_Heptadecanoic acid | cluster2 |
| 477_Eicosanoic acid | cluster2 |
| 208_Xylitol | cluster1 |
| 150_Threitol | cluster1 |
| 95_Glycerol | cluster1 |
| 191_Arabinose | cluster1 |
| 275_Fructose | cluster1 |
| 287_Talose | cluster1 |
| 292_Mannose | cluster1 |
| 523_Sucrose | cluster1 |
| 294_Glucitol | cluster1 |
| 228_Aconitic acid | cluster1 |
| 351_Inositol | cluster1 |
| 281_Glucose | cluster1 |
| 255_Citric acid | cluster1 |
| 317_Gluconic acid | cluster1 |
| 555_Tahalose | cluster1 |
| 644_Hexacosanoic acid | cluster3 |
| 676_1-Octacosanol | cluster3 |
| 614_1-Hexacosanol | cluster3 |
| 548_Heptacosane | cluster3 |
| 724_Stigmasterol | cluster3 |
| 713_Campesterol | cluster3 |
| 741_*β*-Sitosterol | cluster3 |
| 744_Stigmastanol | cluster3 |

| Feature | Cluster |
|---|---|
| 605_Nonacosane | cluster3 |
| 817_1-Dotriacontanol | cluster4 |
| 293_Pentadecanoic acid | cluster4 |
| 378_Phytol | cluster4 |
| 740_1-Triacontanol | cluster4 |
| 785_Triacontanoic acid | cluster4 |
| 700_Octacosanoic acid | cluster4 |
| 586_Tetracosanoic acid | cluster4 |
| 524_Docosanoic acid | cluster4 |
| 556_Tricosanoic acid | cluster4 |
| 798_Lanosterol | cluster4 |
| 795_Glutinol | cluster4 |
| 667_Hentriacontane | cluster4 |
| 901_1-Tetratriacontanol | cluster4 |

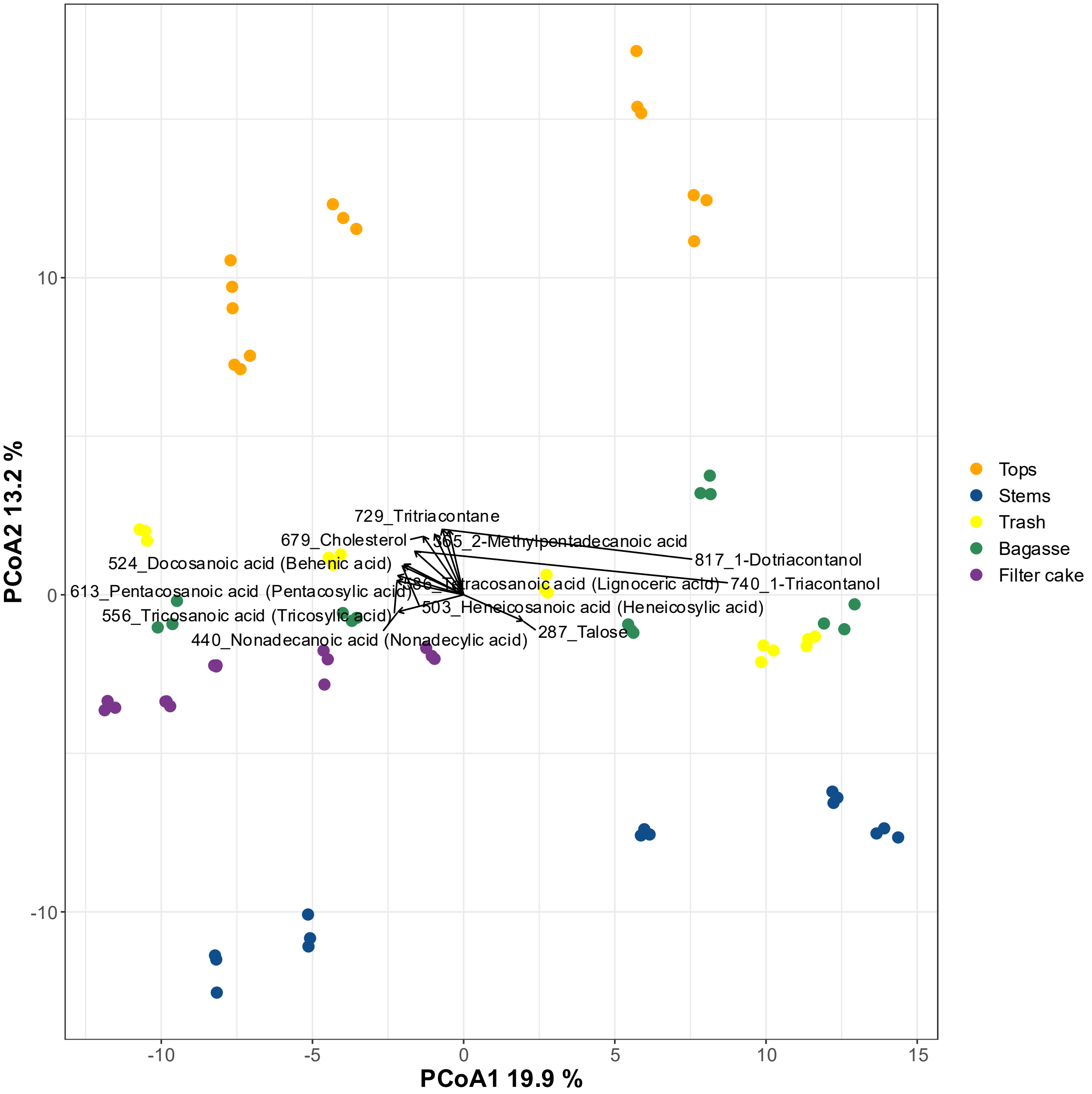


**Figure S1.** PCoA biplot showing metabolites significantly associated with the ordination axes based on envfit analysis. Arrows indicate the direction and strength of metabolite correlations with the PCoA axes, highlighting compounds contributing to the separation of sugarcane biomass types.

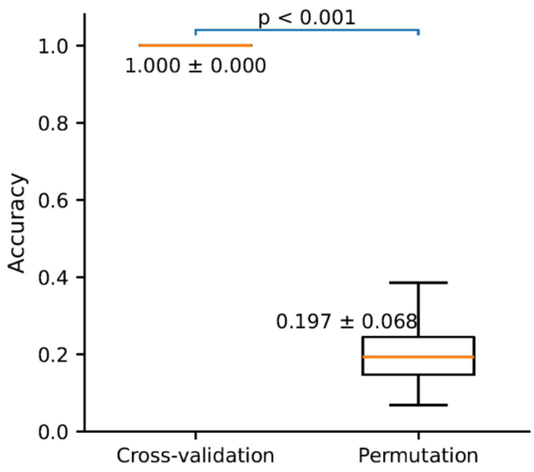


**Figure S2.** Comparison of random forest classification accuracy from repeated cross-validation and permutation testing. Cross-validation shows consistently high accuracy, whereas permutation accuracy approaches the random expectation (~0.02), confirming the statistical robustness of the model.
